# Supplementary material for: Localized effect of treated wastewater effluent on the resistome of an urban watershed
Source: Gigascience. 2020 Nov 19;9(11):giaa125. doi: 10.1093/gigascience/giaa125 (PMC7677451; doi:10.1093/gigascience/giaa125)
Supplement: giaa125_GIGA-D-20-00120_Revision_2 [file giaa125_giga-d-20-00120_revision_2.pdf]

GigaScience

Localized effect of treated wastewater effluent on the resistome of an urban watershed

--Manuscript Draft--

**Manuscript Number:**	GIGA-D-20-00120R2
**Full Title:**	Localized effect of treated wastewater effluent on the resistome of an urban watershed
**Article Type:**	Research
**Funding Information:**	Centers for Disease Control and Prevention (200-2016-91949) — Dr. James VanDerslice
**Abstract:**	Background<br>Wastewater treatment is an essential tool for maintaining water quality in urban environments. While the treatment of wastewater can remove most bacterial cells, some will inevitably survive treatment to be released into natural environments. Previous studies have investigated antibiotic resistance within wastewater treatment plants, but few studies have explored how a river's complete set of antibiotic resistance genes (the 'resistome') is affected by the release of treated effluent into surface waters.<br>Results<br>Here we used high-throughput, deep metagenomic sequencing to investigate the impact of treated wastewater effluent on the resistome of an urban river and the downstream distribution of effluent-associated antibiotic resistance genes and mobile genetic elements. Treated effluent release was found to be associated with increased abundance and diversity of antibiotic resistance genes and mobile genetic elements. The impact of wastewater discharge on the river's resistome diminished with increasing distance from effluent discharge points. The resistome at river locations that were not immediately downstream from any wastewater discharge points were dominated by a single integron carrying genes associated with resistance to sulfonamides and quaternary ammonium compounds.<br>Conclusions<br>Our study documents variations in the resistome of an urban watershed from headwaters to a major confluence in an urban center. Greater abundances and diversity of antibiotic resistance genes are associated with human fecal contamination in river surface water, but the fecal contamination effect appears to be localized, with little measurable impact in downstream waters. The diverse composition of antibiotic resistance genes throughout the watershed suggests the influence of multiple environmental and biological factors.
**Corresponding Author:**	Christopher Neil Thornton, MS<br>University of Utah<br>Salt Lake City, Utah UNITED STATES
**Corresponding Author Secondary Information:**	
**Corresponding Author's Institution:**	University of Utah
**Corresponding Author's Secondary Institution:**	
**First Author:**	Christopher Neil Thornton, MS
**First Author Secondary Information:**	
**Order of Authors:**	Christopher Neil Thornton, MS
	James VanDerslice, PhD
	Windy Tanner, PhD
	William Brazelton, PhD
**Order of Authors Secondary Information:**	
**Response to Reviewers:**	Dear GigaScience Editorial Office,<br>We appreciate the reviewers' continued efforts to improve the paper, and believe that

	we have now adequately addressed their final concerns.<br><br>Reviewer #1 (Comments for the Author):<br>The authors have revised the manuscript and replied to all concerns raised by the reviewers.<br><br>1. The manuscript has improved, but I still have one concern about the normalisation that I raised already on the first round of review (point #6). As reviewer #2 also raised the same issue, I think it should be corrected in all figures and analyses. Now the authors have only removed the description of normalisation from the methods, but kept it in figures and analyses.<br><br>Response:<br>We have updated all of the relevant figures, tables, and statistical test results to reflect the new normalization requested by the reviewers. Previously these results were reported in the supplemental material, and they are now reflected in the main text, as requested.<br><br>Reviewer #2 (Comments for the Author):<br>The authors have addressed almost all my concerns in their revised manuscript. However, there are two instances where I think they could still improve the clarity of the manuscript and where I feel that they skirted my comments a bit.<br><br>1. In Table 1, it is still not clear how prevalence is counted, given that there were only 24 sites. How can the prevalence then be >24?<br><br>Response:<br>The numbers in the table reflect the prevalence on a per sample basis, as three samples were collected at each sampling site. However, we realize that it would be informative to also provide the site prevalence, so we have updated the table to include those numbers as well.<br><br>2. Under "Identification and quantification of ARGs and MGEs", exactly what was regarded a "high sequence similarity threshold" (see my earlier comments)? It should not be hard to provide this number.<br><br>Response:<br>The revised manuscript states:<br>"Homologs of known antibiotic resistance determinants were identified from the predicted protein sequences using AMRFinder v1.04 with default settings [21]. ARGs detected through AMRFinder with family type equal to "equivalog" or "subfamily" were excluded to ensure that only the highest confidence results were reported."<br><br>There is no single answer to the reviewer's question because AMRFinder uses a tiered decision-making process, as explained in the AMRFinder documentation:<br>BLASTP is run with the -evalue 1e-20 -comp_based_stats 0 options against the AMR gene database described above. Exact BLAST matches over the full length of the reference protein are reported. If there is no exact match, then the following rules are applied unless overridden by a curated blast cutoff: Matches with < 90% identity or with < 50% coverage of the protein are dropped. If the hit is to a fusion protein than at least 90% of the protein must be covered. Curated blast cutoffs override these defaults. A BLAST match to a reference protein is removed if it is covered by another BLAST match which has more identical residues or the same number of identical residues, but to a longer reference protein. A single match is chosen as the best of what remains sorting by the following criteria in order (1) if it is exact; (2) has more identical residues; (3) hits a shorter protein; or (4) the gene symbol comes first in alphabetical order.<br><br>Therefore, we have added a sentence referring the reader to the AMRFinder documentation if the reader would like to know more about how ARGs are detected by AMRFinder.
**Additional Information:**	
**Question**	**Response**

Are you submitting this manuscript to a special series or article collection?	No
**Experimental design and statistics**<br><br>Full details of the experimental design and statistical methods used should be given in the Methods section, as detailed in our Minimum Standards Reporting Checklist. Information essential to interpreting the data presented should be made available in the figure legends.<br><br>Have you included all the information requested in your manuscript?	Yes
**Resources**<br><br>A description of all resources used, including antibodies, cell lines, animals and software tools, with enough information to allow them to be uniquely identified, should be included in the Methods section. Authors are strongly encouraged to cite Research Resource Identifiers (RRIDs) for antibodies, model organisms and tools, where possible.<br><br>Have you included the information requested as detailed in our Minimum Standards Reporting Checklist?	Yes
**Availability of data and materials**<br><br>All datasets and code on which the conclusions of the paper rely must be either included in your submission or deposited in publicly available repositories (where available and ethically appropriate), referencing such data using a unique identifier in the references and in the "Availability of Data and Materials" section of your manuscript.<br><br>Have you have met the above requirement as detailed in our Minimum	Yes

Standards Reporting Checklist?	

Powered by Editorial Manager® and ProduXion Manager® from Aries Systems Corporation

LaTeX file

Click here to access/download;LaTeX - Main Document (TeX file);main.tex

Placeholder for OUP logo oup.pdf

Placeholder for journal logo gigascience-logo.pdf

GigaScience, 2017, 1–11

doi: xx.xxxx/xxxx
Manuscript in Preparation
Paper

PAPER

Localized effect of treated wastewater effluent on the resistome of an urban watershed

Christopher N. Thornton[1,*], Windy D. Tanner[2], James A. VanDerslice[2] and William J. Brazelton[1,*]

[1]School of Biological Sciences, University of Utah, Salt Lake City, UT, USA and [2]Department of Family and Preventive Medicine, University of Utah, Salt Lake City, UT, USA

*christopher.thornton@utah.edu; william.brazelton@utah.edu

Abstract

Background
Wastewater treatment is an essential tool for maintaining water quality in urban environments. While the treatment of wastewater can remove most bacterial cells, some will inevitably survive treatment to be released into natural environments. Previous studies have investigated antibiotic resistance within wastewater treatment plants, but few studies have explored how a river's complete set of antibiotic resistance genes (the 'resistome') is affected by the release of treated effluent into surface waters.
Results
Here we used high-throughput, deep metagenomic sequencing to investigate the impact of treated wastewater effluent on the resistome of an urban river and the downstream distribution of effluent-associated antibiotic resistance genes and mobile genetic elements. Treated effluent release was found to be associated with increased abundance and diversity of antibiotic resistance genes and mobile genetic elements. The impact of wastewater discharge on the river's resistome diminished with increasing distance from effluent discharge points. The resistome at river locations that were not immediately downstream from any wastewater discharge points were dominated by a single integron carrying genes associated with resistance to sulfonamides and quaternary ammonium compounds.
Conclusions
Our study documents variations in the resistome of an urban watershed from headwaters to a major confluence in an urban center. Greater abundances and diversity of antibiotic resistance genes are associated with human fecal contamination in river surface water, but the fecal contamination effect appears to be localized, with little measurable impact in downstream waters. The diverse composition of antibiotic resistance genes throughout the watershed suggests the influence of multiple environmental and biological factors.

Key words: antibiotic resistance, wastewater, metagenome, watershed, pollution

Background

The growing public health crisis caused by the emergence and spread of antibiotic resistance is now recognized as a global problem with a complex interplay of environmental, biological, and clinical factors. Research on antimicrobial resistance has historically been focused on human pathogens, with hospitals and other clinical settings thought to be the primary source for the dissemination and evolution of antibiotic resistance. However, in part due to increased reporting of community-acquired, antibiotic-resistant infections [? ?], researchers have started to broaden their focus to include the role of natural environments as possible reservoirs of antimicrobial resistance and as settings for the evolution of new resistance determi-

Compiled on: September 16, 2020.
Draft manuscript prepared by the author.

nants.

Antimicrobial resistance is widespread in nature. Resistance determinants can be found in nearly every environment studied to date, including deep-sea sediment [?], an isolated cave microbiome [?], and 30,000-year-old permafrost [?]. This has led to the recognition that environmental bacterial communities can serve as diverse reservoirs of antimicrobial resistance genes, termed the environmental resistome. There is substantial evidence indicating that, in the past, human pathogens have acquired resistance traits originating in non-pathogenic bacteria that inhabit natural environments (Martinez 2009). It is reasonable to assume that this transfer of environmental resistance factors to human pathogens is ongoing [?]. It is therefore imperative to identify source environments where resistance genes can be selected for and subsequently mobilized into human or animal pathogens.

Wastewater treatment plants (WWTPs) have been demonstrated to contain a large number of antibiotic-resistant bacteria (ARB) and antibiotic resistance genes (ARGs) associated with resistance to all known classes of antibiotic [? ? ? ?]. The treatment of wastewater typically results in substantially reduced concentrations of antibiotics and other pharmaceuticals, in addition to eliminating a significant portion of the resistant bacteria present in untreated wastewater [?]. However, despite the efficacy of modern WWTPs in removing ARB and ARGs from wastewater, some resistance determinants will inevitably persist. In some cases, ARGs have been found in treated effluent at similar or even higher rates than measured in the influent [? ?]. Antibiotic compounds and resistant bacteria that survive wastewater treatment are subsequently released into receiving bodies of water, such as lakes and rivers. Continuous discharge of these contaminants can lead to elevated background levels of resistance [? ? ?], enhancing the likelihood of ARGs being transferred to human commensals or pathogens in the environment.

The localization of ARGs on mobile genetic elements (MGEs), such as transposons and plasmids, enables their movement between bacterial cells of the same or different species. Transfer of resistance factors is likely to increase during exposure to selection factors such as antibiotics and other environmental pollutants [?]. The conditions in WWTPs, including the mixture of organisms from diverse environmental origins and the availability of surfaces and biofilms, can create strong selection pressures for resistance [? ?]. Even those treatments that are effective in removing bacteria from the water can promote the exchange, selection, and dispersal of genes involved in antibiotic resistance [? ?].

Recently, metagenome sequencing of wastewater effluent has been implemented as a useful tool for monitoring the spread of ARGs into natural environments (e.g. [? ?]). Few studies, however, have investigated the environmental resistome throughout a single urban watershed from its headwaters to a major drainage. In this study, we sequenced metagenomes from 72 river samples collected from an urbanized watershed with the goal of assessing the impact of point sources of human waste on the resistance profile of receiving river surface waters, focusing especially on ARGs associated with MGEs.

Data Description

Surface water samples were collected for DNA sequencing and measurements of stream chemistry and physical parameters from 24 sites along three of the rivers comprising the Blue River Watershed: the Blue River and tributaries Indian Creek and Tomahawk Creek. The Blue River watershed was selected based on its high population density, long history of waste overflow from a combined sewage system, and the presence of multiple, high-capacity WWTPs. Sampling site locations were selected based on several factors, including proximity to headwaters and confluences as well as potential sources of pollution such as wastewater treatment plants, hospitals, and drug manufacturing plants.

The surface water samples were analyzed by shotgun metagenomic sequencing, which generated a total of 8.6 billion read pairs. Additional samples were also collected for *E.coli* enumeration and antibiotic susceptibility testing.

Analyses

Detection of ARGs and MGEs in river metagenomes

Antibiotic resistance genes (ARGs) were detected in river water metagenome assemblies using AMRFinder v1.04 [?]. A total of 88 unique ARGs were detected in the watershed, in principle conferring resistance to 12 different classes of antibiotic and an additional four multidrug resistance phenotypes: MLSb, ML, MSb, and LSa. Sulfonamide resistance (26%), followed by aminoglycoside, beta-lactam and macrolide resistance (24%, 16, and 14%, respectively), made up the largest percentage of the watershed resistome (Supplementary Table 1; Additional File 1). The most abundant and commonly occurring ARG was sul1, which was detected in 22 of the 24 sampling sites (Table 1). With the exceptions of sul1 and blaIND, the majority of the highest abundance genes (those composing more than 1% of the total watershed resistome) were found exclusively downstream from potential point sources of human pollution (Supplementary Figure 1). A wide range of mobile genetic elements (MGEs) were also detected in river water metagenome assemblies. A number of these elements were located in close proximity to one or more resistance genes, and we verified many examples of ARGs encoded within complete integrons or transposable elements.

ARGs are more abundant and more diverse downstream from WWTPs

The total abundance of ARGs was found to be significantly higher in river waters sampled immediately downstream from WWTP discharge (Figure 1; ANOVA, p-adjusted = 0.00051 and p-adjusted = 0.0006728, respectively). On average, a 140-fold increase in ARG abundance was observed in samples collected from within 5 km downstream of a WWTP. A 30-fold increase in ARG diversity was also observed in samples collected from downstream surface waters. Of the 88 different ARGs detected in the watershed, 77 of them were detected at downstream sites (averaging 20 per site), while only 15 were detected at upstream sites (averaging 3 per site). ARGs associated with WWTP discharge included those associated with resistance to lincosamide, macrolide, chloramphenicol, fluoroquinolone, polypeptide, trimethoprim, tetracycline, and rifamycin antibiotics as well as with the multidrug-resistant phenotypes MLSb, ML, MSb, and LSa.

ARGs associated with resistance to beta-lactam, aminoglycoside, and sulfonamide antibiotics, in contrast, were common to both upstream and downstream sites. A single sulfonamide resistance gene (sul1) was present in 22 of 24 sampling sites and comprised 87% of total abundance of all sulfonamide resistance genes. Beta-lactamases were also detected with high frequency throughout the watershed, including in 78% of the upstream samples and 75% of the downstream samples. There was no significant difference between upstream and downstream samples in the abundance of sulfonamide resistance genes (FDR = 0.647) or beta-lactam resistance genes (FDR

Table 1. Top 25 resistance genes detected in the watershed.

Resistance gene	Class	Average abundance per rpoB gene	Percent watershed resistome	Sample | Site prevalence
sul1	Sulfonamide	4.48E-03	23.65	68 | 22
ant(3")-II	Aminoglycoside	2.33E-03	12.33	12 | 4
aadA	Aminoglycoside	1.00E-03	5.28	30 | 10
msr	MSb	9.12E-04	4.82	24 | 8
mph(E)	Macrolide	8.02E-04	4.24	24 | 8
blaIND	Beta-Lactam	6.07E-04	3.20	24 | 8
cfxA	Beta-lactam	5.32E-04	2.81	17 | 6
sul2	Sulfonamide	4.51E-04	2.38	38 | 13
mph(G)	Macrolide	4.27E-04	2.25	18 | 6
aph(6)-I	Aminoglycoside	4.13E-4	2.18	20 | 7
mef(A)	Macrolide	4.00E-04	2.11	21 | 7
blaOXA-2	Beta-Lactam	3.55E-04	1.88	24 | 8
blaA	Beta-Lactam	3.51E-04	1.86	26 | 9
tet(C)	Tetracycline	3.42E-04	1.81	24 | 8
tet(Q)	Tetracycline	3.39E-04	1.79	21 | 7
tet(M-W-O-S)	Tetracycline	3.09E-04	1.63	18 | 6
erm(F)	MLS	3.03E-04	1.60	18 | 6
aph(3")-Ib	Aminoglycoside	2.93E-04	1.55	18 | 6
aadA1	Aminoglycoside	2.80E-04	1.48	15 | 5
mef(C)	Macrolide	2.71E-04	1.43	24 | 8
blaOXA-10	Beta-Lactam	2.58E-04	1.36	24 | 8
tet(A-B-C-D)	Tetracycline	2.10E-04	1.11	15 | 5
mef(B)	Macrolide	1.74E-04	0.92	18 | 6
blaOXA	Beta-Lactam	1.65E-04	0.87	18 | 6
blaVEB	Beta-Lactam	1.63E-04	0.86	8 | 3

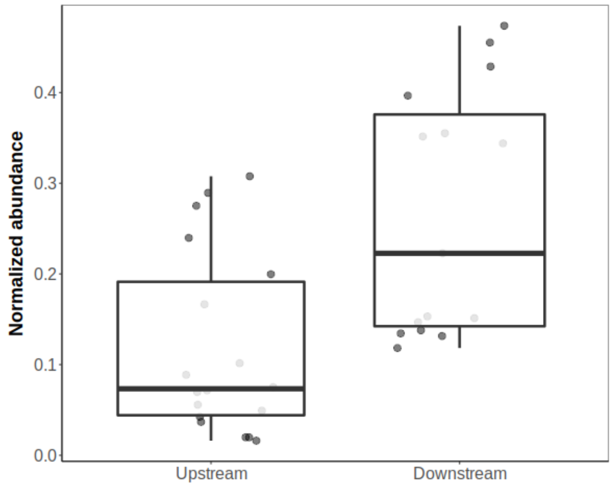

Figure 1. Total abundance of ARGs and MGEs in relation to proximity to WWTP. Normalized abundances of total ARG (top) and MGE (bottom) were square root-transformed for analysis. Samples are grouped based on where they were collected relative to the nearest WWTP (Upstream = surface waters with no impact from a WWTP; Downstream = within 5 km downstream the nearest WWTP). The area between the lower and upper hinge represents the inter-quartile range (IQR), or difference between the first and third quartiles.

= 0.52). Aminoglycoside resistance genes, exclusively encoding agent-modifying enzymes, were detected in 64% of downstream samples and 22% of upstream samples, with a 12-fold enrichment in abundance downstream from WWTP discharge sites (FDR < 1.014e-06).

Proximity to WWTPs was an important factor influencing the abundance of ARGs in river surface waters. Total ARG abundance decreased substantially at sites located more than 5 km downstream from WWTPs compared to sites within 5 km (Supplementary Figure 2; ANOVA, p-adjusted = 0.00058). The log fold-change in ARG diversity was inversely correlated with the downstream distance from WWTP discharge points (Supplementary Figure 3; linear regression, adjusted R^2 = 0.7425, p = 0.0008314). Notably, MGE abundance, while not found to be significantly higher immediately downstream from WWTPs than upstream, also decreased with distance from the WWTPs (Supplementary Figure 4; ANOVA, p-adjusted = 0.0007689).

ARG abundance is correlated with a marker of human fecal pollution

The relationship between total ARG abundance and the abundance of crAssphage was investigated in order to test whether increased abundances of ARGs could be explained by human fecal pollution. crAssphage is a highly abundant bacteriophage in human fecal metagenomes [?] and is rare in feces from non-human animals [?]. The abundances of ARGs and crAssphage were highly correlated with each other in river samples downstream from WWTPs (Figure 2; linear regression, adjusted R^2 = 0.54, p = 5.196e-09). The highest levels of crAssphage were observed immediately downstream from WWTPs, with lower levels detected at more distant sites, following the general trend observed with total ARG abundance.

No correlation was found between crAssphage and ARG abundances in samples collected from sites without an upstream WWTP (linear regression, adjusted R^2 = -0.03, p = 0.73). Nearly all of the upstream sites were located in areas with relatively high population density, and crAssphage sequences were detected in 85% of the upstream samples. Therefore, the lack of correlation between crAssphage and ARG abundances in upstream sites is not due to a complete absence of human fecal pollution but suggests the influence of additional environmental factors that were not measured in this study.

ARGs are associated with MGEs

The potential of ARGs to be transferred between cells was investigated by identifying ARGs located on MGEs such as plasmids, transposons, and integrative conjugative elements (ICEs). The number of unique ARGs encoded on MGEs (mARGs) was signif-

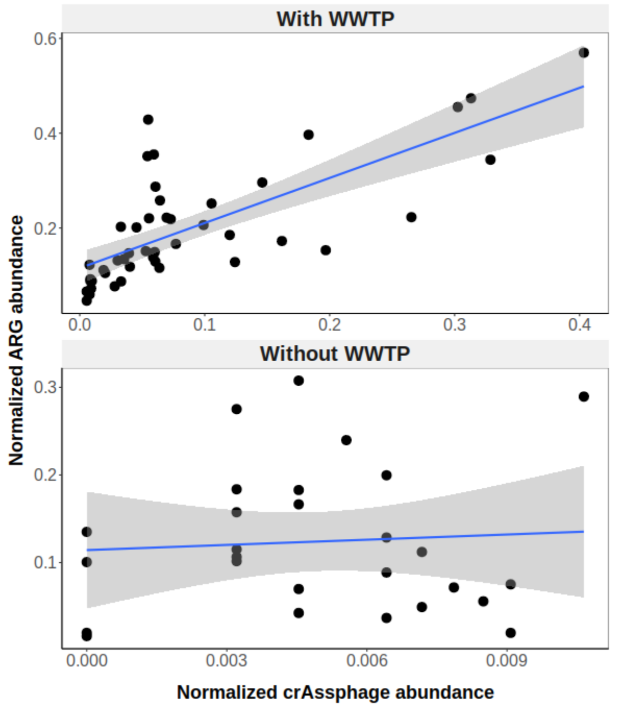

Figure 2. Relationship between ARG and crAssphage abundance. Normalized abundances of total ARG and crAssphage DNA were square root-transformed for analysis. Sites are grouped according to whether one or more WWTP is located upstream the sample site (top) or not (bottom). Smoothing curves based on linear regression (blue line) are shown along with 95% confidence intervals (shaded regions). Note the smaller range of crAssphage abundances in samples without upstream WWTPs.

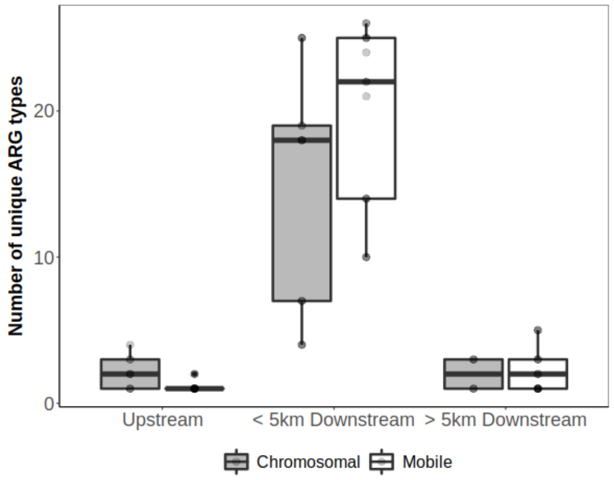

Figure 3. Diversity of ARGs and ARG-encoding plasmids/ICEs in relation to WWTP. Samples are grouped based on where they were collected relative to the nearest WWTP (Upstream = samples are of surface waters with no impact from a WWTP; <5 km downstream = samples were collected within 5 km downstream the nearest WWTP; >5 km downstream = samples were collected from sites greater than 5 km downstream nearest WWTP). The area between the lower and upper hinge represents the inter-quartile range (IQR), or difference between the first and third quartiles.

icantly higher at sites immediately downstream from WWTPs (Figure 3; randomization, $p < 0.001$). The number of mARGs rapidly diminished with increasing distance downstream (randomization, $p < 0.001$). On average, the number of mARGs immediately downstream from WWTPs (averaging 19 per sample) was slightly higher than the number of ARGs assumed to be chromosome-encoded due to lack of evidence to the contrary (cARG; averaging 15 per sample); however, the difference was not found to be significant (ANOVA, $p = 0.09$). Individual mARGs were more often than cARGs to be found in multiple sites downstream from WWTPs, consistent with the ability of mARGs to be shared among multiple bacterial species.

Of the 37 mARGs detected in the watershed, a majority (84%) could be found within 5 km downstream from a wastewater discharge site. Only two mARGs (blaTEM and sul1) were observed in sampling sites upstream from all WWTPs. The sulfonamide resistance gene sul1 was found in two distinct mobile contexts throughout the watershed (Supplementary Table 2), but in upstream sites, it appeared primarily in an integron that also contained the quaternary ammonium compound (QAC) resistance genes qacE and qacG (Figure 4). This integron had closest sequence similarity to integron In78 (100% identity over 60% of the sequence), previously associated with *Pseudomonas aeruginosa* [?].

Downstream sites hosted mobilized genes conferring resistance to many classes of antibiotic, including tetracycline, macrolide, aminoglycoside, fluoroquinolone, and lincosamide antibiotics as well as several likely plasmid/ICE-encoded genes responsible for the macrolide-lincosamide-streptogramin resistance phenotype (Supplementary Table 2). Unlike sul1, the plasmid-encoded sul2 variant was found exclusively downstream from WWTPs and in multiple different mobile contexts, often with a rolling-circle-type transposase. On one such contig with 100% identity to plasmid R485, sul2 was found together with a likely ISCR transposase, a toxin-antitoxin system, multiple conjugation proteins, and an integrase matching those from the Tn916 family of conjugative transposons.

Resistance phenotypes detected by antibiotic susceptibility assays

Escherichia coli colonies were cultivated from river water samples that were collected simultaneously with the samples for metagenomics sequencing. Curiously, no assembled metagenomic sequences were classified as *E. coli*, indicating that our sequencing and assembly approach was not sensitive enough to detect *E. coli* populations of this density.

E. coli colonies were tested for their susceptibility to a variety of antibiotics, including those associated with resistant Enterobacteriaceae pathogens identified in the 2013 CDC Antibiotic Resistance Threat Report as well as additional antibiotics with clinical significance. Antibiotic-resistant *E. coli* were isolated at 7 of the 24 sampling sites (Supplementary Table 3). Ampicillin resistance was the most common phenotype (6% of isolates), followed by amoxicillin-clavulanate, cefazolin, and cefoxitin resistance (3% of isolates each). No isolates were resistant to glycopeptide, trimethoprim, or sulfonamide antibiotics.

Colistin resistance was determined by measuring minimum inhibitory concentrations (MIC) with broth microdilution plates. The most frequently observed colistin MIC was 0.5 ug/mL. Three of the 70 isolates exhibited MICs of 2 ug mL^{-1}; colistin resistance is defined as a MIC >2 ug mL^{-1}. A single isolate exhibited a colistin MIC of 8 ug mL^{-1}. The metagenome of the site where this isolate was isolated (CPA) contains the colistin resistance gene mcr-5. No other genes conferring resistance to colistin were detected in the watershed.

Discussion

Antibiotic resistance genes (ARGs) have been previously detected in the discharge of wastewater treatment plants

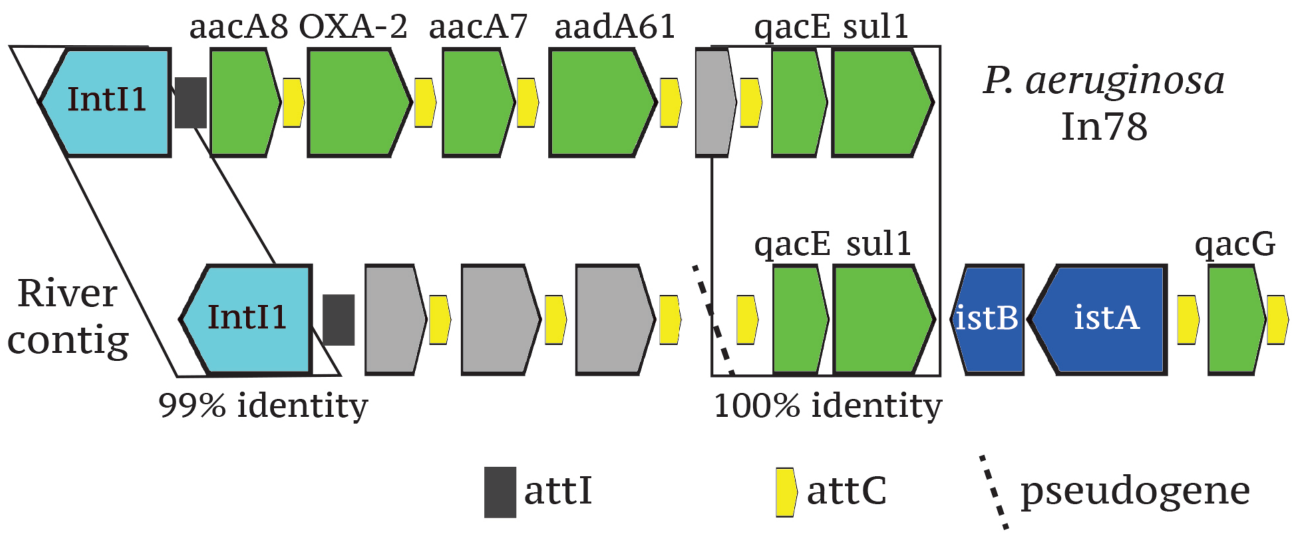

Figure 4. Novel sul1-bearing integron and closest match In78 from *P. aeruginosa*. In addition to the sul1-qacE cassette, the contig associated with the novel integron contained a full-length integron integrase and associated integrase and cassette attachment sites, 3 uncharacterized protein-coding genes, IS21-family transposition genes istA and istB, and a second QAC resistance gene (qacG). The boxed areas of the schema show the regions of alignment between integrons.

(WWTPs) [? ? ? ?], as well as in receiving aquatic environments such as rivers and lakes [? ? ? ? ? ? ?]. Here, we conducted an extensive metagenomic study to investigate the distance decay effect of four wastewater treatment plants on the resistance profile of an urban watershed. River samples collected immediately downstream from WWTPs had significantly higher environmental abundance and diversity of ARGs, and this effect diminished in samples collected >5 km downstream. These results provide additional support for a localized effect of WWTPs on the resistome of receiving aquatic environments.

Our results also show that ARGs associated with WWTPs are likely to be encoded on mobile genetic elements (MGEs) such as plasmids and transposons. Nearly half of the unique ARGs detected downstream of the WWTPs were encoded on an MGE, and all of these ARGs have been previously associated with resistance to clinically relevant drug families. For instance, the erythromycin ribosome methylase (erm) genes have been identified as part of a core WWTP resistome [?], and have consistently been found enriched in biofilms and surface waters downstream from WWTP discharge [? ?]. All of the erm genes detected in our study, including erm(F) and erm(B), were located in assembled sequences identified as plasmids or ICEs.

A number of other resistance determinants were also associated with MGEs. These included the linked aminoglycoside resistance genes strA and strB, which have previously been detected in WWTP effluent [? ?] and other aquatic environments subjected to human pollutants [?]. The strA-strB genes are typically encoded on broad-host-range non-conjugative plasmids as well as conjugative plasmids associated with Tn3-type transposons [?]. Within the Blue River watershed, strA-strB was encoded on a complete Tn3-like transposon with 100% identity to transposon Tn5393d from *Alcaligenes faecalis*. Contigs containing the complete transposon were found at two sites, each downstream from a potential point source of ARG pollution (LDP and UMC), while smaller contigs containing a subset of this region were found downstream from all four WWTPs. The strA-strB genes were not found in any of the upstream samples.

The spectinomycin resistance gene aadA, another member of the core WWTP resistome described in Munck et al. (2015), was also found in samples downstream from all WWTPs and in no upstream samples. In our study, aadA genes frequently co-occurred with other ARGs and with multiple MGEs. For instance, aadA, sul1, qacEdelta, and an IS66 transposase were present on an assembled contig with >99% similarity to the conjugative tetracycline resistance plasmid pFBAOT6.

Plasmid-mediated quinolone resistance, conferred through the pentapeptide repeat protein qnr(S), was also detected immediately downstream from all WWTPs. The qnr(S) gene has consistently been found enriched in WWTP effluent and receiving waters [? ? ?].

In the two largest rivers investigated here, Blue River and Indian Creek, WWTPs contribute on average 15% of base flow [?], and could contribute over 95% under certain conditions [?]. Despite the significant contribution of WWTPs to both streamflow and ARG abundance and diversity, the WWTP-associated ARGs did not persist in surface waters more than 5 km downstream from effluent discharge points. This diminishing effect of elevated ARG abundances in receiving waters with increasing distance from WWTPs has been observed elsewhere [? ? ?]. These results, combined with the strong correlation of total ARG abundance with the abundance of a human gut phage, is consistent with the interpretation that ARGs were released into surface waters with human fecal pollution [?] and then diluted in downstream waters [? ?].

The abundance of ARGs in sites upstream from all WWTPs, in contrast, were independent of human fecal pollution levels. The relatively high abundances of ARGs in these sites suggests the influence of agricultural pollution near the headwaters, but this hypothesis was not tested by the current study. The most abundant ARG in upstream samples was the sul1 sulfonamide resistance gene, which was primarily encoded on a class 1 integron along with two QAC resistance genes. This same mobile element was ubiquitous throughout the entire watershed, spanning multiple streamflows, land use types, and pollution levels, suggesting that it may be maintained in natural microbial communities due to a variety of selection pressures.

Potential implications

Our metagenomic study of an urban watershed has shown that 1) ARGs are widespread in natural aquatic environments, 2) WWTPs introduce a characteristic profile of mobilized ARGs into receiving rivers and streams, 3) Human fecal pollution and

associated ARGs can be effectively diluted by natural microbial populations within several kilometers from discharge points, and 4) the dispersal of specific ARG-encoding MGEs with conserved genomic structures can be traced throughout the watershed.

Characterization of the genomic context of ARGs, such as the novel, ubiquitous integron containing three different antimicrobial resistance genes, was enabled by assembly of the metagenomes, a computationally challenging task that is not routinely performed in such studies. The additional genomic information obtained from metagenomic assembly comes at the cost of reduced sensitivity of detection of sequences that have low abundances and those that are difficult to assemble. Nevertheless, the ability to detect ARGs in new genomic contexts and trace their dispersal among genetic elements, organisms, and environments is a powerful tool for the surveillance of antibiotic resistance in natural environments and potentially for the early detection of emerging resistance genotypes of clinical relevance. Future work should continue to develop and validate metagenomic methods for the quantitative measurement of antimicrobial resistance in natural environments.

Methods

Description of study sites

The Blue River watershed encompasses 450 square kilometers and includes the southern half of the Kansas City metropolitan area below the Missouri River. The majority of water in the Kansas City metropolitan area and in many of the adjacent towns to the south and west drains into the Blue River. The Blue River basin is primarily urban and suburban, with a population density of 794.8 persons/km^2. Six WWTPs are located in the basin, three of which near-continuously discharge treated effluent directly into the Blue River or one of its tributaries while a fourth has occasional wet-weather discharges into the lower Blue River. WWTPs provide the dominant source of streamflow, nutrients, and pharmaceutical compounds to the middle and lower reaches of the Blue River during base flow [?]. Twelve sites on the main stem of the Blue River, ten sites on the tributary Indian Creek, and two sites along Tomahawk Creek were investigated (Figure 5). Accessible locations bracketing WWTP discharges (n=4) were specifically targeted to capture the impact of WWTPs on the stream. Two sites along Tomahawk Creek, which does not contain any WWTPs, were sampled as additional upstream background samples for one WWTP site, which was located downstream from both the effluent discharge point and the confluence between Indian Creek and Tomahawk Creek. Additional samples from sites at various points throughout the watershed were collected as reference samples. Characteristics of the four WWTPs and a summary of the sampling sites are presented in Supplementary Tables 4 and 5, respectively.

Sample collection

A portable peristaltic pump was used to collect surface water samples by either pumping water directly from the stream/river into their respective containers or through sequential in-line filters. At each sampling site, the pump tubing inlet was submerged several centimeters beneath the water surface and held in place through the use of tube weights or anchors. An attempt was made to sample from a location in the stream/river where flow appeared to be greatest or most representative of total flow. Whenever possible pump tubing was positioned upstream to reduce unnecessary exposure to con-

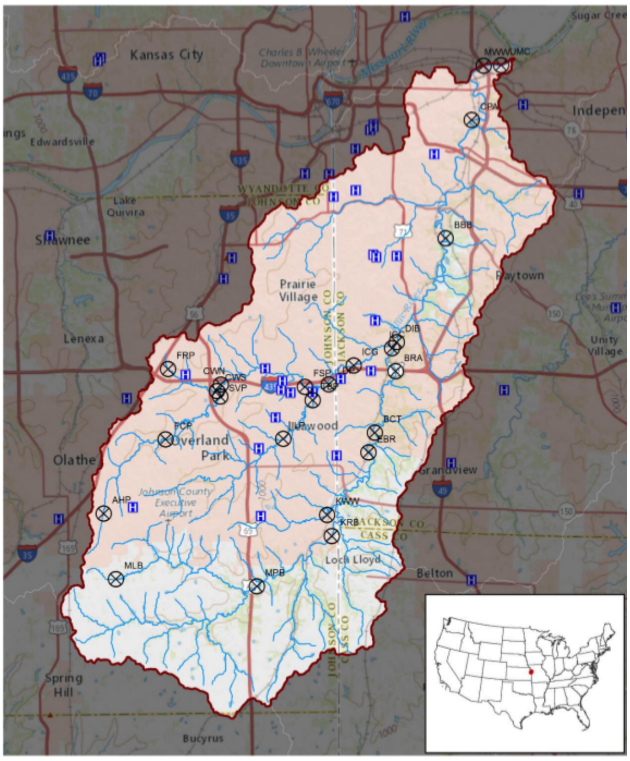

Figure 5. Location of sampling sites within the Blue River watershed. Sampling sites are marked with an X. Locations of hospitals are marked with a blue H.

taminants from sampling equipment and personnel, and caution was taken during the placement of the sampling equipment to avoid streambed disturbance upstream of the collection site. To avoid introducing contamination between sites the pump tubing was rinsed between sampling locations with ultrapure water and 80% ethanol and purged prior to sample collection with site water for at least one minute after placement of the tubing inlet. Physical and chemical parameters of the stream/river water, including dissolved oxygen, specific conductance, total dissolved solids, pH, oxidation-reduction potential, and temperature (Supplementary Table 6), were measured simultaneously with sample collection using a YSI Pro Plus portable multi-parameter water quality meter (YSI Incorporated), and turbidity was measured with a Mi415 turbidity meter (Martini instruments).

For each sample collected for metagenomic sequencing, site water was filtered sequentially through a 5.0 um mixed cellulose filter (5 um pore-size; 47 mm diameter; MF-Millipore; Millipore), followed by a 0.2 um Sterivex cartridge filter (0.22 um pore-size; Sterivex; Millipore), until either clogging of the filters substantially slowed water flow or a total volume of five liters had been filtered. This maximum volume was previously determined to be a reasonable compromise between expediency and thoroughness when sampling river water for metagenomic sequencing, despite considerable variation in sampling site water quality, and was found to be sufficient for this study as well. Depending on the level of suspended particles in the site waters, the volume of water filtered varied from 240 to 5000 mL. Volume filtered was an important factor affecting the amount of DNA that could be extracted from the filters and, consequently, the quality of a metagenomic dataset. A correction for difference in volume filtered was factored into calculations of gene abundance to account for this. Immediately after sampling, remaining fluid was purged from the filter assembly/cartridge and the 5.0 um and 0.2 um filters placed into separate, sterile whirlpool packs and frozen on dry ice before transportation

to the laboratory, where they were stored at -80 °C until processing. Field replicates, in triplicate, were collected at each sampling site and processed individually.

The 5.0 um prefilter was implemented as a screen for filtering plant debris and large eukaryotic cells to increase the coverage depth of genes associated with antibiotic resistance, which are exclusive to bacteria. In addition, the 5.0 um prefilter removed large particles, biofilms, and aggregates, which can unpredictably swamp samples of the free-living, suspended river communities collected by the 0.2 um filters. This smaller size fraction was the focus of the study, as it was considered to be more representative of suspended cells that are dispersing through the watershed rather than being retained in sediments and debris. No sequencing was performed with the 5 µm filters; therefore, genes encoded by large cells or cells associated with aggregates and biofilms are likely to be under-represented in our study.

Samples for *E. coli* enumeration and antibiotic susceptibility testing were collected into sterile, 100 mL clear plastic bottles and then serially diluted on-site in a phosphate buffer solution. Dilutions were filtered through a 0.45 um membrane filter and the filters aseptically removed from their assemblies and placed onto USEPA-approved chromogenic/fluorogenic media for the detection and enumeration of E. coli and total coliforms in water (U.S. Environmental Protection Agency Approved Method 1604: Total Coliforms and Escherichia coli in Water by Membrane Filtration Using a Simultaneous Detection Technique (MI Medium).). Inoculated plates were inverted and set to incubate at 35 °C for 20 to 24 hours. Following incubation, colonies exhibiting phenotypes typical of *E. coli* (blue-green colonies) were counted under ambient light using manufacturer's guidelines. Plates were then sealed with parafilm and stored in coolers with ice for transportation to the lab, where they were refrigerated for later culturing. Field blanks were collected at roughly one-quarter of the sites (chosen at random) and consisted of distilled water. Field blanks were processed in the same manner as the study samples. No growth was observed on all but one of the field blanks, which is suspected of having been contaminated with unsterilized forceps due to the unique pattern of growth.

Antibiotic susceptibility testing

Once in-lab, three colonies per sample that had been identified as *E. coli* on the MI medium were selected for subsequent phenotypic resistance testing. The chosen colonies were plated onto blood agar and incubated at 37 °C. The purity of the cultures was reassessed on chromogenic agar (red-pink colonies) whenever blood agar colonies exhibited atypical morphology. Three of the 74 plates made for susceptibility testing were found through this additional screening measure to be contaminated and removed from testing. Following incubation for approximately 24 hours, sensitivity of the remaining isolates to 16 antibiotics, including ampicillin, amoxicillin, cefazolin, cefepime, ceftriaxone, ceftazidime, ceftazidime with clavulanic acid, cefoxitin, cefotaxime, cefotaxime with clavulanic acid, meropenem, ciprofloxacin, aztreonam, sulfamethoxazole-trimethoprim, gentamicin and tetracycline, was determined through the Kirby-Bauer method with BBL Sensi-Disc Antimicrobial Susceptibility Test Discs (BD). Testing was performed according to CLSI protocols (CLSI, 2016) with the following modification: a BBL Prompt Inoculation System (BD) was used to pick between 3 and 5 colonies from the blood agar for standardized preparation of 0.5 McFarland turbidity level bacterial inocula. This procedure has been shown to have high correlation with conventional disk diffusion techniques, which involves the suspension of colonies in saline followed by dilution to 0.5 McFarland. Within 15 minutes of preparation, the suspension was used to inoculate Mueller-Hinton (MH) agar plates using standard swabbing technique. Between 5-6 antibiotic disks were applied to each of 3 plates per isolate to prevent overlapping zones. Inhibition zones were measured to the nearest millimeter for each of the 16 antibiotic disks by visual inspection of the inoculated plates following overnight incubation at 37 °C, and the zones interpreted through a Zone Diameter Interpretive Chart to determine susceptibility category.

Resistance to colistin was determined through broth microdilution (BMD). Because the large size of the colistin molecule prevents sufficient diffusion through agar medium, BMD is the only procedure recommended by the European Committee on Antimicrobial Susceptibility Testing (EUCAST) for antimicrobial susceptibility testing of colistin [?]. Minimum inhibitory concentration (MIC) for colistin was measured using MIC-strip colistin microdilution plates according to manufacturer's instruction (8 x 12 well plates; Merlin Diagnostics). Briefly, 50 ul of the standardized inoculum was added to 11 mL MH broth. The suspension was gently vortexed and a 100 ul aliquot per well transferred to microdilution strips containing MH broth supplemented with a 2-fold serial dilution of antibiotic. Final concentrations of antibiotic ranged from 0.0625 um/mL to 64 um/mL. Inoculated plates were sealed and incubated at 37 °C. Following incubation for 20-24 hours, MICs were determined through visual inspection of the plates under ambient and supplemented light. MIC was defined as the concentration of drug at which no growth was visible. Isolates were retested whenever a skipped well was observed during inspection of the BMD strips or for additional confirmation for *E. coli* isolates with MIC exceeding 2 ug/mL.

Metagenomic sequencing

Extraction of DNA from cells retained on the 0.2 um Sterivex filters was performed according to an established lab protocol [?], briefly described here. For the complete protocol, see "Availability of supporting data and materials". To help mitigate any variation that might be introduced during laboratory handling, DNA extraction was performed on the Sterivex filters in random order, with extraction order resolved beforehand. Filter cartridges were injected with extraction buffer, incubated at 65 °C for 30 minutes, and then the fluid was extracted from the filter cartridge and distributed into 0.1 mm glass bead tubes, which were shaken for 40 seconds in a MiniBeadBeater-16 607 (3450 oscillations/min; BioSpec). Bead tubes were centrifuged, and the supernatant was purified by extraction with phenol/chloroform/isoamyl alcohol (25:24:1) and chloroform/isoamyl alcohol (24:1). DNA was preciptated overnight at -20 °C in sodium-acetate/ethanol, and the pellets were resuspended in low-EDTA TE. DNA was not extracted from the 5 um prefilters, which remain in frozen storage for potential future analyses.

Whenever possible, 80 ng of purified DNA was used in the construction of metagenomic libraries. Total DNA extracted from the filters was quantified on a Qubit fluorometer (ThermoFisher). The quality of the sample DNA was also assessed before library construction with a Nanodrop spectrophotometer (ThermoFisher), and further purification conducted as needed based on Nanodrop 260/230 and 260/280 ratios using magnetic bead-based cleanup following a protocol adapted from Rohland & Reich (2012) [?]. The 260/230 curve was particularly predictive of library preparation success for this study, with a significant correlation found between Nanodrop 260/230 ratios and the final concentration of DNA in the prepared libraries (linear regression, adjusted R^2 = 0.283, p = 5.267e-11). Purified DNA

was fragmented by sonication with a Q800R sonicator (QSonica) at 4 °C, 25% amplitude, with a 10-second pulse for 60 seconds. These settings were selected to achieve a high molecular weight band on an agarose gel around the 500-700 base pair (bp) range. Magnetic beads were then used to select molecules from the fragmented DNA with a target size range of 500 to 700 bp. The size and quality of the fragments were verified with gel electrophoresis on a random subset of samples. Libraries were prepared from the size-selected DNA for metagenomic sequencing using the NEBnext Ultra DNA library prep kit for Illumina according to manufacturer instructions. A final bead cleanup was performed on the prepared DNA to remove excess kit reagents that could interfere with sequencing, and stored at -20 °C until transportation to the sequencing center. Each of the triplicate samples per site was processed separately, resulting in a total of 72 metagenomic libraries for sequencing.

Quality control and sequencing of the metagenomic libraries was conducted at the University of Utah High-Throughput Genomics Core Facility. Libraries were evaluated for quality on a Bioanalyzer DNA 1000 chip (Agilent Technologies), and then paired-end sequencing (2 x 125 bp) was performed on an Illumina HiSeq2500 platform with HiSeq v4 chemistry. Libraries were multiplexed and pooled 4 per lane for a total of 19 lanes of Illumina sequencing, yielding over one trillion bp of data (Supplementary Table 7). Demultiplexing and conversion of the raw sequencing base-call data were performed through the CASAVA v1.8 pipeline.

Sequence assembly

Quality control of the sequencing reads was performed with BBDuk v37.10 and consisted of contaminant removal and quality-based trimming. First, contaminants in the form of library adapter sequence were trimmed/filtered from the reads. An adapter was considered to be present whenever an adapter reference sequence shared a 23-mer with a read, or an 8-mer if located at the extreme 3' end. A Hamming distance of two was allowed between matching k-mers. Adapter sequences were also detected from completely overlapping read pairs. Additional contaminants in the form of PhiX DNA were identified and removed if 90% of a read's length was covered by the PhiX174 reference genome. PhiX DNA was used as a spike-in control during sequencing at concentrations representing approximately 0.5% of reads generated per lane of flow cell. Reads were compared to the reference genome using k-mer matching with a k-mer size of 31. Matching 31-mers were allowed to differ by a Hamming distance of one.

Reads were further trimmed based on quality using the Phred algorithm in BBDuk and discarded following trimming whenever the final length of a read was less than 52 bp. In a study using publicly available Illumina paired-end transcriptomes, it was found that overly stringent trimming resulted in worse assemblies according to the majority of metrics measured, particularly for low-coverage datasets [?]. The adverse effects of trimming were reduced, however, as coverage was increased. Based on these findings, a gentle trimming strategy was recommended (Phred quality score between 2 and 5) except under specific scenarios when more aggressive trimming is warranted, such as in the case of exceptionally high sequencing depth. We tested the applicability of these guidelines to metagenomic assemblies with a mock microbial community [?]. In nearly all cases, aggressive trimming reduced the number of mismatches and indels between the assembly and mapped short reads (Supplementary Figure 5), but there was an increasingly detrimental impact on measures of assembly performance as quality score threshold was increased, which was especially evident in the lower coverage libraries. Therefore, a low quality threshold was chosen for quality-based trimming (Phred quality score of 5), as the metagenomes were determined by Nonpareil v3.3.1 [?] to not be fully saturated with reads.

Records with both reads in a pair passing quality control were co-assembled by sampling site using Megahit v1.1.1 (Li et al. 2016) with parameters kstart=27, kend=127, and kstep=20, and taxonomic bins reconstructed from the resultant assemblies using PhyloPythiaS+ [?]. The performance of the assembler on the datasets was evaluated based on read mapping rates and statistics provided by MetaQuast v4.6.1 [?]. Megahit was selected for assembly because of its reliable performance on datasets of highly complex microbial communities [? ?]. Megahit is also known to recover larger portions of strain variants than other, comparable short-read assemblers [?].

Identification and quantification of ARGs and MGEs

Contiguous sequences (or contigs) generated during assembly with length less than 200 bp were discarded prior to functional annotation. Putative coding DNA sequences were predicted from the remaining contigs and then translated into protein sequences using Prodigal v2.6.3 [?] in meta mode. Broad functional characterization of the gene predictions was performed through similarity searches against the Kyoto Encyclopedia of Genes and Genomes (KEGG, release 83.2) [?] using the BLASTP subcommand of Diamond v0.9.14 [?] with the more-sensitive flag, a maximum expect value of e-5, and parameters unal=0, max-target-seqs=1, strand=both. Homologs of known antibiotic resistance determinants were identified from the predicted protein sequences using AMRFinder v1.04 with default settings [?]. ARGs detected through AMRFinder with family type equal to "equivalog" or "subfamily" were excluded to ensure that only the highest confidence results were reported (see the AMRFinder documentation for a more thorough description of how ARGs are detected by AMRFinder).

The program hmmsearch (HMMER v3.1b) was used to detect remote homologs of mobile genetic element (MGE) markers by searching a custom database of profile HMMs against the predicted protein sequences using manually curated gathering scores to determine which hits to report. The database was built by supplementing a set of custom profiles with select profiles taken from Pfam v32.0 [?]. Markers selected for inclusion in the database were chosen for their involvement in the transposition of DNA (i.e., integrases and transposases), and represent integrons as well as type I and II transposable elements. To create the custom profiles, groups of related proteins belonging to the same marker family were downloaded from the NCBI RefSeq database of protein sequences. Duplicate sequences were clustered with CD-HIT, and very divergent (less than 20% identity) and very similar sequences (greater than 80% identity) were removed prior to initial multiple sequence alignment (MSA) with MAFFT [?]. Subsequent steps, including filtering, final alignment, and trimming, were performed with T-Coffee [?]. First, sequence CORE (sCORE) scores were generated, and the distribution of scores analyzed to identify outliers. Sequences with poor sCORE were discarded and the 40 most informative sequences extracted. The most informative sequences were defined as the sequences diverging the most from each other and constituted the final set used in the construction of the seed MSA. Residues from the seed MSA with low transitive consistency score [?] were removed, and the resulting curated alignments used to construct the profile HMMs. Model-specific cutoffs were assigned to the profiles as in Punta et al. (2012) [?].

Coverage of the identified features was estimated by individually mapping the quality-controlled short reads to their

relevant assembly using Bowtie2 v2.3.2 [?] with the very-sensitive flag and insert-size min and max parameters provided. These parameters were estimated from the insert-size mean and standard deviation calculated with BBmap v37.10. crAssphage coverage was calculated in an identical manner by mapping short reads to the crAssphage genome (GeneBank: NC_024711.1). To prevent artificial replicates from inflating coverage estimates, replicates in the mapped reads were first identified and removed using the MarkDuplicates functionality of Picard Tools v2.17.8. After duplicate filtering, mapped read best matches were sorted by name using Samtools v1.3 [?] and then used for estimating environmental abundance of the annotated sequences with count_features v1.3.0, part of the seq-annot open-source software package developed for the study.

For estimating feature abundances, read counts - the number of fragments thought to have originated from a given genomic region - were first transformed to fragment proportions (FP), a variant of transcripts per million (TPM) [?] without the application of a scaling factor. Each fragment used in the determination of the read counts was represented by a pair of aligned reads. The count of a feature was incremented whenever its coordinates fell within the interval of an alignment, defined as the region between two ends of a successfully mapped read pair, and an alignment interval overlapping multiple features was considered as separate evidence for the presence of each feature falling within its coordinates. Classes from the HTSeq python library v0.9.1 [?] were incorporated into count_features for storing alignment and feature coordinates.

FP is a measure of the proportion of fragments in the underlying population produced from a given genomic region. Raw counts were replaced with nucleotide fractions by dividing read counts by effective length of the feature. FP further varies from TPM in that the effective length of a feature is equal to its actual length. In metagenomes, any given position of a feature is capable of producing fragments of any length, so consideration of fragment start position is unnecessary in the determination of the counts per bp rate. The nucleotide fraction of each feature was divided by the sum of all counts per bp rates to give the length-adjusted proportion of feature *i* out of *n* total features predicted from sample *k*:

$$FP_{i,k} = \frac{\frac{counts_{i,k}}{length_i(bp)}}{\sum_i^{n_k} \frac{counts_{j,k}}{length_j(bp)}} \tag{1}$$

ARG abundances were normalized to the abundance of the rpoB gene by dividing the FP of each ARG to the FP of rpoB in that metagenomic library.

Detection of mobile ARGs

An assembly reconciliation program was used to merge contigs encoding one or more resistance genes prior to the detection of transposable ARGs. ARG-bearing contigs from across all sites were first combined and clustered at 100% identity. The dereplicated contigs were then merged using Mix v1.0 (Soueidan et al. 2013) with parameters C=100 and A=200. Merged contigs were manually inspected for mis-assembly by mapping the dereplicated contigs onto the merged assembly with BLASTN (BLAST+ v2.7.1) [?]. Contigs that failed to map were then appended to the merged assembly to create the final set of unique ARG-bearing contigs. Components of MGEs were predicted from these contigs as described above with additional annotation in the form of repeat units from Repseek v6.6 [?] and attC attachment sites using cmsearch (INFERNAL v1.1.2) [?] with the covariance model from Cury et al. (2016) [?]. Transposable ARGs were defined as those encoded by a complete intracellular MGE, including autonomous transposons, composite transposons, insertion sequence common regions (ISCRs), and integrons. To be considered complete, an MGE was required to contain its relevant recombination module and be bounded by appropriate attachment/excision sites when applicable. For instance, integron-encoded ARGs were required to co-occur on the same contig as both an attC recombination site as well as an integron integrase.

Transferable ARGs were detected by searching the identified ARGs against a database of plasmid and integrative and conjugative element (ICE) protein sequences using BLASTP with a reporting threshold of greater than 99% sequence similarity. The database was composed of sequences from the ACLAME v0.4 [?] and ICEberg v2.0 [?] databases and supplemented with additional plasmid sequences downloaded from NCBI RefSeq. For each site, contigs were re-mapped to the set of unique ARG-bearing contigs and the results, which included ARG-encoding plasmids/ICEs as well as transposable ARGs, were matched to the ARG abundance table for each metagenome. The proportion of mobile ARG per metagenome was calculated as the abundance of transferable or transposable ARGs over the total abundance of ARGs.

Statistical analyses

All statistical analyses were performed in the R programming language v3.5.1 (R Development Core Team 2008) with the aid of reshape2 v1.4.3 [?] and several graphing and statistical libraries. Figures were generated with the data visualization library ggplot2 v3.1.0 [?], using color palettes from the dichromat package v2.0.0 [?]. Resistome percentages were calculated as the fraction of total normalized abundance for a given ARG or ARG category out of the total normalized abundance for all ARGs detected within the watershed samples. ARG diversity was calculated as the number of different ARG types detected at a site or within the watershed, where a given ARG type represents a unique entry in the AMRFinder database at the allele or exception level.

Linear regressions were performed with the modeling function lm (core stats package), and curves drawn using the geom_smooth layer of ggplot2 with linear model (lm) set as the smoothing method. Normalized ARG abundance was modeled as a linear function of the normalized abundance of crAssphage DNA. To determine if the relationship was dependent on input from known sources of human waste, samples were grouped based on whether they were collected from a site downstream from one or more WWTPs or from a site with no upstream WWTP. A linear model of ARG and crAssphage abundances was compared to one that included an additional interaction term separating samples based on whether they were taken from sites located downstream from WWTPs or from sites upstream from all WWTPs. The model that included the interaction term was found to better fit the data (ANOVA, $p = 0.001927$), so the relationship between ARG and crAssphage abundances within sample groups was further tested individually. Normalized abundances were log-transformed prior to model fitting.

Geographic distance served as a proxy for river distance when assigning sites to one of three groups representing varying distances from WWTPs. Normalized coverage totals were log-transformed for between-group comparisons, and differences in total abundance assessed with one-way analysis of variance (ANOVA) followed by Tukey's posthoc test for pairwise comparisons of group means. When abundance data were

further divided by location of the resistance gene (chromosomal versus mobilized), the log-transformed data failed to meet test assumptions of normally distributed data with equal variance; in this case, significance was instead assessed through a non-parametric randomization procedure involving randomly reassigned sample labels. P-values were calculated by comparing observed F values with distributions generated from ten thousand permutations of the data. The level of statistical significance was set at 0.001 for all statistical tests performed.

Only samples collected from sites located upstream and within 5 km downstream from a WWTP were considered in the analysis of differential abundance (n=18 and n=15 per group, respectively). The change in abundance of ARG totals at downstream WWTP sites was calculated as the log2 of the ratio between downstream and upstream abundance, and statistical significance of differences in abundance between groups assessed using edgeR v3.24.3 [?] on unmodified read counts. Gene categories with False Discovery Rate (FDR) less than 0.05 were considered to be significantly more abundant in one group over the other.

Availability of source code and requirements

- Project name: seq-annot
- Project home page: e.g. https://github.com/Brazelton-Lab/seq-annot
- Operating system(s): Linux
- Programming language: Python
- Other requirements: Python 3.4 or higher
- License: GPLv3
- biotoolsID: biotools:seq-annot
- RRID: SCR_018731
- Any restrictions to use by non-academics: none

Availability of supporting data and materials

The raw metagenomic sequence data sets and the metagenome assemblies supporting the results reported here are available at the NCBI Sequence Read Archive and NCBI WGS, respectively, under BioProject accession PRJNA562643. The protocols used in the extraction of sample DNA can be found at https://baas-becking.biology.utah.edu/data/category/18-protocols. The custom software and scripts used in data processing are available from https://github.com/Brazelton-Lab. The R code used in data analysis is also available at the development GitHub page https://github.com/Brazelton-Lab/Thornton_2019.

Additional files

Additional File 1: Supplementary tables and figures.

Supplementary Table 1. Percent total watershed resistome by drug class.

Supplementary Table 2. ARG-bearing contigs matching known mobile genetic elements.

Supplementary Table 3. Summary of the antibiotic susceptibility testing results.

Supplementary Table 4. Characteristics of the four WWTPs associated with the study.

Supplementary Table 5. Description of sites sampled within the Blue River Watershed.

Supplementary Table 6. Physical and chemical parameters of sample site surface waters.

Supplementary Table 7. Sequencing and assembly statistics.

Supplementary Figure 1. Total normalized ARG abundance by sampling site.

Supplementary Figure 2. Total ARG abundance at varying distances from WWTP.

Supplementary Figure 3. ARG richness with increasing distance downstream a WWTP.

Supplementary Figure 4. Total MGE abundance at varying distances from WWTP.

Supplementary Figure 5. Association between assembly quality and quality score threshold at varying sequencing depths.

Additional File 2: Results of the statistical tests using an alternative normalization measure.

Figure A.S1. Total normalized ARG abundance by sampling site.

Figure A.1. Total abundance of ARGs in relation to proximity to WWTP

Figure A.S1. Total ARG abundance at varying distances from WWTP.

Figure A.2. Relationship between ARG and crAssphage abundance.

Declarations

List of abbreviations

ANOVA: Analysis of Variance; ARB: Antibiotic Resistant Bacteria; ARG: Antibiotic Resistance Gene; AS: Activated Sludge; BMD: Broth Microdilution; BNR: Biological Nutrient Removal; bp: Base Pairs; CARD: Comprehensive Antibiotic Resistance Database; cARG: Chromosomal Antibiotic Resistance Gene; crAssphage: cross-assembly phage; EUCAST: European Committee on Antimicrobial Susceptibility Testing; FDR: False Discovery Rate; FP: Fragment Proportions; HGT: Horizontal Gene Transfer; HMM: Hidden Markov Model; ICE: Integrative and Conjugative Element; ISCR: Insertion Sequence Common Region; KEGG: Kyoto Encyclopedia of Genes and Genomes; LSa: Lincosamide-Steptogramin a mARG: Mobile Antibiotic Resistance Gene; MGD: Million Gallons per Day; MGE: Mobile Genetic Element; MH: Mueller-Hinton; MIC: Minimum Inhibitory Concentration; ML: Macrolide-Lincosamide; MLSb: Macrolide-Lincosamide-Streptogramin B; MSb: Macrolide-Streptogramin B; MSA: Multiple Sequence Alignment; QAC: Quaternary Ammonium Compound; PE: Person Equivalents; TF: Trickle Filter; TF/GC: Trickle Filter with Gravity Clarifiers; TPM: Transcripts per Million; WWTP: Wastewater Treatment Plant.

Ethical Approval

Not applicable.

Consent for publication

Not applicable.

Competing Interests

The authors declare that they have no competing interests.

Funding

This work was supported by contract #200-2016-91949 to PI VanDerslice from the US Centers for Disease Control and Prevention under Broad Agency Announcement 2016-N-17812. The funding body had no role in the design or execution of the study, nor in writing the manuscript.

Author's Contributions

W.J.B. conceptualized the project, designed the experiments, and wrote the manuscript. C.N.T. assisted in the experimental design, performed the experiments, analyzed the data, developed the software, and wrote the manuscript. J.A.V. conceptualized the project and designed the experiments. W.D.T. conceptualized the project and designed and performed the experiments. All authors assisted in data collection and edited the manuscript.

Acknowledgements

We would like to thank Bahvneet Singh, Alex Hyer, and Cody Dangerfield for their assistance in sample collection. In addition, Sharzad Motamedi, Julia McGonigle, and Lizethe Pendleton provided invaluable advice during sample processing and preparation for sequencing.

Manuscript

Click here to access/download;Manuscript;gigascience_2020_manuscript_r

GigaScience, 2017, 1–13

doi: xx.xxxx/xxxx
Manuscript in Preparation
Paper

PAPER

Localized effect of treated wastewater effluent on the resistome of an urban watershed

Christopher N. Thornton[1,*], Windy D. Tanner[2], James A. VanDerslice[2] and William J. Brazelton[1,*]

[1]School of Biological Sciences, University of Utah, Salt Lake City, UT, USA and [2]Department of Family and Preventive Medicine, University of Utah, Salt Lake City, UT, USA

*christopher.thornton@utah.edu; william.brazelton@utah.edu

Abstract

Background
Wastewater treatment is an essential tool for maintaining water quality in urban environments. While the treatment of wastewater can remove most bacterial cells, some will inevitably survive treatment to be released into natural environments. Previous studies have investigated antibiotic resistance within wastewater treatment plants, but few studies have explored how a river's complete set of antibiotic resistance genes (the 'resistome') is affected by the release of treated effluent into surface waters.

Results
Here we used high-throughput, deep metagenomic sequencing to investigate the impact of treated wastewater effluent on the resistome of an urban river and the downstream distribution of effluent-associated antibiotic resistance genes and mobile genetic elements. Treated effluent release was found to be associated with increased abundance and diversity of antibiotic resistance genes and mobile genetic elements. The impact of wastewater discharge on the river's resistome diminished with increasing distance from effluent discharge points. The resistome at river locations that were not immediately downstream from any wastewater discharge points were dominated by a single integron carrying genes associated with resistance to sulfonamides and quaternary ammonium compounds.

Conclusions
Our study documents variations in the resistome of an urban watershed from headwaters to a major confluence in an urban center. Greater abundances and diversity of antibiotic resistance genes are associated with human fecal contamination in river surface water, but the fecal contamination effect appears to be localized, with little measurable impact in downstream waters. The diverse composition of antibiotic resistance genes throughout the watershed suggests the influence of multiple environmental and biological factors.

Key words: antibiotic resistance, wastewater, metagenome, watershed, pollution

Background

The growing public health crisis caused by the emergence and spread of antibiotic resistance is now recognized as a global problem with a complex interplay of environmental, biological, and clinical factors. Research on antimicrobial resistance has historically been focused on human pathogens, with hospitals and other clinical settings thought to be the primary source for the dissemination and evolution of antibiotic resistance. However, in part due to increased reporting of community-acquired, antibiotic-resistant infections [1, 2], researchers have started to broaden their focus to include the role of natural environments as possible reservoirs of antimicrobial resistance and as settings for the evolution of new resistance

Compiled on: September 16, 2020.
Draft manuscript prepared by the author.

determinants.

Antimicrobial resistance is widespread in nature. Resistance determinants can be found in nearly every environment studied to date, including deep-sea sediment [3], an isolated cave microbiome [4], and 30,000-year-old permafrost [5]. This has led to the recognition that environmental bacterial communities can serve as diverse reservoirs of antimicrobial resistance genes, termed the environmental resistome. There is substantial evidence indicating that, in the past, human pathogens have acquired resistance traits originating in non-pathogenic bacteria that inhabit natural environments (Martinez 2009). It is reasonable to assume that this transfer of environmental resistance factors to human pathogens is ongoing [6]. It is therefore imperative to identify source environments where resistance genes can be selected for and subsequently mobilized into human or animal pathogens.

Wastewater treatment plants (WWTPs) have been demonstrated to contain a large number of antibiotic-resistant bacteria (ARB) and antibiotic resistance genes (ARGs) associated with resistance to all known classes of antibiotic [7, 8, 9, 10]. The treatment of wastewater typically results in substantially reduced concentrations of antibiotics and other pharmaceuticals, in addition to eliminating a significant portion of the resistant bacteria present in untreated wastewater [10]. However, despite the efficacy of modern WWTPs in removing ARB and ARGs from wastewater, some resistance determinants will inevitably persist. In some cases, ARGs have been found in treated effluent at similar or even higher rates than measured in the influent [11, 8]. Antibiotic compounds and resistant bacteria that survive wastewater treatment are subsequently released into receiving bodies of water, such as lakes and rivers. Continuous discharge of these contaminants can lead to elevated background levels of resistance [12, 13, 14], enhancing the likelihood of ARGs being transferred to human commensals or pathogens in the environment.

The localization of ARGs on mobile genetic elements (MGEs), such as transposons and plasmids, enables their movement between bacterial cells of the same or different species. Transfer of resistance factors is likely to increase during exposure to selection factors such as antibiotics and other environmental pollutants [15]. The conditions in WWTPs, including the mixture of organisms from diverse environmental origins and the availability of surfaces and biofilms, can create strong selection pressures for resistance [16, 7]. Even those treatments that are effective in removing bacteria from the water can promote the exchange, selection, and dispersal of genes involved in antibiotic resistance [17, 18].

Recently, metagenome sequencing of wastewater effluent has been implemented as a useful tool for monitoring the spread of ARGs into natural environments (e.g. [19, 20]). Few studies, however, have investigated the environmental resistome throughout a single urban watershed from its headwaters to a major drainage. In this study, we sequenced metagenomes from 72 river samples collected from an urbanized watershed with the goal of assessing the impact of point sources of human waste on the resistance profile of receiving river surface waters, focusing especially on ARGs associated with MGEs.

Data Description

Surface water samples were collected for DNA sequencing and measurements of stream chemistry and physical parameters from 24 sites along three of the rivers comprising the Blue River Watershed: the Blue River and tributaries Indian Creek and Tomahawk Creek. The Blue River watershed was selected based on its high population density, long history of waste overflow from a combined sewage system, and the presence of multiple, high-capacity WWTPs. Sampling site locations were selected based on several factors, including proximity to headwaters and confluences as well as potential sources of pollution such as wastewater treatment plants, hospitals, and drug manufacturing plants.

The surface water samples were analyzed by shotgun metagenomic sequencing, which generated a total of 8.6 billion read pairs. Additional samples were also collected for *E.coli* enumeration and antibiotic susceptibility testing.

Analyses

Detection of ARGs and MGEs in river metagenomes

Antibiotic resistance genes (ARGs) were detected in river water metagenome assemblies using AMRFinder v1.04 [21]. A total of 88 unique ARGs were detected in the watershed, in principle conferring resistance to 12 different classes of antibiotic and an additional four multidrug resistance phenotypes: MLSb, ML, MSb, and LSa. Sulfonamide resistance (26%), followed by aminoglycoside, beta-lactam and macrolide resistance (24%, 16, and 14%, respectively), made up the largest percentage of the watershed resistome (Supplementary Table 1; Additional File 1). The most abundant and commonly occurring ARG was sul1, which was detected in 22 of the 24 sampling sites (Table 1). With the exceptions of sul1 and blaIND, the majority of the highest abundance genes (those composing more than 1% of the total watershed resistome) were found exclusively downstream from potential point sources of human pollution (Supplementary Figure 1). A wide range of mobile genetic elements (MGEs) were also detected in river water metagenome assemblies. A number of these elements were located in close proximity to one or more resistance genes, and we verified many examples of ARGs encoded within complete integrons or transposable elements.

ARGs are more abundant and more diverse downstream from WWTPs

The total abundance of ARGs was found to be significantly higher in river waters sampled immediately downstream from WWTP discharge (Figure 1; ANOVA, p-adjusted = 0.00051 and p-adjusted = 0.0006728, respectively). On average, a 140-fold increase in ARG abundance was observed in samples collected from within 5 km downstream of a WWTP. A 30-fold increase in ARG diversity was also observed in samples collected from downstream surface waters. Of the 88 different ARGs detected in the watershed, 77 of them were detected at downstream sites (averaging 20 per site), while only 15 were detected at upstream sites (averaging 3 per site). ARGs associated with WWTP discharge included those associated with resistance to lincosamide, macrolide, chloramphenicol, fluoroquinolone, polypeptide, trimethoprim, tetracycline, and rifamycin antibiotics as well as with the multidrug-resistant phenotypes MLSb, ML, MSb, and LSa.

ARGs associated with resistance to beta-lactam, aminoglycoside, and sulfonamide antibiotics, in contrast, were common to both upstream and downstream sites. A single sulfonamide resistance gene (sul1) was present in 22 of 24 sampling sites and comprised 87% of total abundance of all sulfonamide resistance genes. Beta-lactamases were also detected with high frequency throughout the watershed, including in 78% of the upstream samples and 75% of the downstream samples. There was no significant difference between upstream and downstream samples in the abundance of sulfonamide resistance genes (FDR = 0.647) or beta-lactam resistance genes (FDR

Table 1. Top 25 resistance genes detected in the watershed.

Resistance gene	Class	Average abundance per rpoB gene	Percent watershed resistome	Sample | Site prevalence
sul1	Sulfonamide	4.48E-03	23.65	68 | 22
ant(3")-II	Aminoglycoside	2.33E-03	12.33	12 | 4
aadA	Aminoglycoside	1.00E-03	5.28	30 | 10
msr	MSb	9.12E-04	4.82	24 | 8
mph(E)	Macrolide	8.02E-04	4.24	24 | 8
blaIND	Beta-Lactam	6.07E-04	3.20	24 | 8
cfxA	Beta-lactam	5.32E-04	2.81	17 | 6
sul2	Sulfonamide	4.51E-04	2.38	38 | 13
mph(G)	Macrolide	4.27E-04	2.25	18 | 6
aph(6)-I	Aminoglycoside	4.13E-4	2.18	20 | 7
mef(A)	Macrolide	4.00E-04	2.11	21 | 7
blaOXA-2	Beta-Lactam	3.55E-04	1.88	24 | 8
blaA	Beta-Lactam	3.51E-04	1.86	26 | 9
tet(C)	Tetracycline	3.42E-04	1.81	24 | 8
tet(Q)	Tetracycline	3.39E-04	1.79	21 | 7
tet(M-W-O-S)	Tetracycline	3.09E-04	1.63	18 | 6
erm(F)	MLS	3.03E-04	1.60	18 | 6
aph(3")-Ib	Aminoglycoside	2.93E-04	1.55	18 | 6
aadA1	Aminoglycoside	2.80E-04	1.48	15 | 5
mef(C)	Macrolide	2.71E-04	1.43	24 | 8
blaOXA-10	Beta-Lactam	2.58E-04	1.36	24 | 8
tet(A-B-C-D)	Tetracycline	2.10E-04	1.11	15 | 5
mef(B)	Macrolide	1.74E-04	0.92	18 | 6
blaOXA	Beta-Lactam	1.65E-04	0.87	18 | 6
blaVEB	Beta-Lactam	1.63E-04	0.86	8 | 3

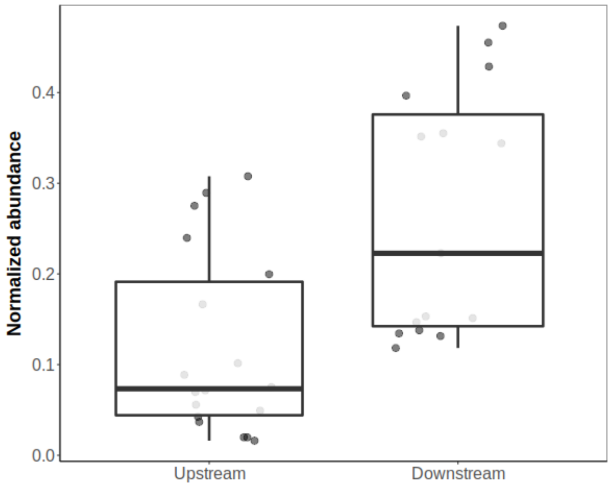

Figure 1. Total abundance of ARGs and MGEs in relation to proximity to WWTP. Normalized abundances of total ARG (top) and MGE (bottom) were square root-transformed for analysis. Samples are grouped based on where they were collected relative to the nearest WWTP (Upstream = surface waters with no impact from a WWTP; Downstream = within 5 km downstream the nearest WWTP). The area between the lower and upper hinge represents the inter-quartile range (IQR), or difference between the first and third quartiles.

= 0.52). Aminoglycoside resistance genes, exclusively encoding agent-modifying enzymes, were detected in 64% of downstream samples and 22% of upstream samples, with a 12-fold enrichment in abundance downstream from WWTP discharge sites (FDR < 1.014e-06).

Proximity to WWTPs was an important factor influencing the abundance of ARGs in river surface waters. Total ARG abundance decreased substantially at sites located more than 5 km downstream from WWTPs compared to sites within 5 km (Supplementary Figure 2; ANOVA, p-adjusted = 0.00058). The log fold-change in ARG diversity was inversely correlated with the downstream distance from WWTP discharge points (Supplementary Figure 3; linear regression, adjusted R^2 = 0.7425, p = 0.0008314). Notably, MGE abundance, while not found to be significantly higher immediately downstream from WWTPs than upstream, also decreased with distance from the WWTPs (Supplementary Figure 4; ANOVA, p-adjusted = 0.0007689).

ARG abundance is correlated with a marker of human fecal pollution

The relationship between total ARG abundance and the abundance of crAssphage was investigated in order to test whether increased abundances of ARGs could be explained by human fecal pollution. crAssphage is a highly abundant bacteriophage in human fecal metagenomes [22] and is rare in feces from non-human animals [23]. The abundances of ARGs and crAssphage were highly correlated with each other in river samples downstream from WWTPs (Figure 2; linear regression, adjusted R^2 = 0.54, p = 5.196e-09). The highest levels of crAssphage were observed immediately downstream from WWTPs, with lower levels detected at more distant sites, following the general trend observed with total ARG abundance.

No correlation was found between crAssphage and ARG abundances in samples collected from sites without an upstream WWTP (linear regression, adjusted R^2 = -0.03, p = 0.73). Nearly all of the upstream sites were located in areas with relatively high population density, and crAssphage sequences were detected in 85% of the upstream samples. Therefore, the lack of correlation between crAssphage and ARG abundances in upstream sites is not due to a complete absence of human fecal pollution but suggests the influence of additional environmental factors that were not measured in this study.

ARGs are associated with MGEs

The potential of ARGs to be transferred between cells was investigated by identifying ARGs located on MGEs such as plasmids, transposons, and integrative conjugative elements (ICEs). The number of unique ARGs encoded on MGEs (mARGs) was signif-

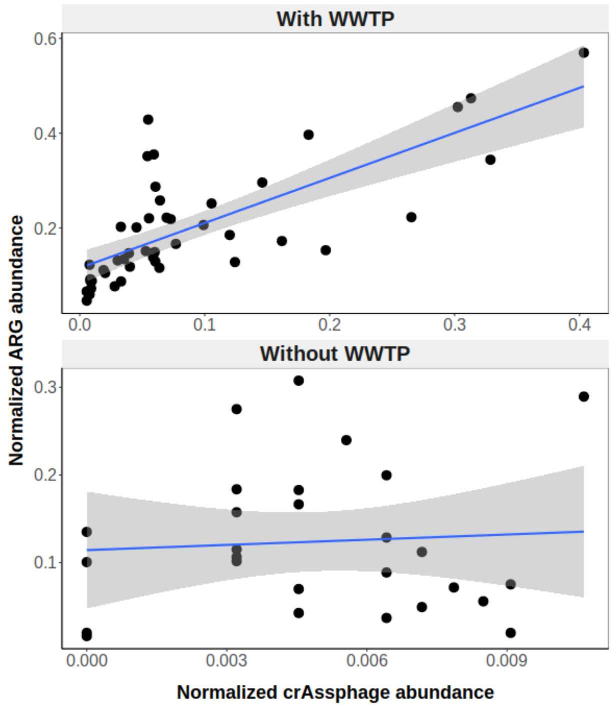

Figure 2. Relationship between ARG and crAssphage abundance. Normalized abundances of total ARG and crAssphage DNA were square root-transformed for analysis. Sites are grouped according to whether one or more WWTP is located upstream the sample site (top) or not (bottom). Smoothing curves based on linear regression (blue line) are shown along with 95% confidence intervals (shaded regions). Note the smaller range of crAssphage abundances in samples without upstream WWTPs.

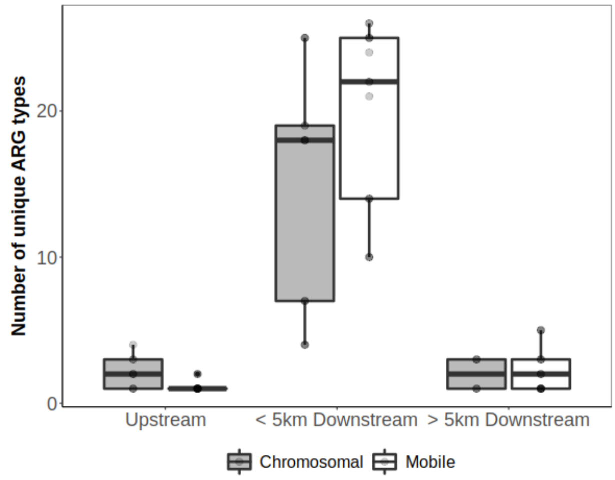

Figure 3. Diversity of ARGs and ARG-encoding plasmids/ICEs in relation to WWTP. Samples are grouped based on where they were collected relative to the nearest WWTP (Upstream = samples are of surface waters with no impact from a WWTP; <5 km downstream = samples were collected within 5 km downstream the nearest WWTP; >5 km downstream = samples were collected from sites greater than 5 km downstream nearest WWTP). The area between the lower and upper hinge represents the inter-quartile range (IQR), or difference between the first and third quartiles.

icantly higher at sites immediately downstream from WWTPs (Figure 3; randomization, $p < 0.001$). The number of mARGs rapidly diminished with increasing distance downstream (randomization, $p < 0.001$). On average, the number of mARGs immediately downstream from WWTPs (averaging 19 per sample) was slightly higher than the number of ARGs assumed to be chromosome-encoded due to lack of evidence to the contrary (cARG; averaging 15 per sample); however, the difference was not found to be significant (ANOVA, $p = 0.09$). Individual mARGs were more often than cARGs to be found in multiple sites downstream from WWTPs, consistent with the ability of mARGs to be shared among multiple bacterial species.

Of the 37 mARGs detected in the watershed, a majority (84%) could be found within 5 km downstream from a wastewater discharge site. Only two mARGs (blaTEM and sul1) were observed in sampling sites upstream from all WWTPs. The sulfonamide resistance gene sul1 was found in two distinct mobile contexts throughout the watershed (Supplementary Table 2), but in upstream sites, it appeared primarily in an integron that also contained the quaternary ammonium compound (QAC) resistance genes qacE and qacG (Figure 4). This integron had closest sequence similarity to integron In78 (100% identity over 60% of the sequence), previously associated with *Pseudomonas aeruginosa* [24].

Downstream sites hosted mobilized genes conferring resistance to many classes of antibiotic, including tetracycline, macrolide, aminoglycoside, fluoroquinolone, and lincosamide antibiotics as well as several likely plasmid/ICE-encoded genes responsible for the macrolide-lincosamide-streptogramin resistance phenotype (Supplementary Table 2). Unlike sul1, the plasmid-encoded sul2 variant was found exclusively downstream from WWTPs and in multiple different mobile contexts, often with a rolling-circle-type transposase. On one such contig with 100% identity to plasmid R485, sul2 was found together with a likely ISCR transposase, a toxin-antitoxin system, multiple conjugation proteins, and an integrase matching those from the Tn916 family of conjugative transposons.

Resistance phenotypes detected by antibiotic susceptibility assays

Escherichia coli colonies were cultivated from river water samples that were collected simultaneously with the samples for metagenomics sequencing. Curiously, no assembled metagenomic sequences were classified as *E. coli*, indicating that our sequencing and assembly approach was not sensitive enough to detect *E. coli* populations of this density.

E. coli colonies were tested for their susceptibility to a variety of antibiotics, including those associated with resistant Enterobacteriaceae pathogens identified in the 2013 CDC Antibiotic Resistance Threat Report as well as additional antibiotics with clinical significance. Antibiotic-resistant *E. coli* were isolated at 7 of the 24 sampling sites (Supplementary Table 3). Ampicillin resistance was the most common phenotype (6% of isolates), followed by amoxicillin-clavulanate, cefazolin, and cefoxitin resistance (3% of isolates each). No isolates were resistant to glycopeptide, trimethoprim, or sulfonamide antibiotics.

Colistin resistance was determined by measuring minimum inhibitory concentrations (MIC) with broth microdilution plates. The most frequently observed colistin MIC was 0.5 ug/mL. Three of the 70 isolates exhibited MICs of 2 ug mL^{-1}; colistin resistance is defined as a MIC >2 ug mL^{-1}. A single isolate exhibited a colistin MIC of 8 ug mL^{-1}. The metagenome of the site where this isolate was isolated (CPA) contains the colistin resistance gene mcr-5. No other genes conferring resistance to colistin were detected in the watershed.

Discussion

Antibiotic resistance genes (ARGs) have been previously detected in the discharge of wastewater treatment plants

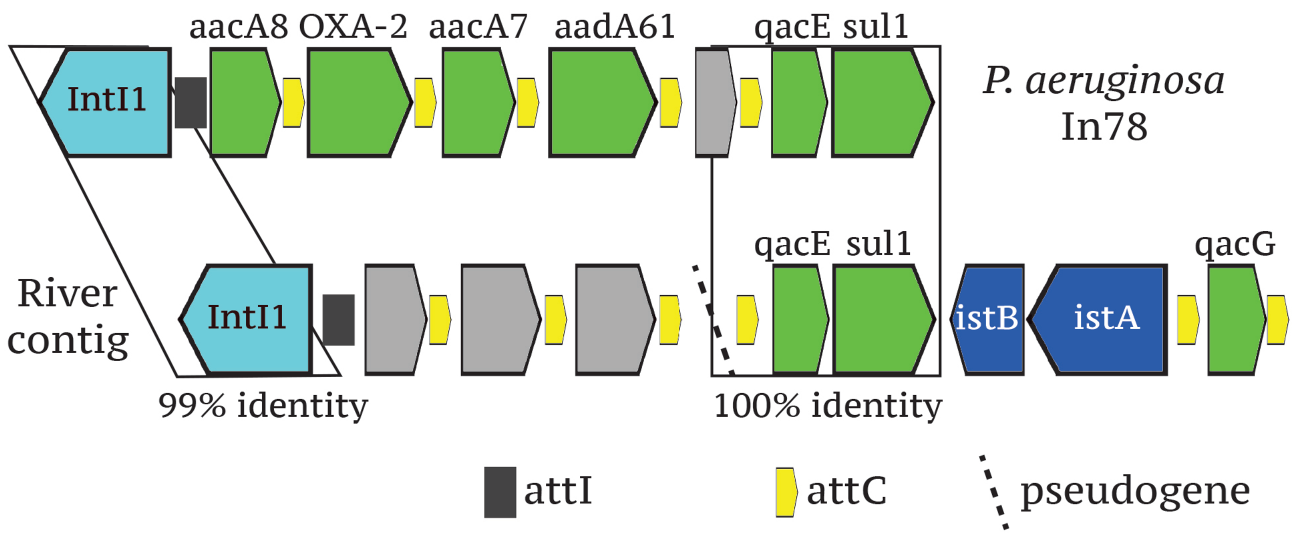

Figure 4. Novel sul1-bearing integron and closest match In78 from *P. aeruginosa*. In addition to the sul1-qacE cassette, the contig associated with the novel integron contained a full-length integron integrase and associated integrase and cassette attachment sites, 3 uncharacterized protein-coding genes, IS21-family transposition genes istA and istB, and a second QAC resistance gene (qacG). The boxed areas of the schema show the regions of alignment between integrons.

(WWTPs) [25, 26, 11, 8], as well as in receiving aquatic environments such as rivers and lakes [19, 27, 13, 14, 28, 29, 12]. Here, we conducted an extensive metagenomic study to investigate the distance decay effect of four wastewater treatment plants on the resistance profile of an urban watershed. River samples collected immediately downstream from WWTPs had significantly higher environmental abundance and diversity of ARGs, and this effect diminished in samples collected >5 km downstream. These results provide additional support for a localized effect of WWTPs on the resistome of receiving aquatic environments.

Our results also show that ARGs associated with WWTPs are likely to be encoded on mobile genetic elements (MGEs) such as plasmids and transposons. Nearly half of the unique ARGs detected downstream of the WWTPs were encoded on an MGE, and all of these ARGs have been previously associated with resistance to clinically relevant drug families. For instance, the erythromycin ribosome methylase (erm) genes have been identified as part of a core WWTP resistome [30], and have consistently been found enriched in biofilms and surface waters downstream from WWTP discharge [13, 12]. All of the erm genes detected in our study, including erm(F) and erm(B), were located in assembled sequences identified as plasmids or ICEs.

A number of other resistance determinants were also associated with MGEs. These included the linked aminoglycoside resistance genes strA and strB, which have previously been detected in WWTP effluent [28, 19] and other aquatic environments subjected to human pollutants [31]. The strA-strB genes are typically encoded on broad-host-range non-conjugative plasmids as well as conjugative plasmids associated with Tn3-type transposons [32]. Within the Blue River watershed, strA-strB was encoded on a complete Tn3-like transposon with 100% identity to transposon Tn5393d from *Alcaligenes faecalis*. Contigs containing the complete transposon were found at two sites, each downstream from a potential point source of ARG pollution (LDP and UMC), while smaller contigs containing a subset of this region were found downstream from all four WWTPs. The strA-strB genes were not found in any of the upstream samples.

The spectinomycin resistance gene aadA, another member of the core WWTP resistome described in Munck et al. (2015), was also found in samples downstream from all WWTPs and in no upstream samples. In our study, aadA genes frequently co-occurred with other ARGs and with multiple MGEs. For instance, aadA, sul1, qacEdelta, and an IS66 transposase were present on an assembled contig with >99% similarity to the conjugative tetracycline resistance plasmid pFBAOT6.

Plasmid-mediated quinolone resistance, conferred through the pentapeptide repeat protein qnr(S), was also detected immediately downstream from all WWTPs. The qnr(S) gene has consistently been found enriched in WWTP effluent and receiving waters [12, 14, 13].

In the two largest rivers investigated here, Blue River and Indian Creek, WWTPs contribute on average 15% of base flow [33], and could contribute over 95% under certain conditions [34]. Despite the significant contribution of WWTPs to both streamflow and ARG abundance and diversity, the WWTP-associated ARGs did not persist in surface waters more than 5 km downstream from effluent discharge points. This diminishing effect of elevated ARG abundances in receiving waters with increasing distance from WWTPs has been observed elsewhere [19, 27, 13]. These results, combined with the strong correlation of total ARG abundance with the abundance of a human gut phage, is consistent with the interpretation that ARGs were released into surface waters with human fecal pollution [35] and then diluted in downstream waters [19, 27].

The abundance of ARGs in sites upstream from all WWTPs, in contrast, were independent of human fecal pollution levels. The relatively high abundances of ARGs in these sites suggests the influence of agricultural pollution near the headwaters, but this hypothesis was not tested by the current study. The most abundant ARG in upstream samples was the sul1 sulfonamide resistance gene, which was primarily encoded on a class 1 integron along with two QAC resistance genes. This same mobile element was ubiquitous throughout the entire watershed, spanning multiple streamflows, land use types, and pollution levels, suggesting that it may be maintained in natural microbial communities due to a variety of selection pressures.

Potential implications

Our metagenomic study of an urban watershed has shown that 1) ARGs are widespread in natural aquatic environments, 2) WWTPs introduce a characteristic profile of mobilized ARGs into receiving rivers and streams, 3) Human fecal pollution and

associated ARGs can be effectively diluted by natural microbial populations within several kilometers from discharge points, and 4) the dispersal of specific ARG-encoding MGEs with conserved genomic structures can be traced throughout the watershed.

Characterization of the genomic context of ARGs, such as the novel, ubiquitous integron containing three different antimicrobial resistance genes, was enabled by assembly of the metagenomes, a computationally challenging task that is not routinely performed in such studies. The additional genomic information obtained from metagenomic assembly comes at the cost of reduced sensitivity of detection of sequences that have low abundances and those that are difficult to assemble. Nevertheless, the ability to detect ARGs in new genomic contexts and trace their dispersal among genetic elements, organisms, and environments is a powerful tool for the surveillance of antibiotic resistance in natural environments and potentially for the early detection of emerging resistance genotypes of clinical relevance. Future work should continue to develop and validate metagenomic methods for the quantitative measurement of antimicrobial resistance in natural environments.

Methods

Description of study sites

The Blue River watershed encompasses 450 square kilometers and includes the southern half of the Kansas City metropolitan area below the Missouri River. The majority of water in the Kansas City metropolitan area and in many of the adjacent towns to the south and west drains into the Blue River. The Blue River basin is primarily urban and suburban, with a population density of 794.8 persons/km^2. Six WWTPs are located in the basin, three of which near-continuously discharge treated effluent directly into the Blue River or one of its tributaries while a fourth has occasional wet-weather discharges into the lower Blue River. WWTPs provide the dominant source of streamflow, nutrients, and pharmaceutical compounds to the middle and lower reaches of the Blue River during base flow [33]. Twelve sites on the main stem of the Blue River, ten sites on the tributary Indian Creek, and two sites along Tomahawk Creek were investigated (Figure 5). Accessible locations bracketing WWTP discharges (n=4) were specifically targeted to capture the impact of WWTPs on the stream. Two sites along Tomahawk Creek, which does not contain any WWTPs, were sampled as additional upstream background samples for one WWTP site, which was located downstream from both the effluent discharge point and the confluence between Indian Creek and Tomahawk Creek. Additional samples from sites at various points throughout the watershed were collected as reference samples. Characteristics of the four WWTPs and a summary of the sampling sites are presented in Supplementary Tables 4 and 5, respectively.

Sample collection

A portable peristaltic pump was used to collect surface water samples by either pumping water directly from the stream/river into their respective containers or through sequential in-line filters. At each sampling site, the pump tubing inlet was submerged several centimeters beneath the water surface and held in place through the use of tube weights or anchors. An attempt was made to sample from a location in the stream/river where flow appeared to be greatest or most representative of total flow. Whenever possible pump tubing was positioned upstream to reduce unnecessary exposure to con-

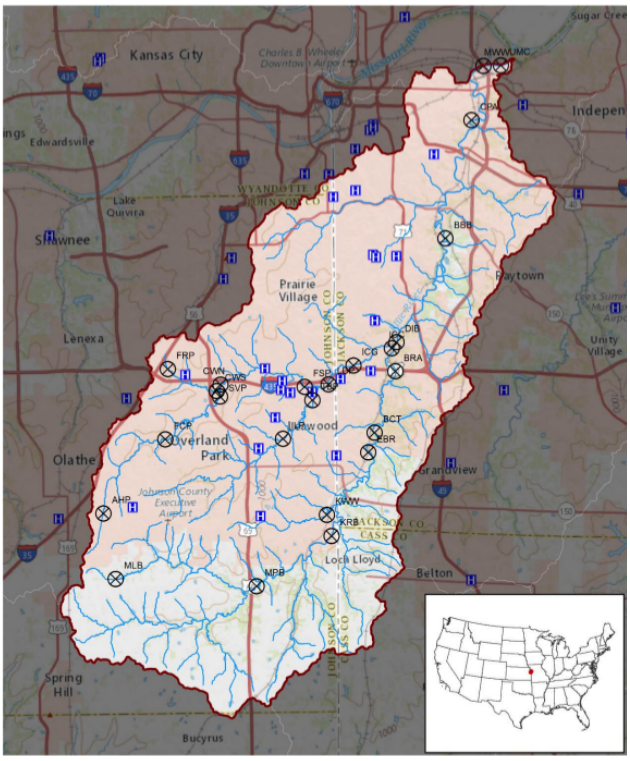

Figure 5. Location of sampling sites within the Blue River watershed. Sampling sites are marked with an X. Locations of hospitals are marked with a blue H.

taminants from sampling equipment and personnel, and caution was taken during the placement of the sampling equipment to avoid streambed disturbance upstream of the collection site. To avoid introducing contamination between sites the pump tubing was rinsed between sampling locations with ultrapure water and 80% ethanol and purged prior to sample collection with site water for at least one minute after placement of the tubing inlet. Physical and chemical parameters of the stream/river water, including dissolved oxygen, specific conductance, total dissolved solids, pH, oxidation-reduction potential, and temperature (Supplementary Table 6), were measured simultaneously with sample collection using a YSI Pro Plus portable multi-parameter water quality meter (YSI Incorporated), and turbidity was measured with a Mi415 turbidity meter (Martini instruments).

For each sample collected for metagenomic sequencing, site water was filtered sequentially through a 5.0 um mixed cellulose filter (5 um pore-size; 47 mm diameter; MF-Millipore; Millipore), followed by a 0.2 um Sterivex cartridge filter (0.22 um pore-size; Sterivex; Millipore), until either clogging of the filters substantially slowed water flow or a total volume of five liters had been filtered. This maximum volume was previously determined to be a reasonable compromise between expediency and thoroughness when sampling river water for metagenomic sequencing, despite considerable variation in sampling site water quality, and was found to be sufficient for this study as well. Depending on the level of suspended particles in the site waters, the volume of water filtered varied from 240 to 5000 mL. Volume filtered was an important factor affecting the amount of DNA that could be extracted from the filters and, consequently, the quality of a metagenomic dataset. A correction for difference in volume filtered was factored into calculations of gene abundance to account for this. Immediately after sampling, remaining fluid was purged from the filter assembly/cartridge and the 5.0 um and 0.2 um filters placed into separate, sterile whirlpool packs and frozen on dry ice before transportation

to the laboratory, where they were stored at -80 °C until processing. Field replicates, in triplicate, were collected at each sampling site and processed individually.

The 5.0 um prefilter was implemented as a screen for filtering plant debris and large eukaryotic cells to increase the coverage depth of genes associated with antibiotic resistance, which are exclusive to bacteria. In addition, the 5.0 um prefilter removed large particles, biofilms, and aggregates, which can unpredictably swamp samples of the free-living, suspended river communities collected by the 0.2 um filters. This smaller size fraction was the focus of the study, as it was considered to be more representative of suspended cells that are dispersing through the watershed rather than being retained in sediments and debris. No sequencing was performed with the 5 µm filters; therefore, genes encoded by large cells or cells associated with aggregates and biofilms are likely to be under-represented in our study.

Samples for *E. coli* enumeration and antibiotic susceptibility testing were collected into sterile, 100 mL clear plastic bottles and then serially diluted on-site in a phosphate buffer solution. Dilutions were filtered through a 0.45 um membrane filter and the filters aseptically removed from their assemblies and placed onto USEPA-approved chromogenic/fluorogenic media for the detection and enumeration of E. coli and total coliforms in water (U.S. Environmental Protection Agency Approved Method 1604: Total Coliforms and Escherichia coli in Water by Membrane Filtration Using a Simultaneous Detection Technique (MI Medium).). Inoculated plates were inverted and set to incubate at 35 °C for 20 to 24 hours. Following incubation, colonies exhibiting phenotypes typical of *E. coli* (blue-green colonies) were counted under ambient light using manufacturer's guidelines. Plates were then sealed with parafilm and stored in coolers with ice for transportation to the lab, where they were refrigerated for later culturing. Field blanks were collected at roughly one-quarter of the sites (chosen at random) and consisted of distilled water. Field blanks were processed in the same manner as the study samples. No growth was observed on all but one of the field blanks, which is suspected of having been contaminated with unsterilized forceps due to the unique pattern of growth.

Antibiotic susceptibility testing

Once in-lab, three colonies per sample that had been identified as *E. coli* on the MI medium were selected for subsequent phenotypic resistance testing. The chosen colonies were plated onto blood agar and incubated at 37 °C. The purity of the cultures was reassessed on chromogenic agar (red-pink colonies) whenever blood agar colonies exhibited atypical morphology. Three of the 74 plates made for susceptibility testing were found through this additional screening measure to be contaminated and removed from testing. Following incubation for approximately 24 hours, sensitivity of the remaining isolates to 16 antibiotics, including ampicillin, amoxicillin, cefazolin, cefepime, ceftriaxone, ceftazidime, ceftazidime with clavulanic acid, cefoxitin, cefotaxime, cefotaxime with clavulanic acid, meropenem, ciprofloxacin, aztreonam, sulfamethoxazole-trimethoprim, gentamicin and tetracycline, was determined through the Kirby-Bauer method with BBL Sensi-Disc Antimicrobial Susceptibility Test Discs (BD). Testing was performed according to CLSI protocols (CLSI, 2016) with the following modification: a BBL Prompt Inoculation System (BD) was used to pick between 3 and 5 colonies from the blood agar for standardized preparation of 0.5 McFarland turbidity level bacterial inocula. This procedure has been shown to have high correlation with conventional disk diffusion techniques, which involves the suspension of colonies in saline followed by dilution to 0.5 McFarland. Within 15 minutes of preparation, the suspension was used to inoculate Mueller-Hinton (MH) agar plates using standard swabbing technique. Between 5-6 antibiotic disks were applied to each of 3 plates per isolate to prevent overlapping zones. Inhibition zones were measured to the nearest millimeter for each of the 16 antibiotic disks by visual inspection of the inoculated plates following overnight incubation at 37 °C, and the zones interpreted through a Zone Diameter Interpretive Chart to determine susceptibility category.

Resistance to colistin was determined through broth microdilution (BMD). Because the large size of the colistin molecule prevents sufficient diffusion through agar medium, BMD is the only procedure recommended by the European Committee on Antimicrobial Susceptibility Testing (EUCAST) for antimicrobial susceptibility testing of colistin [36]. Minimum inhibitory concentration (MIC) for colistin was measured using MIC-strip colistin microdilution plates according to manufacturer's instruction (8 x 12 well plates; Merlin Diagnostics). Briefly, 50 ul of the standardized inoculum was added to 11 mL MH broth. The suspension was gently vortexed and a 100 ul aliquot per well transferred to microdilution strips containing MH broth supplemented with a 2-fold serial dilution of antibiotic. Final concentrations of antibiotic ranged from 0.0625 um/mL to 64 um/mL. Inoculated plates were sealed and incubated at 37 °C. Following incubation for 20-24 hours, MICs were determined through visual inspection of the plates under ambient and supplemented light. MIC was defined as the concentration of drug at which no growth was visible. Isolates were retested whenever a skipped well was observed during inspection of the BMD strips or for additional confirmation for *E. coli* isolates with MIC exceeding 2 ug/mL.

Metagenomic sequencing

Extraction of DNA from cells retained on the 0.2 um Sterivex filters was performed according to an established lab protocol [37], briefly described here. For the complete protocol, see "Availability of supporting data and materials". To help mitigate any variation that might be introduced during laboratory handling, DNA extraction was performed on the Sterivex filters in random order, with extraction order resolved beforehand. Filter cartridges were injected with extraction buffer, incubated at 65 °C for 30 minutes, and then the fluid was extracted from the filter cartridge and distributed into 0.1 mm glass bead tubes, which were shaken for 40 seconds in a MiniBeadBeater-16 607 (3450 oscillations/min; BioSpec). Bead tubes were centrifuged, and the supernatant was purified by extraction with phenol/chloroform/isoamyl alcohol (25:24:1) and chloroform/isoamyl alcohol (24:1). DNA was preciptated overnight at -20 °C in sodium-acetate/ethanol, and the pellets were resuspended in low-EDTA TE. DNA was not extracted from the 5 um prefilters, which remain in frozen storage for potential future analyses.

Whenever possible, 80 ng of purified DNA was used in the construction of metagenomic libraries. Total DNA extracted from the filters was quantified on a Qubit fluorometer (ThermoFisher). The quality of the sample DNA was also assessed before library construction with a Nanodrop spectrophotometer (ThermoFisher), and further purification conducted as needed based on Nanodrop 260/230 and 260/280 ratios using magnetic bead-based cleanup following a protocol adapted from Rohland & Reich (2012) [38]. The 260/230 curve was particularly predictive of library preparation success for this study, with a significant correlation found between Nanodrop 260/230 ratios and the final concentration of DNA in the prepared libraries (linear regression, adjusted $R^2 = 0.283$, $p = 5.267e-11$). Purified DNA

was fragmented by sonication with a Q800R sonicator (QSonica) at 4 °C, 25% amplitude, with a 10-second pulse for 60 seconds. These settings were selected to achieve a high molecular weight band on an agarose gel around the 500-700 base pair (bp) range. Magnetic beads were then used to select molecules from the fragmented DNA with a target size range of 500 to 700 bp. The size and quality of the fragments were verified with gel electrophoresis on a random subset of samples. Libraries were prepared from the size-selected DNA for metagenomic sequencing using the NEBnext Ultra DNA library prep kit for Illumina according to manufacturer instructions. A final bead cleanup was performed on the prepared DNA to remove excess kit reagents that could interfere with sequencing, and stored at -20 °C until transportation to the sequencing center. Each of the triplicate samples per site was processed separately, resulting in a total of 72 metagenomic libraries for sequencing.

Quality control and sequencing of the metagenomic libraries was conducted at the University of Utah High-Throughput Genomics Core Facility. Libraries were evaluated for quality on a Bioanalyzer DNA 1000 chip (Agilent Technologies), and then paired-end sequencing (2 x 125 bp) was performed on an Illumina HiSeq2500 platform with HiSeq v4 chemistry. Libraries were multiplexed and pooled 4 per lane for a total of 19 lanes of Illumina sequencing, yielding over one trillion bp of data (Supplementary Table 7). Demultiplexing and conversion of the raw sequencing base-call data were performed through the CASAVA v1.8 pipeline.

Sequence assembly

Quality control of the sequencing reads was performed with BBDuk v37.10 and consisted of contaminant removal and quality-based trimming. First, contaminants in the form of library adapter sequence were trimmed/filtered from the reads. An adapter was considered to be present whenever an adapter reference sequence shared a 23-mer with a read, or an 8-mer if located at the extreme 3' end. A Hamming distance of two was allowed between matching k-mers. Adapter sequences were also detected from completely overlapping read pairs. Additional contaminants in the form of PhiX DNA were identified and removed if 90% of a read's length was covered by the PhiX174 reference genome. PhiX DNA was used as a spike-in control during sequencing at concentrations representing approximately 0.5% of reads generated per lane of flow cell. Reads were compared to the reference genome using k-mer matching with a k-mer size of 31. Matching 31-mers were allowed to differ by a Hamming distance of one.

Reads were further trimmed based on quality using the Phred algorithm in BBDuk and discarded following trimming whenever the final length of a read was less than 52 bp. In a study using publicly available Illumina paired-end transcriptomes, it was found that overly stringent trimming resulted in worse assemblies according to the majority of metrics measured, particularly for low-coverage datasets [39]. The adverse effects of trimming were reduced, however, as coverage was increased. Based on these findings, a gentle trimming strategy was recommended (Phred quality score between 2 and 5) except under specific scenarios when more aggressive trimming is warranted, such as in the case of exceptionally high sequencing depth. We tested the applicability of these guidelines to metagenomic assemblies with a mock microbial community [40]. In nearly all cases, aggressive trimming reduced the number of mismatches and indels between the assembly and mapped short reads (Supplementary Figure 5), but there was an increasingly detrimental impact on measures of assembly performance as quality score threshold was increased, which was especially evident in the lower coverage libraries. Therefore, a low quality threshold was chosen for quality-based trimming (Phred quality score of 5), as the metagenomes were determined by Nonpareil v3.3.1 [41] to not be fully saturated with reads.

Records with both reads in a pair passing quality control were co-assembled by sampling site using Megahit v1.1.1 (Li et al. 2016) with parameters kstart=27, kend=127, and kstep=20, and taxonomic bins reconstructed from the resultant assemblies using PhyloPythiaS+ [42]. The performance of the assembler on the datasets was evaluated based on read mapping rates and statistics provided by MetaQuast v4.6.1 [43]. Megahit was selected for assembly because of its reliable performance on datasets of highly complex microbial communities [44, 45]. Megahit is also known to recover larger portions of strain variants than other, comparable short-read assemblers [46].

Identification and quantification of ARGs and MGEs

Contiguous sequences (or contigs) generated during assembly with length less than 200 bp were discarded prior to functional annotation. Putative coding DNA sequences were predicted from the remaining contigs and then translated into protein sequences using Prodigal v2.6.3 [47] in meta mode. Broad functional characterization of the gene predictions was performed through similarity searches against the Kyoto Encyclopedia of Genes and Genomes (KEGG, release 83.2) [48] using the BLASTP subcommand of Diamond v0.9.14 [49] with the more-sensitive flag, a maximum expect value of e-5, and parameters unal=0, max-target-seqs=1, strand=both. Homologs of known antibiotic resistance determinants were identified from the predicted protein sequences using AMRFinder v1.04 with default settings [21]. ARGs detected through AMRFinder with family type equal to "equivalog" or "subfamily" were excluded to ensure that only the highest confidence results were reported (see the AMRFinder documentation for a more thorough description of how ARGs are detected by AMRFinder).

The program hmmsearch (HMMER v3.1b) was used to detect remote homologs of mobile genetic element (MGE) markers by searching a custom database of profile HMMs against the predicted protein sequences using manually curated gathering scores to determine which hits to report. The database was built by supplementing a set of custom profiles with select profiles taken from Pfam v32.0 [50]. Markers selected for inclusion in the database were chosen for their involvement in the transposition of DNA (i.e., integrases and transposases), and represent integrons as well as type I and II transposable elements. To create the custom profiles, groups of related proteins belonging to the same marker family were downloaded from the NCBI RefSeq database of protein sequences. Duplicate sequences were clustered with CD-HIT, and very divergent (less than 20% identity) and very similar sequences (greater than 80% identity) were removed prior to initial multiple sequence alignment (MSA) with MAFFT [51]. Subsequent steps, including filtering, final alignment, and trimming, were performed with T-Coffee [52]. First, sequence CORE (sCORE) scores were generated, and the distribution of scores analyzed to identify outliers. Sequences with poor sCORE were discarded and the 40 most informative sequences extracted. The most informative sequences were defined as the sequences diverging the most from each other and constituted the final set used in the construction of the seed MSA. Residues from the seed MSA with low transitive consistency score [53] were removed, and the resulting curated alignments used to construct the profile HMMs. Model-specific cutoffs were assigned to the profiles as in Punta et al. (2012) [54].

Coverage of the identified features was estimated by individually mapping the quality-controlled short reads to their

relevant assembly using Bowtie2 v2.3.2 [55] with the very-sensitive flag and insert-size min and max parameters provided. These parameters were estimated from the insert-size mean and standard deviation calculated with BBmap v37.10. crAssphage coverage was calculated in an identical manner by mapping short reads to the crAssphage genome (GeneBank: NC_024711.1). To prevent artificial replicates from inflating coverage estimates, replicates in the mapped reads were first identified and removed using the MarkDuplicates functionality of Picard Tools v2.17.8. After duplicate filtering, mapped read best matches were sorted by name using Samtools v1.3 [56] and then used for estimating environmental abundance of the annotated sequences with count_features v1.3.0, part of the seq-annot open-source software package developed for the study.

For estimating feature abundances, read counts - the number of fragments thought to have originated from a given genomic region - were first transformed to fragment proportions (FP), a variant of transcripts per million (TPM) [57] without the application of a scaling factor. Each fragment used in the determination of the read counts was represented by a pair of aligned reads. The count of a feature was incremented whenever its coordinates fell within the interval of an alignment, defined as the region between two ends of a successfully mapped read pair, and an alignment interval overlapping multiple features was considered as separate evidence for the presence of each feature falling within its coordinates. Classes from the HTSeq python library v0.9.1 [58] were incorporated into count_features for storing alignment and feature coordinates.

FP is a measure of the proportion of fragments in the underlying population produced from a given genomic region. Raw counts were replaced with nucleotide fractions by dividing read counts by effective length of the feature. FP further varies from TPM in that the effective length of a feature is equal to its actual length. In metagenomes, any given position of a feature is capable of producing fragments of any length, so consideration of fragment start position is unnecessary in the determination of the counts per bp rate. The nucleotide fraction of each feature was divided by the sum of all counts per bp rates to give the length-adjusted proportion of feature i out of n total features predicted from sample k:

$$FP_{i,k} = \frac{\frac{counts_{i,k}}{length_i(bp)}}{\sum_i^{n_k} \frac{counts_{j,k}}{length_j(bp)}} \quad (1)$$

ARG abundances were normalized to the abundance of the rpoB gene by dividing the FP of each ARG to the FP of rpoB in that metagenomic library.

Detection of mobile ARGs

An assembly reconciliation program was used to merge contigs encoding one or more resistance genes prior to the detection of transposable ARGs. ARG-bearing contigs from across all sites were first combined and clustered at 100% identity. The dereplicated contigs were then merged using Mix v1.0 (Soueidan et al. 2013) with parameters C=100 and A=200. Merged contigs were manually inspected for mis-assembly by mapping the dereplicated contigs onto the merged assembly with BLASTN (BLAST+ v2.7.1) [59]. Contigs that failed to map were then appended to the merged assembly to create the final set of unique ARG-bearing contigs. Components of MGEs were predicted from these contigs as described above with additional annotation in the form of repeat units from Repseek v6.6 [60] and attC attachment sites using cmsearch (INFERNAL v1.1.2) [61] with the covariance model from Cury et al. (2016) [62]. Transposable ARGs were defined as those encoded by a complete intracellular MGE, including autonomous transposons, composite transposons, insertion sequence common regions (ISCRs), and integrons. To be considered complete, an MGE was required to contain its relevant recombination module and be bounded by appropriate attachment/excision sites when applicable. For instance, integron-encoded ARGs were required to co-occur on the same contig as both an attC recombination site as well as an integron integrase.

Transferable ARGs were detected by searching the identified ARGs against a database of plasmid and integrative and conjugative element (ICE) protein sequences using BLASTP with a reporting threshold of greater than 99% sequence similarity. The database was composed of sequences from the ACLAME v0.4 [63] and ICEberg v2.0 [64] databases and supplemented with additional plasmid sequences downloaded from NCBI RefSeq. For each site, contigs were re-mapped to the set of unique ARG-bearing contigs and the results, which included ARG-encoding plasmids/ICEs as well as transposable ARGs, were matched to the ARG abundance table for each metagenome. The proportion of mobile ARG per metagenome was calculated as the abundance of transferable or transposable ARGs over the total abundance of ARGs.

Statistical analyses

All statistical analyses were performed in the R programming language v3.5.1 (R Development Core Team 2008) with the aid of reshape2 v1.4.3 [65] and several graphing and statistical libraries. Figures were generated with the data visualization library ggplot2 v3.1.0 [66], using color palettes from the dichromat package v2.0.0 [67]. Resistome percentages were calculated as the fraction of total normalized abundance for a given ARG or ARG category out of the total normalized abundance for all ARGs detected within the watershed samples. ARG diversity was calculated as the number of different ARG types detected at a site or within the watershed, where a given ARG type represents a unique entry in the AMRFinder database at the allele or exception level.

Linear regressions were performed with the modeling function lm (core stats package), and curves drawn using the geom_smooth layer of ggplot2 with linear model (lm) set as the smoothing method. Normalized ARG abundance was modeled as a linear function of the normalized abundance of crAssphage DNA. To determine if the relationship was dependent on input from known sources of human waste, samples were grouped based on whether they were collected from a site downstream from one or more WWTPs or from a site with no upstream WWTP. A linear model of ARG and crAssphage abundances was compared to one that included an additional interaction term separating samples based on whether they were taken from sites located downstream from WWTPs or from sites upstream from all WWTPs. The model that included the interaction term was found to better fit the data (ANOVA, $p = 0.001927$), so the relationship between ARG and crAssphage abundances within sample groups was further tested individually. Normalized abundances were log-transformed prior to model fitting.

Geographic distance served as a proxy for river distance when assigning sites to one of three groups representing varying distances from WWTPs. Normalized coverage totals were log-transformed for between-group comparisons, and differences in total abundance assessed with one-way analysis of variance (ANOVA) followed by Tukey's posthoc test for pairwise comparisons of group means. When abundance data were further divided by location of the resistance gene (chromoso-

mal versus mobilized), the log-transformed data failed to meet test assumptions of normally distributed data with equal variance; in this case, significance was instead assessed through a non-parametric randomization procedure involving randomly reassigned sample labels. P-values were calculated by comparing observed F values with distributions generated from ten thousand permutations of the data. The level of statistical significance was set at 0.001 for all statistical tests performed.

Only samples collected from sites located upstream and within 5 km downstream from a WWTP were considered in the analysis of differential abundance (n=18 and n=15 per group, respectively). The change in abundance of ARG totals at downstream WWTP sites was calculated as the log2 of the ratio between downstream and upstream abundance, and statistical significance of differences in abundance between groups assessed using edgeR v3.24.3 [68] on unmodified read counts. Gene categories with False Discovery Rate (FDR) less than 0.05 were considered to be significantly more abundant in one group over the other.

Availability of source code and requirements

- Project name: seq-annot
- Project home page: e.g. https://github.com/Brazelton-Lab/seq-annot
- Operating system(s): Linux
- Programming language: Python
- Other requirements: Python 3.4 or higher
- License: GPLv3
- biotoolsID: biotools:seq-annot
- RRID: SCR_018731
- Any restrictions to use by non-academics: none

Availability of supporting data and materials

The raw metagenomic sequence data sets and the metagenome assemblies supporting the results reported here are available at the NCBI Sequence Read Archive and NCBI WGS, respectively, under BioProject accession PRJNA562643. The protocols used in the extraction of sample DNA can be found at https://baas-becking.biology.utah.edu/data/category/18-protocols. The custom software and scripts used in data processing are available from https://github.com/Brazelton-Lab. The R code used in data analysis is also available at the development GitHub page https://github.com/Brazelton-Lab/Thornton_2019.

Additional files

Additional File 1: Supplementary tables and figures.

Supplementary Table 1. Percent total watershed resistome by drug class.

Supplementary Table 2. ARG-bearing contigs matching known mobile genetic elements.

Supplementary Table 3. Summary of the antibiotic susceptibility testing results.

Supplementary Table 4. Characteristics of the four WWTPs associated with the study.

Supplementary Table 5. Description of sites sampled within the Blue River Watershed.

Supplementary Table 6. Physical and chemical parameters of sample site surface waters.

Supplementary Table 7. Sequencing and assembly statistics.

Supplementary Figure 1. Total normalized ARG abundance by sampling site.

Supplementary Figure 2. Total ARG abundance at varying distances from WWTP.

Supplementary Figure 3. ARG richness with increasing distance downstream a WWTP.

Supplementary Figure 4. Total MGE abundance at varying distances from WWTP.

Supplementary Figure 5. Association between assembly quality and quality score threshold at varying sequencing depths.

Additional File 2: Results of the statistical tests using an alternative normalization measure.

Figure A.S1. Total normalized ARG abundance by sampling site.

Figure A.1. Total abundance of ARGs in relation to proximity to WWTP

Figure A.S1. Total ARG abundance at varying distances from WWTP.

Figure A.2. Relationship between ARG and crAssphage abundance.

Declarations

List of abbreviations

ANOVA: Analysis of Variance; ARB: Antibiotic Resistant Bacteria; ARG: Antibiotic Resistance Gene; AS: Activated Sludge; BMD: Broth Microdilution; BNR: Biological Nutrient Removal; bp: Base Pairs; CARD: Comprehensive Antibiotic Resistance Database; cARG: Chromosomal Antibiotic Resistance Gene; crAssphage: cross-assembly phage; EUCAST: European Committee on Antimicrobial Susceptibility Testing; FDR: False Discovery Rate; FP: Fragment Proportions; HGT: Horizontal Gene Transfer; HMM: Hidden Markov Model; ICE: Integrative and Conjugative Element; ISCR: Insertion Sequence Common Region; KEGG: Kyoto Encyclopedia of Genes and Genomes; LSa: Lincosamide-Steptogramin a mARG: Mobile Antibiotic Resistance Gene; MGD: Million Gallons per Day; MGE: Mobile Genetic Element; MH: Mueller-Hinton; MIC: Minimum Inhibitory Concentration; ML: Macrolide-Lincosamide; MLSb: Macrolide-Lincosamide-Streptogramin B; MSb: Macrolide-Streptogramin B; MSA: Multiple Sequence Alignment; QAC: Quaternary Ammonium Compound; PE: Person Equivalents; TF: Trickle Filter; TF/GC: Trickle Filter with Gravity Clarifiers; TPM: Transcripts per Million; WWTP: Wastewater Treatment Plant.

Ethical Approval

Not applicable.

Consent for publication

Not applicable.

Competing Interests

The authors declare that they have no competing interests.

Funding

This work was supported by contract #200-2016-91949 to PI VanDerslice from the US Centers for Disease Control and Prevention under Broad Agency Announcement 2016-N-17812. The funding body had no role in the design or execution of the study, nor in writing the manuscript.

Author's Contributions

W.J.B. conceptualized the project, designed the experiments, and wrote the manuscript. C.N.T. assisted in the experimental design, performed the experiments, analyzed the data, developed the software, and wrote the manuscript. J.A.V. conceptualized the project and designed the experiments. W.D.T. conceptualized the project and designed and performed the experiments. All authors assisted in data collection and edited the manuscript.

Acknowledgements

We would like to thank Bahvneet Singh, Alex Hyer, and Cody Dangerfield for their assistance in sample collection. In addition, Sharzad Motamedi, Julia McGonigle, and Lizethe Pendleton provided invaluable advice during sample processing and preparation for sequencing.

References

1. Doi Y, Park YS, Rivera JI, Adams-Haduch JM, Hingwe A, Sordillo EM, et al. Community-Associated Extended-Spectrum B-Lactamase–Producing Escherichia coli Infection in the United States. Clinical Infectious Diseases 2013 Mar;56(5):641–648. https://academic.oup.com/cid/article-lookup/doi/10.1093/cid/cis942.
2. Freeman J, Sexton D, Anderson D. Emergence of Extended-Spectrum B-Lactamase–Producing *Escherichia coli* in Community Hospitals throughout North Carolina: A Harbinger of a Wider Problem in the United States? Clinical Infectious Diseases 2009 Jul;49(2):e30–e32. https://academic.oup.com/cid/article-lookup/doi/10.1086/600046.
3. Chen B, Yang Y, Liang X, Yu K, Zhang T, Li X. Metagenomic Profiles of Antibiotic Resistance Genes (ARGs) between Human Impacted Estuary and Deep Ocean Sediments. Environmental Science & Technology 2013 Nov;47(22):12753–12760. http://pubs.acs.org/doi/10.1021/es403818e.
4. Bhullar K, Waglechner N, Pawlowski A, Koteva K, Banks ED, Johnston MD, et al. Antibiotic Resistance Is Prevalent in an Isolated Cave Microbiome. PLoS ONE 2012 Apr;7(4):e34953. http://dx.plos.org/10.1371/journal.pone.0034953.
5. D'Costa VM, King CE, Kalan L, Morar M, Sung WWL, Schwarz C, et al. Antibiotic resistance is ancient. Nature 2011 Aug;477(7365):457–461. http://www.nature.com/doifinder/10.1038/nature10388.
6. Lupo A, Coyne S, Berendonk TU. Origin and Evolution of Antibiotic Resistance: The Common Mechanisms of Emergence and Spread in Water Bodies. Frontiers in Microbiology 2012;3. http://journal.frontiersin.org/article/10.3389/fmicb.2012.00018/abstract.
7. Guo J, Li J, Chen H, Bond PL, Yuan Z. Metagenomic analysis reveals wastewater treatment plants as hotspots of antibiotic resistance genes and mobile genetic elements. Water Research 2017 Oct;123:468–478. https://linkinghub.elsevier.com/retrieve/pii/S0043135417305651.
8. Munir M, Wong K, Xagoraraki I. Release of antibiotic resistant bacteria and genes in the effluent and biosolids of five wastewater utilities in Michigan. Water Research 2011 Jan;45(2):681–693. https://linkinghub.elsevier.com/retrieve/pii/S004313541000597X.
9. Su JQ, An XL, Li B, Chen QL, Gillings MR, Chen H, et al. Metagenomics of urban sewage identifies an extensively shared antibiotic resistome in China. Microbiome 2017 Jul;5. https://www.ncbi.nlm.nih.gov/pmc/articles/PMC5517792/.
10. Yang Y, Li B, Zou S, Fang HHP, Zhang T. Fate of antibiotic resistance genes in sewage treatment plant revealed by metagenomic approach. Water Research 2014 Oct;62:97–106. https://linkinghub.elsevier.com/retrieve/pii/S0043135414003728.
11. Mao D, Yu S, Rysz M, Luo Y, Yang F, Li F, et al. Prevalence and proliferation of antibiotic resistance genes in two municipal wastewater treatment plants. Water Research 2015 Nov;85:458–466. https://linkinghub.elsevier.com/retrieve/pii/S0043135415302220.
12. Marti E, Jofre J, Balcazar JL. Prevalence of Antibiotic Resistance Genes and Bacterial Community Composition in a River Influenced by a Wastewater Treatment Plant. PLoS ONE 2013 Oct;8(10):e78906. https://dx.plos.org/10.1371/journal.pone.0078906.
13. Proia L, von Schiller D, Sànchez-Melsió A, Sabater S, Borrego CM, Rodríguez-Mozaz S, et al. Occurrence and persistence of antibiotic resistance genes in river biofilms after wastewater inputs in small rivers. Environmental Pollution 2016 Mar;210:121–128. http://linkinghub.elsevier.com/retrieve/pii/S0269749115301950.
14. Subirats J, Triadó-Margarit X, Mandaric L, Acuña V, Balcázar JL, Sabater S, et al. Wastewater pollution differently affects the antibiotic resistance gene pool and biofilm bacterial communities across streambed compartments. Molecular Ecology 2017 Oct;26(20):5567–5581. http://doi.wiley.com/10.1111/mec.14288.
15. Gullberg E, Albrecht LM, Karlsson C, Sandegren L, Andersson DI. Selection of a Multidrug Resistance Plasmid by Sublethal Levels of Antibiotics and Heavy Metals. mBio 2014 Oct;5(5):e01918–14. https://mbio.asm.org/lookup/doi/10.1128/mBio.01918-14.
16. Rizzo L, Manaia C, Merlin C, Schwartz T, Dagot C, Ploy MC, et al. Urban wastewater treatment plants as hotspots for antibiotic resistant bacteria and genes spread into the environment: A review. Science of The Total Environment 2013 Mar;447:345–360. https://linkinghub.elsevier.com/retrieve/pii/S0048969713000429.
17. Petrovich M, Chu B, Wright D, Griffin J, Elfeki M, Murphy BT, et al. Antibiotic resistance genes show enhanced mobilization through suspended growth and biofilm-based wastewater treatment processes. FEMS Microbiology Ecology 2018 May;94(5). https://academic.oup.com/femsec/article/doi/10.1093/femsec/fiy041/4925569.
18. Yuan QB, Guo MT, Wei WJ, Yang J. Reductions of bacterial antibiotic resistance through five biological treatment processes treated municipal wastewater. Environmental science and pollution research international 2016 Oct;23(19):19495–19503. Place: Germany.
19. Chu BTT, Petrovich ML, Chaudhary A, Wright D, Murphy

B, Wells G, et al. Metagenomics Reveals the Impact of Wastewater Treatment Plants on the Dispersal of Microorganisms and Genes in Aquatic Sediments. Applied and Environmental Microbiology 2017 Dec;84(5):e02168-17, /aem/84/5/e02168-17.atom. http://aem.asm.org/lookup/doi/10.1128/AEM.02168-17.
20. The Global Sewage Surveillance project consortium, Hendriksen RS, Munk P, Njage P, van Bunnik B, McNally L, et al. Global monitoring of antimicrobial resistance based on metagenomics analyses of urban sewage. Nature Communications 2019 Dec;10(1):1124. http://www.nature.com/articles/s41467-019-08853-3.
21. Feldgarden M, Brover V, Haft DH, Prasad AB, Slotta DJ, Tolstoy I, et al. Using the NCBI AMRFinder Tool to Determine Antimicrobial Resistance Genotype-Phenotype Correlations Within a Collection of NARMS Isolates. Microbiology; 2019.
22. Dutilh BE, Cassman N, McNair K, Sanchez SE, Silva GGZ, Boling L, et al. A highly abundant bacteriophage discovered in the unknown sequences of human faecal metagenomes. Nature Communications 2014 Dec;5(1):4498. http://www.nature.com/articles/ncomms5498.
23. García-Aljaro C, Ballesté E, Muniesa M, Jofre J. Determination of crAssphage in water samples and applicability for tracking human faecal pollution. Microbial Biotechnology 2017 Nov;10(6):1775–1780. http://doi.wiley.com/10.1111/1751-7915.12841.
24. Aubert D, Girlich D, Naas T, Nagarajan S, Nordmann P. Functional and Structural Characterization of the Genetic Environment of an Extended-Spectrum -Lactamase blaVEB Gene from a Pseudomonas aeruginosa Isolate Obtained in India. Antimicrobial Agents and Chemotherapy 2004 Sep;48(9):3284–3290. http://aac.asm.org/cgi/doi/10.1128/AAC.48.9.3284-3290.2004.
25. Karkman A, Johnson TA, Lyra C, Stedtfeld RD, Tamminen M, Tiedje JM, et al. High-throughput quantification of antibiotic resistance genes from an urban wastewater treatment plant. FEMS Microbiology Ecology 2016 Mar;92(3):fiw014. https://academic.oup.com/femsec/article-lookup/doi/10.1093/femsec/fiw014.
26. Tang J, Bu Y, Zhang XX, Huang K, He X, Ye L, et al. Metagenomic analysis of bacterial community composition and antibiotic resistance genes in a wastewater treatment plant and its receiving surface water. Ecotoxicology and Environmental Safety 2016 Oct;132:260–269. https://linkinghub.elsevier.com/retrieve/pii/S0147651316302226.
27. Czekalski N, Gascón Díez E, Bürgmann H. Wastewater as a point source of antibiotic-resistance genes in the sediment of a freshwater lake. The ISME Journal 2014 Jul;8(7):1381–1390. http://www.nature.com/articles/ismej20148.
28. Kristiansson E, Fick J, Janzon A, Grabic R, Rutgersson C, Weijdegård B, et al. Pyrosequencing of Antibiotic-Contaminated River Sediments Reveals High Levels of Resistance and Gene Transfer Elements. PLoS ONE 2011 Feb;6(2):e17038. http://dx.plos.org/10.1371/journal.pone.0017038.
29. Rowe W, Verner-Jeffreys DW, Baker-Austin C, Ryan JJ, Maskell DJ, Pearce GP. Comparative metagenomics reveals a diverse range of antimicrobial resistance genes in effluents entering a river catchment. Water Science and Technology 2016 Apr;73(7):1541–1549. https://iwaponline.com/wst/article/73/7/1541-1549/19028.
30. Munck C, Albertsen M, Telke A, Ellabaan M, Nielsen P, Sommer M. Limited dissemination of the wastewater treatment plant core resistome. Nature Communications 2015 Sep;6.
31. Bengtsson-Palme J, Boulund F, Fick J, Kristiansson E, Larsson DGJ. Shotgun metagenomics reveals a wide array of antibiotic resistance genes and mobile elements in a polluted lake in India. Frontiers in Microbiology 2014 Dec;5. http://journal.frontiersin.org/article/10.3389/fmicb.2014.00648/abstract.
32. Sundin GW, Bender CL. Dissemination of the strA-strB streptomycin-resistance genes among commensal and pathogenic bacteria from humans, animals, and plants. Molecular Ecology 1996 Feb;5(1):133–143. http://doi.wiley.com/10.1111/j.1365-294X.1996.tb00299.x.
33. Wilkison DH, Armstrong DJ, Blevins DW. Effects of wastewater and combined sewer overflows on water quality in the Blue River basin, Kansas City, Missouri and Kansas, July 1998-October 2000. US Geological Survey Water-Resources Investigations Report 2002;p. 162.
34. Wilkison DH. Water Quality in the Blue River Basin, Kansas City Metropolitan Area, Missouri and Kansas, July 1998 to October 2004. US Geological Survey Scientific Investigations Report 2006;p. 6.
35. Karkman A, Pärnänen K, Larsson DGJ. Fecal pollution can explain antibiotic resistance gene abundances in anthropogenically impacted environments. Nature Communications 2019 Dec;10(1). http://www.nature.com/articles/s41467-018-07992-3.
36. Matuschek E, Åhman J, Webster C, Kahlmeter G. Antimicrobial susceptibility testing of colistin – evaluation of seven commercial MIC products against standard broth microdilution for Escherichia coli , Klebsiella pneumoniae , Pseudomonas aeruginosa , and Acinetobacter spp. Clinical Microbiology and Infection 2018 Aug;24(8):865–870. https://linkinghub.elsevier.com/retrieve/pii/S1198743X17306675.
37. Brazelton WJ, Thornton CN, Hyer A, Twing KI, Longino AA, Lang SQ, et al. Metagenomic identification of active methanogens and methanotrophs in serpentinite springs of the Voltri Massif, Italy. PeerJ 2017 Jan;5:e2945. https://peerj.com/articles/2945.
38. Rohland N, Reich D. Cost-effective, high-throughput DNA sequencing libraries for multiplexed target capture. Genome Research 2012 May;22(5):939–946. http://genome.cshlp.org/cgi/doi/10.1101/gr.128124.111.
39. MacManes MD. On the optimal trimming of high-throughput mRNA sequence data. Frontiers in Genetics 2014;5. http://journal.frontiersin.org/article/10.3389/fgene.2014.00013/abstract.
40. Shakya M, Quince C, Campbell JH, Yang ZK, Schadt CW, Podar M. Comparative metagenomic and rRNA microbial diversity characterization using archaeal and bacterial synthetic communities: Metagenomic and rRNA diversity characterization. Environmental Microbiology 2013 Jun;15(6):1882–1899. http://doi.wiley.com/10.1111/1462-2920.12086.
41. Rodriguez-R LM, Konstantinidis KT. Nonpareil: a redundancy-based approach to assess the level of coverage in metagenomic datasets. Bioinformatics 2014 Mar;30(5):629–635. https://academic.oup.com/bioinformatics/article-lookup/doi/10.1093/bioinformatics/btt584.
42. Gregor I, Dröge J, Schirmer M, Quince C, McHardy AC. *PhyloPythiaS+* : a self-training method for the rapid reconstruction of low-ranking taxonomic bins from metagenomes. PeerJ 2016 Feb;4:e1603. https://peerj.com/articles/1603.
43. Mikheenko A, Saveliev V, Gurevich A. MetaQUAST: evaluation of metagenome assemblies. Bioinformatics 2016 Apr;32(7):1088–1090. https://academic.oup.com/bioinformatics/article-lookup/doi/10.1093/bioinformatics/btv697.

44. van der Walt AJ, van Goethem MW, Ramond JB, Makhalanyane TP, Reva O, Cowan DA. Assembling metagenomes, one community at a time. BMC Genomics 2017 Dec;18(1). http://bmcgenomics.biomedcentral.com/articles/10.1186/s12864-017-3918-9.
45. Vollmers J, Wiegand S, Kaster AK. Comparing and Evaluating Metagenome Assembly Tools from a Microbiologist's Perspective - Not Only Size Matters! PLOS ONE 2017 Jan;12(1):e0169662. http://dx.plos.org/10.1371/journal.pone.0169662.
46. Awad S, Irber L, Brown CT. Evaluating Metagenome Assembly on a Simple Defined Community with Many Strain Variants. bioRxiv 2017;p. 155358.
47. Hyatt D, Chen GL, LoCascio PF, Land ML, Larimer FW, Hauser LJ. Prodigal: prokaryotic gene recognition and translation initiation site identification. BMC Bioinformatics 2010 Mar;11:119. https://www.ncbi.nlm.nih.gov/pmc/articles/PMC2848648/.
48. Ogata H, Goto S, Sato K, Fujibuchi W, Bono H, Kanehisa M. KEGG: Kyoto Encyclopedia of Genes and Genomes. Nucleic Acids Research 1999 Jan;27(1):29–34. https://academic.oup.com/nar/article-lookup/doi/10.1093/nar/27.1.29.
49. Buchfink B, Xie C, Huson DH. Fast and sensitive protein alignment using DIAMOND. Nature Methods 2014 Nov;12(1):59–60. http://www.nature.com/doifinder/10.1038/nmeth.3176.
50. El-Gebali S, Mistry J, Bateman A, Eddy SR, Luciani A, Potter SC, et al. The Pfam protein families database in 2019. Nucleic Acids Research 2019 Jan;47(D1):D427–D432. https://academic.oup.com/nar/article/47/D1/D427/5144153.
51. Katoh K. MAFFT: a novel method for rapid multiple sequence alignment based on fast Fourier transform. Nucleic Acids Research 2002 Jul;30(14):3059–3066. https://academic.oup.com/nar/article-lookup/doi/10.1093/nar/gkf436.
52. Notredame C, Higgins DG, Heringa J. T-coffee: a novel method for fast and accurate multiple sequence alignment 1 1Edited by J. Thornton. Journal of Molecular Biology 2000 Sep;302(1):205–217. https://linkinghub.elsevier.com/retrieve/pii/S0022283600940427.
53. Chang JM, Di Tommaso P, Notredame C. TCS: A New Multiple Sequence Alignment Reliability Measure to Estimate Alignment Accuracy and Improve Phylogenetic Tree Reconstruction. Molecular Biology and Evolution 2014 Jun;31(6):1625–1637. https://academic.oup.com/mbe/article-lookup/doi/10.1093/molbev/msu117.
54. Punta M, Coggill PC, Eberhardt RY, Mistry J, Tate J, Boursnell C, et al. The Pfam protein families database. Nucleic Acids Research 2012 Jan;40(D1):D290–D301. https://academic.oup.com/nar/article-lookup/doi/10.1093/nar/gkr1065.
55. Langmead B, Salzberg SL. Fast gapped-read alignment with Bowtie 2. Nature Methods 2012 Apr;9(4):357–359. http://www.nature.com/articles/nmeth.1923.
56. Li H, Handsaker B, Wysoker A, Fennell T, Ruan J, Homer N, et al. The Sequence Alignment/Map format and SAMtools. Bioinformatics 2009 Aug;25(16):2078–2079. https://academic.oup.com/bioinformatics/article-lookup/doi/10.1093/bioinformatics/btp352.
57. Li B, Ruotti V, Stewart RM, Thomson JA, Dewey CN. RNA-Seq gene expression estimation with read mapping uncertainty. Bioinformatics 2010 Feb;26(4):493–500. https://academic.oup.com/bioinformatics/article-lookup/doi/10.1093/bioinformatics/btp692.
58. Anders S, Pyl PT, Huber W. HTSeq–a Python framework to work with high-throughput sequencing data. Bioinformatics 2015 Jan;31(2):166–169. https://academic.oup.com/bioinformatics/article-lookup/doi/10.1093/bioinformatics/btu638.
59. Camacho C, Coulouris G, Avagyan V, Ma N, Papadopoulos J, Bealer K, et al. BLAST+: architecture and applications. BMC Bioinformatics 2009;10(1):421. http://www.biomedcentral.com/1471-2105/10/421.
60. Achaz G, Boyer F, Rocha EPC, Viari A, Coissac E. Repseek, a tool to retrieve approximate repeats from large DNA sequences. Bioinformatics 2007 Jan;23(1):119–121. https://academic.oup.com/bioinformatics/article-lookup/doi/10.1093/bioinformatics/btl519.
61. Nawrocki EP, Eddy SR. Infernal 1.1: 100-fold faster RNA homology searches. Bioinformatics 2013 Nov;29(22):2933–2935. https://academic.oup.com/bioinformatics/article-lookup/doi/10.1093/bioinformatics/btt509.
62. Cury J, Jové T, Touchon M, Néron B, Rocha EP. Identification and analysis of integrons and cassette arrays in bacterial genomes. Nucleic Acids Research 2016 Jun;44(10):4539–4550. https://www.ncbi.nlm.nih.gov/pmc/articles/PMC4889954/.
63. Leplae R, Lima-Mendez G, Toussaint A. ACLAME: A CLAssification of Mobile genetic Elements, update 2010. Nucleic Acids Research 2010 Jan;38(suppl_1):D57–D61. https://academic.oup.com/nar/article-lookup/doi/10.1093/nar/gkp938.
64. Liu M, Li X, Xie Y, Bi D, Sun J, Li J, et al. ICEberg 2.0: an updated database of bacterial integrative and conjugative elements. Nucleic Acids Research 2019 Jan;47(D1):D660–D665. https://academic.oup.com/nar/article/47/D1/D660/5165266.
65. Wickham H. Reshaping Data with the **reshape** Package. Journal of Statistical Software 2007;21(12). http://www.jstatsoft.org/v21/i12/.
66. Wickham H. ggplot2: Elegant Graphics for Data Analysis. Springer-Verlag New York; 2016. https://ggplot2.tidyverse.org.
67. Lumley T, Knoblauch K, Waichler S, Zeileis A, dichromat: Color Schemes for Dichromats; 2013. https://CRAN.R-project.org/package=dichromat.
68. Robinson MD, McCarthy DJ, Smyth GK. edgeR: a Bioconductor package for differential expression analysis of digital gene expression data. Bioinformatics 2010 Jan;26(1):139–140. https://academic.oup.com/bioinformatics/article-lookup/doi/10.1093/bioinformatics/btp616.

supplementary file

Click here to access/download
Supplementary Material
Additional File 1.pdf
