# Supplementary material for: Localized effect of treated wastewater effluent on the resistome of an urban watershed
Source: Gigascience. 2020 Nov 19;9(11):giaa125. doi: 10.1093/gigascience/giaa125 (PMC7677451; doi:10.1093/gigascience/giaa125)
Supplement: giaa125_Supplemental_File [file giaa125_supplemental_file.pdf]

Supplementary Table 1: Percent total watershed resistome by drug class.

Drug Class	Watershed	Blue River	Indian Creek	Indian Creek North	Tomahawk Creek
Aminoglycoside	24.0	15.6	38.8	1.83	0
Beta-lactam	15.9	30.9	12.7	68.2	3.04
Diaminopyrimidine	0.4	0.4	0.1	5.8	0
Fluoroquinolone	0.5	0.2	1.0	0	0
Glycopeptide	0.4	0	0	0	0
Lincosamide	2.0	3.0	0.8	0	0
LSa	0.2	0.3	0	0	0
Macrolide	13.6	16.8	12.6	0	0
MLS	2.6	3.7	1.8	0	0
MSb	4.8	6.3	4.1	0	0
Multidrug	0.3	0	0	13.8	0
Phenicol	0.1	0.3	0	0	0
Polypeptide	0.03	0.1	0	0	0
Rifamycin	0.02	0	0	0.9	0
Sulfone-Sulfonamide	26.0	26.8	20.7	9.4	97.0
Tetracycline	9.4	10.6	7.6	0	0

Supplementary Table 2: ARG-bearing contigs matching known mobile genetic elements.

Contig ID	MGE Type	MGE Components	ARG genes	Complete
gc_10	ICE	None	tetQ	N
gc_12	In	IntI, istAB	sul1, qacEG	Y
gc_15	Plasmid	None	blaA	N
gc_22	Tn916	IntTn, xisTn	tetM	Y
gc_26	Plasmid	IS66, res	tet, mph, msr	Y
gc_33	ISCR	IS91	sul2	Y
gc_35	Plasmid	Int, relaxase, trfA, mobC	qacL, blaOXA	Y
gc_52	Plasmid	TnpA tnpR	blaA	Y
gc_58	Plasmid	tnpR	blaA	Y
gc_59	Plasmid	IS66	sul1, qacE, aadA	N
gc_61	ICE	None	tetM	N
gc_80	ICE	None	tetM	N
gc_91	Plasmid	None	blaTEM, bla2	N
gc_92	Plasmid	None	sul2	N
gc_102	Plasmid	None	blaTEM	N
gc_107	ICE	None	ermB	N
gc_113	Plasmid	None	aadA, blaOXA	N
gc_120	Plasmid	SI91, res, intTn	sul2	Y
gc_133	Plasmid	None	aph3	N
gc_162	Plasmid	None	tetRC	N
gc_172	Plasmid	None	aph3, aph6	N
gc_181	Plasmid	None	tetA	N
gc_184	ICE	None	erm23	N
gc_188	Plasmid	None	tetG	N
gc_203	Plasmid	None	tetR, aph6	N
gc_216	Plasmid	None	tetX	N
gc_235	Plasmid	None	mph, mef	N
gc_242	Plasmid	None	qnrS	N
gc_257	Plasmid	None	lnuC	N
qc_156	ISCR	IS91	ermF	Y

Supplementary Table 3: Summary of the antibiotic susceptibility testing results.

Sample Site	E. coli CFU	Beta-lactams	Sulfanomides	Trimethoprim	Gentamicin	Tetracycline
ICC	33	0	0	0	0	0
DIB	53	0	0	0	0	0
BRA	55	0	0	0	0	0
BCT	74	1	0	0	0	0
BRA2	1020	0	0	0	0	0
EBR	500	1	0	0	0	0
KWW	350	0	0	0	0	0
KRB	150	0	0	0	0	0
MPB	65	0	0	0	0	0
MLB	6300	0	0	0	0	0
AHP	1200	0	0	0	0	0
SVP	1200	0	0	0	0	0
CWS	6400	0	0	0	0	0
CWN	1600	8	0	0	0	0
FCP	580	0	0	0	0	0
FRP	620	0	0	0	0	1
ILP	460	1	0	0	0	0
TBP	420	0	0	0	0	0
FSP	650	3	0	0	0	0
LDP	320	0	0	0	0	0
ICG	340	0	0	0	0	0
BBB	470	0	0	0	0	0
CPA	500	0	0	0	0	0
MWW	1300	0	0	0	0	0
UMC	2500	1	0	0	0	0

Supplementary Table 4: Characteristics of the four WWTPs associated with the study.

Wastewater Treatment Plant	Coordinates	Secondary Treatment *	Capacity		
			Flow Rate (MGD)	Load (P.E.)	Average Daily Flow (MGD)
Douglas L. Smith Middle Basin Plant (MBP)	38.9240271, -94.7022235	BNR	14.5	145,000	10.9
Tomahawk Wastewater Treatment Plant (TC)	38.9305624, -94.6247989	TF	4-7	40,000	6.5
Blue River Main (BRM)	38.8493673, -94.6190643	BNR	10.5	105,000	5.9
Blue River Wastewater Plant (BR)	39.1190071, -94.4976568	TF/GC	120	850,000	81 †

*AS=Activated Sludge; TF=Trickle Filter; TF/GC=Trickle Filter with Gravity Clarifiers
BNR=Biological Nutrient Removal
† data from 2011 operation permit

Supplementary Table 5: Description of sites sampled within the Blue River Watershed.

Site Code	Association	Latitude	Longitude	Field Site
ICC	WWTP	38.952958	-94.563948	Indian Creek
DIB	WWTP	38.956451	-94.560134	Blue River
BRA	WWTP	38.939143	-94.561442	Blue River
BCT	WWTP	38.903159	-94.578199	Blue River
BRA2	WWTP	38.939143	-94.561442	Blue River
EBR	WWTP	38.891412	-94.583257	Blue River
KWW	WWTP	38.854923	-94.615853	Blue River
KRB	Reference	38.842448	-94.612554	Blue River
MPB	Reference	38.813318	-94.670967	Blue River
MLB	Rural	38.818864	-94.778833	Coffee Creek
AHP	Reference	38.857594	-94.7882	Indian Creek South
SVP	WWTP	38.925804	-94.697055	Indian Creek South
CWS	Hospital	38.92075	-94.699529	Indian Creek North
CWN	WWTP	38.93288	-94.69652	Indian Creek
FCP	Reference	38.900837	-94.739828	Indian Creek South
FRP	Reference	38.942513	-94.737143	Indian Creek North
ILP	Reference	38.9002091	-94.6489961	Tomahawk Creek
TBP	Reference	38.922873	-94.625833	Tomahawk Creek
FSP	Reference	38.93085	-94.631627	Indian Creek
LDP	WWTP	38.932129	-94.61322	Indian Creek
ICG	WWTP	38.943062	-94.593613	Indian Creek
BBB	WWTP	39.017381	-94.521087	Blue River
CPA	Hospital	39.087112	-94.49946	Blue River
MWW	WWTP	39.119097	-94.489562	Blue River
UMC	DM *	39.119258	-94.476065	Blue River
* Drug manufacturing plant				

Supplementary Table 6: Physical and chemical parameters of sample site surface waters.

Site Code	DO (%)	SPC (uS/cm)	TDS (mg/L)	pH	ORP (mV)	Temperature (C)	Turbidity (NTv)
DIB	47.4	869	565.5	8.57	124.3	NA	2.95
BRA	52.7	690	448.5	8.62	67.9	NA	3.61
BCT	68.1	667	435.5	8.92	52.2	NA	1.29
EBR	69.4	648	422.5	8.77	130.8	28.4	1.74
KWW	60.8	504	325	8.07	143.9	25.9	1.37
KRB	69.2	600	390	8.36	96.6	27.4	5.19
MPB	59.4	520	338	8.65	121.3	26.8	7.16
BBB	59.2	505	331.5	8.18	124.3	33.4	7.22
CPA	69.2	634	409.5	8.42	108.4	29.2	8.86
MWW	59.6	633	409.5	8.45	131.3	25.9	10.94
UMC	54.2	661	429	8.35	130.1	28.2	20.72
MLB	57.3	404.6	263.25	8.26	146.4	22.8	69.33
ICC	22.4	1063	689	8.52	126.1	NA	7.47
AHP	65.3	291.5	189.8	8.91	126.4	28.6	1.69
SVP	64.6	715	468	8.33	154.1	26.5	3.48
CWS	65	436.6	280	8.32	68.5	30.7	15.05
CWN	69.7	698	455	8.34	95.2	27.7	6.38
FCP	68.3	439.6	286	8.34	97.7	25.9	2.15
FRP	64.2	531	344.5	8.43	113.7	24.1	1.01
FSP	62.1	653	422.5	8.36	154.5	25	8.27
LDP	67.3	395.5	256.1	8.27	150.4	27.7	7.51
ICG	62.9	681	442	8.22	139.7	29.8	4.9
ILP	57.1	661	429	8.54	136.4	23.3	2.03
TBP	69.1	565	364	8.44	150	24.8	4.69

Supplementary Table 7: Sequencing and assembly statistics.

Site Code	Sample ID	Raw reads *	QC reads *	Assembled reads *	Assembly size †	Longest contig †	N50 †	Predicted genes
ICC	0WARd141	214.68	213.74	191.75	1773029.807	790.281	1.39	2805270
ICC	0WARd143	165.12	164.87	147.82	1773029.807	790.281	1.39	2715470
ICC	0WARd145	160.1	158.96	143.99	1773029.807	790.281	1.39	2649963
DIB	0WARd147	164.51	163.47	140.17	2066497.786	886.934	1.31	3088504
DIB	0WARd149	143	142.55	125.55	2066497.786	886.934	1.31	2784405
DIB	0WARd151	132.48	131.7	103.25	2066497.786	886.934	1.31	2940173
BRA	0WARd153	198.65	197.21	163.08	1627030.767	222.045	1.192	2726818
BRA	0WARd155	156.08	154.84	133.58	1627030.767	222.045	1.192	2622683
BCT	0WARd159	216.97	215.08	190.12	1909046.573	243.513	1.153	3213441
BCT	0WARd161	140.26	139.41	124.54	1909046.573	243.513	1.153	3006743
BCT	0WARd163	141.75	141.51	127.27	1909046.573	243.513	1.153	2968453
BRA2	0WARd165	125.91	124.83	106.54	1356668.341	368.517	1.247	2048307
BRA2	0WARd167	85.28	84.49	72.85	1356668.341	368.517	1.247	1947600
BRA2	0WARd169	137.17	136.98	116.25	1356668.341	368.517	1.247	2147761
EBR	0WARd171	89.01	88.48	71.93	1707184.766	694.519	1.143	2473162
EBR	0WARd175	134.65	133.64	116.78	1707184.766	694.519	1.143	2652366
EBR	0WARd177	183.56	182.5	156.22	1707184.766	694.519	1.143	2831697
KWW	0WARd179	175.22	174.73	155.8	1185504.272	1058.463	1.381	1865421
KWW	0WARd181	160.66	159.41	143.18	1185504.272	1058.463	1.381	1770378
KWW	0WARd183	135.67	134.86	119.73	1185504.272	1058.463	1.381	1761288
KRB	0WARd185	173.6	171.44	124.76	2226793.91	380.215	1.293	3349704
KRB	0WARd187	117.16	115.99	84.86	2226793.91	380.215	1.293	3130327
KRB	0WARd189	123.18	122.57	73.64	2226793.91	380.215	1.293	3148582
MPB	0WARd191	94.53	93.78	75.41	1541349.776	552.934	1.479	1937485
MPB	0WARd193	126.38	125.36	99.65	1541349.776	552.934	1.479	2039725
MPB	0WARd195	200.26	200.12	158.15	1541349.776	552.934	1.479	2422736
MLB	0WARd197	158.21	156.83	65.36	1790892.087	641.849	0.958	2668606
MLB	0WARd199	171.9	171.76	64.16	1790892.087	641.849	0.958	2718086
MLB	0WARd201	89.17	87.47	35.36	1790892.087	641.849	0.958	2273581
AHP	0WARd203	122.41	122.02	41.07	2083048.327	874.703	0.882	3506581

AHP	0WARd205	140.5	139.32	49.2	2083048.327	874.703	0.882	3709573
AHP	0WARd207	123.65	123.39	43.32	2083048.327	874.703	0.882	3553086
SVP	0WARd209	181.31	180.23	132.19	1952078.806	601.753	1.287	3075726
SVP	0WARd211	176.91	175.9	132.3	1952078.806	601.753	1.287	3106407
SVP	0WARd213	130.34	129.09	94.56	1952078.806	601.753	1.287	2846919
CWS	0WARd215	180.93	180.68	84.05	1983953.61	324.478	0.924	3232833
CWS	0WARd217	153.62	152.08	74.68	1983953.61	324.478	0.924	2986842
CWS	0WARd219	153.9	153.48	77.25	1983953.61	324.478	0.924	3028946
CWN	0WARd221	156.23	154.97	102.81	1788982.017	1023.981	1.181	2837817
CWN	0WARd223	118.45	115.95	77.01	1788982.017	1023.981	1.181	2656357
CWN	0WARd225	143.89	141.32	97.53	1788982.017	1023.981	1.181	2737209
FCP	0WARd227	143.14	141.4	105.69	1317236.377	785.472	1.294	1937184
FCP	0WARd229	155.94	150.27	100.68	1317236.377	785.472	1.294	2008218
FCP	0WARd231	88.16	87.01	61.12	1317236.377	785.472	1.294	1817423
FRP	0WARd233	107.24	106.83	57.8	1093447.802	778.023	1.046	1438672
FRP	0WARd235	145.11	144.53	77.28	1093447.802	778.023	1.046	1542757
FRP	0WARd237	102.09	101.34	47.86	1093447.802	778.023	1.046	1437178
ILP	0WARd239	144.86	143.86	107.42	1991509.103	679.187	1.353	3008241
ILP	0WARd241	155.22	154.72	108.64	1991509.103	679.187	1.353	3101276
ILP	0WARd243	107.28	106.39	77.91	1991509.103	679.187	1.353	2870618
TBP	0WARd245	128.8	128.66	88.01	1825724.526	559.277	1.397	2721418
TBP	0WARd247	122.24	119.69	87.71	1825724.526	559.277	1.397	2610847
TBP	0WARd249	119.6	118.41	84.09	1825724.526	559.277	1.397	2648541
FSP	0WARd251	135.61	134.75	97.31	1421333.367	480.591	1.351	2125328
FSP	0WARd253	100.77	98.61	65.4	1421333.367	480.591	1.351	2019532
FSP	0WARd255	129.5	129.32	99.07	1421333.367	480.591	1.351	2111103
LDP	0WARd257	130.9	130.28	97.85	1858935.237	1046.936	1.17	2690783
LDP	0WARd259	149.2	149.04	111.6	1858935.237	1046.936	1.17	2880390
LDP	0WARd261	184.26	182.84	132.64	1858935.237	1046.936	1.17	2980981
ICG	0WARd263	203.02	202.11	139.94	2249366.77	506.848	1.131	3601002
ICG	0WARd265	85.41	83.53	60.09	2249366.77	506.848	1.131	2876908
ICG	0WARd267	179.38	177.54	131.17	2249366.77	506.848	1.131	3550160
BBB	0WARd269	114.04	112.27	83.86	1942089.993	318.27	1.202	2926643
BBB	0WARd271	166.58	164.96	126.41	1942089.993	318.27	1.202	3081325

BBB	0WARd273	114.47	114.09	87.02	1942089.993	318.27	1.202	2859632
CPA	0WARd275	222.68	219.05	145.65	2757479.071	458.349	1.038	4448272
CPA	0WARd277	126.58	126.17	77.36	2757479.071	458.349	1.038	3831727
CPA	0WARd279	145.71	144.71	111.74	2757479.071	458.349	1.038	3510064
MWW	0WARd281	162.49	160.64	128.17	2223033.81	325.827	1.224	3479168
MWW	0WARd283	157.29	156.73	125.54	2223033.81	325.827	1.224	3432362
MWW	0WARd285	145.1	144.24	117.73	2223033.81	325.827	1.224	3429777
UMC	0WARd287	173.4	173.25	91.91	2297663.705	361.295	0.979	3517234
UMC	0WARd289	98.42	98.21	62	2297663.705	361.295	0.979	2834563
UMC	0WARd291	153.57	153.15	108	2297663.705	361.295	0.979	3332918

* per million
† thousand bps (kbp)

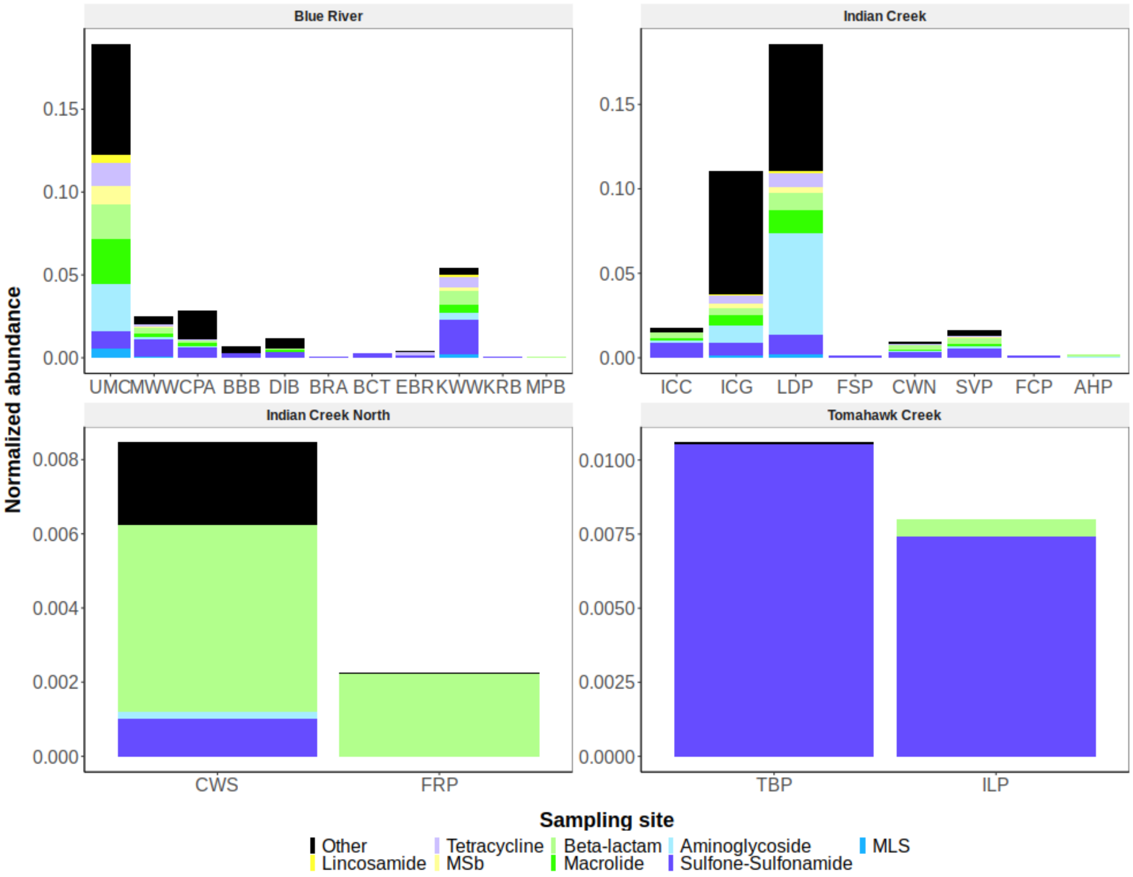

Supplementary Figure 1: Total normalized ARG abundance by sampling site. ARG abundances were normalized to the abundance of the rpoB gene by dividing the FP of each ARG to the FP of the rpoB gene. The Other category represents ARGs that together compose less than 5% of the total watershed resistome. Note that site DIB is the first site sampled downstream of the confluence of the Blue River and Indian Creek.

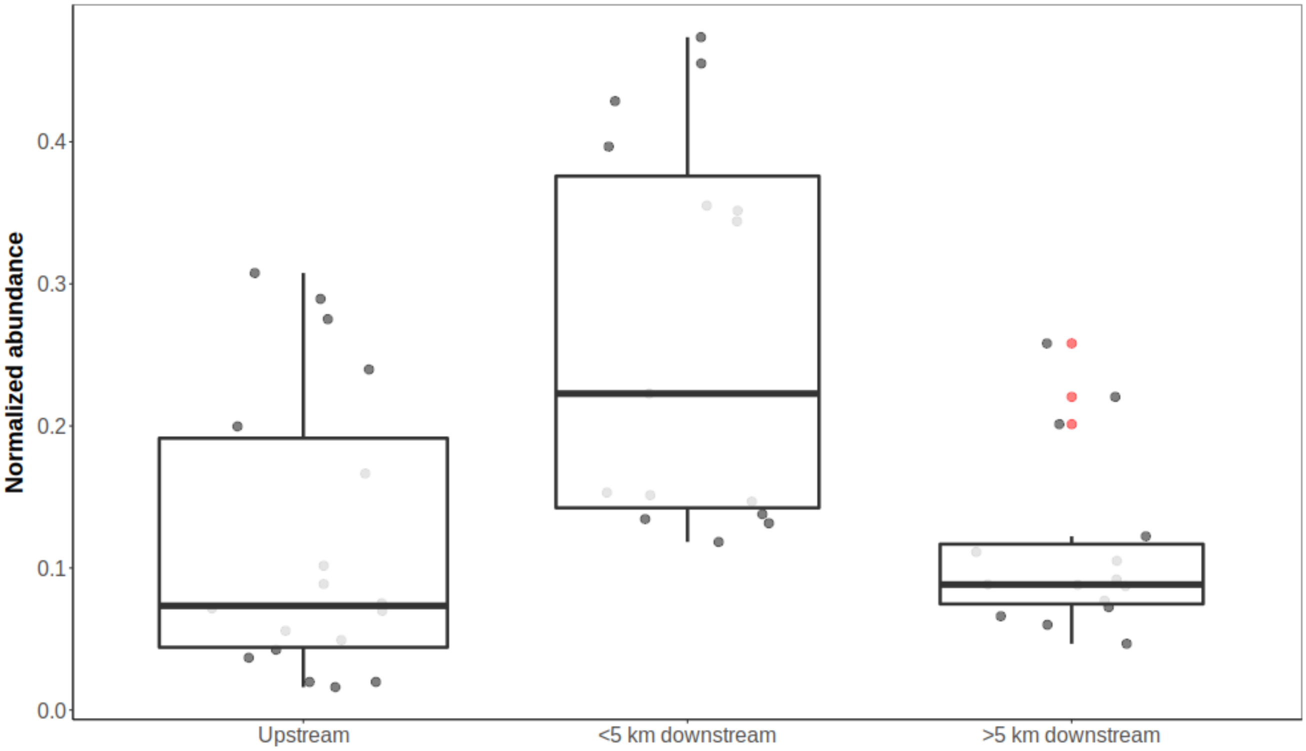

Supplementary Figure 2: Total ARG abundance at varying distances from WWTP. ARG abundances were normalized to the abundance of the rpoB gene by dividing the FP of each ARG to the FP of the rpoB gene, and then root-transformed for analysis. Samples are grouped based on where they were collected relative to the nearest WWTP (Upstream = surface waters with no impact from a WWTP; <5 km downstream = within 5 km downstream the nearest WWTP; >5 km downstream = more than 5 km downstream the nearest WWTP). The area between the lower and upper hinge represents the inter-quartile range (IQR), or difference between the first and third quartiles.

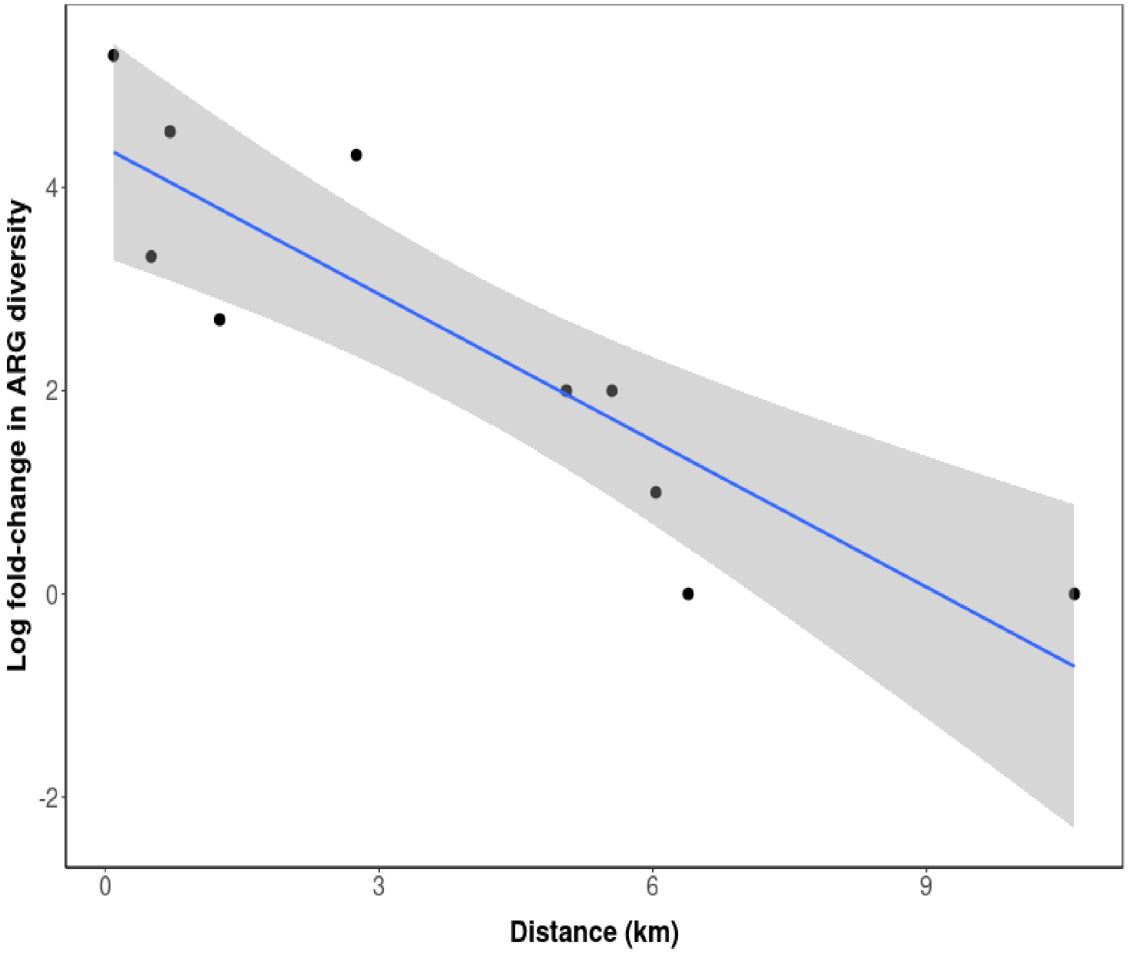

Supplementary Figure 3: ARG richness with increasing distance downstream a WWTP. The change in ARG richness was calculated as the log2 fold-change in the number of unique ARG types detected between a downstream WWTP site and its closest upstream site. A smoothing curve based on linear regression (blue line) is shown along with 95% confidence intervals (shaded region).

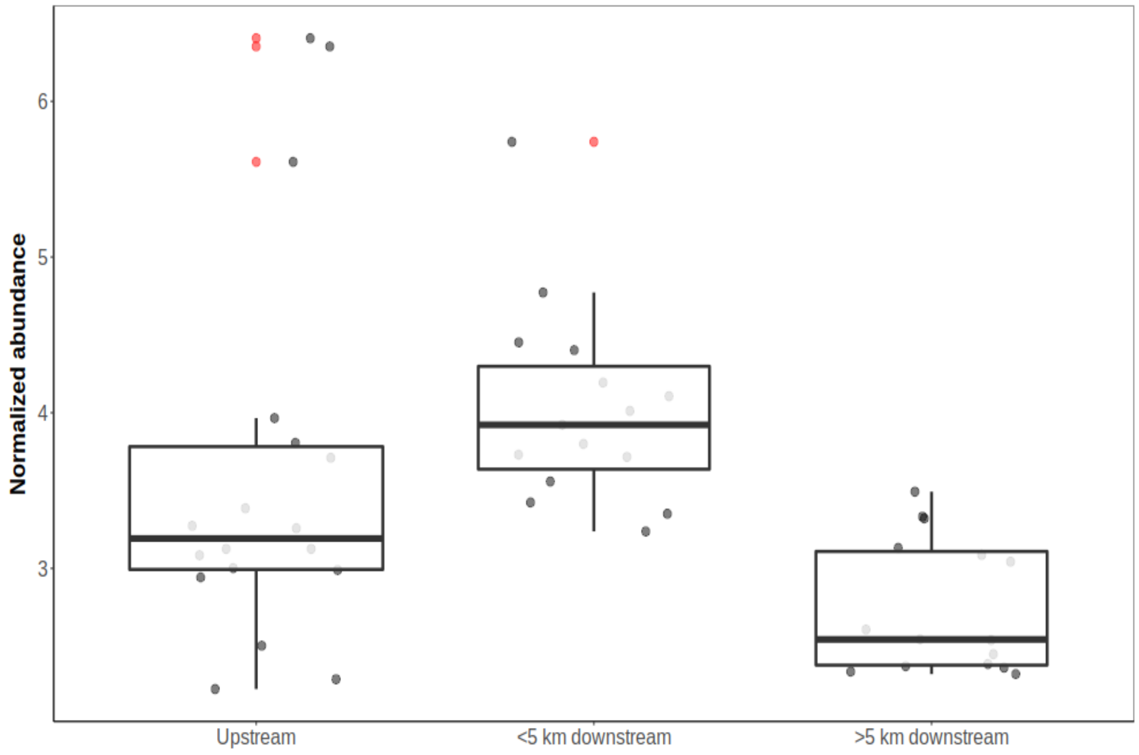

Supplementary Figure 4: Total MGE abundance at varying distances from WWTP. MGE abundances were normalized to the abundance of the rpoB gene by dividing the FP of each ARG to the FP of the rpoB gene, and then root-transformed for analysis. Samples are grouped based on where they were collected relative to the nearest WWTP (Upstream = surface waters with no impact from a WWTP; <5 km downstream = within 5 km downstream the nearest WWTP; >5 km downstream = more than 5 km downstream the nearest WWTP). The area between the lower and upper hinge represents the inter-quartile range (IQR), or difference between the first and third quartiles.

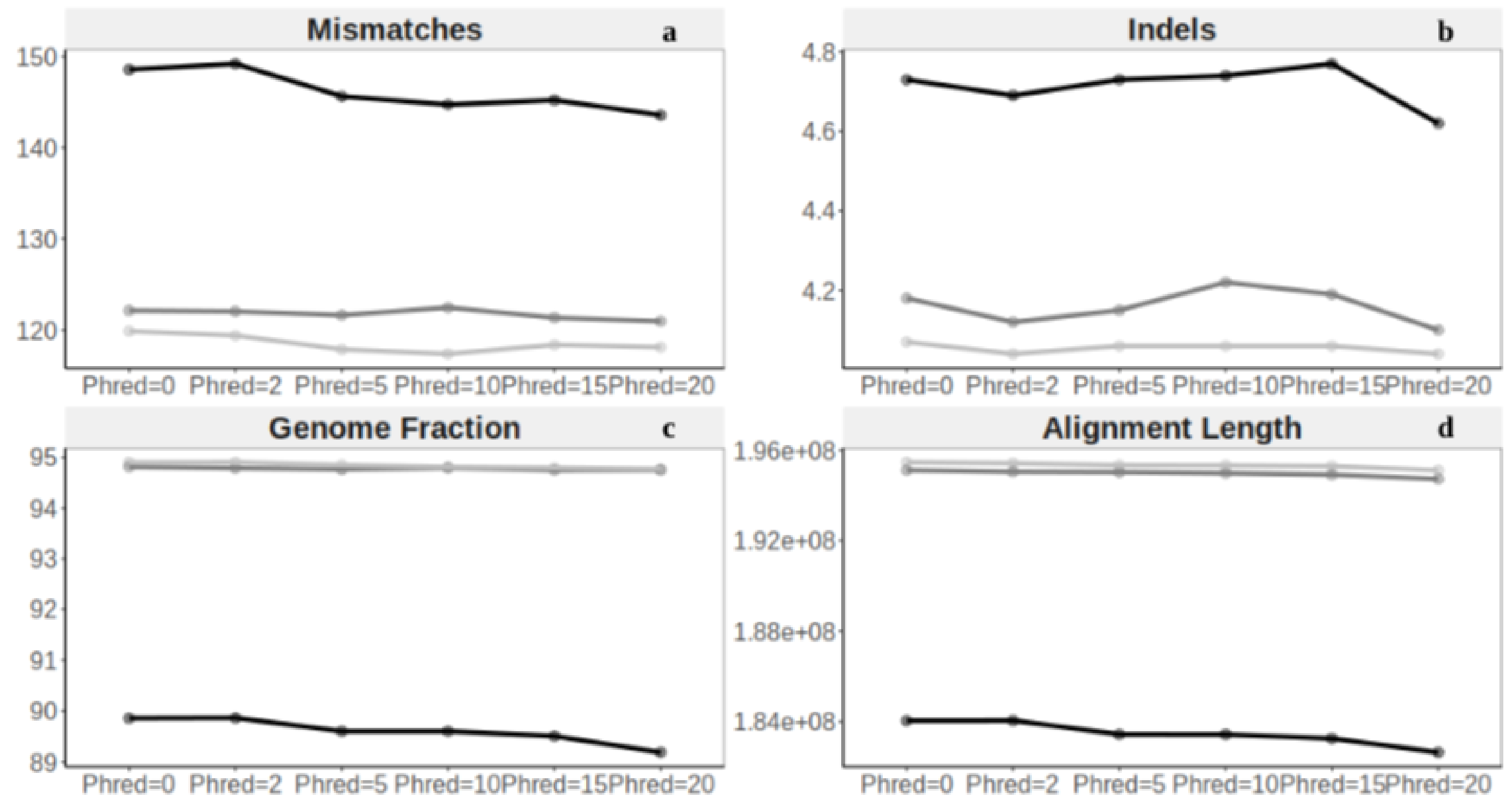

Supplementary Figure 5: Association between assembly quality and quality score threshold at varying sequencing depths. Top panels represent two of the most common types of assembly error – number of mismatches (a) and indels (b). Bottom panels represent standard metrics of assembly performance – fraction of the genome covered by reads (c) and total alignment length (d).
