# Supplementary material for: Localized effect of treated wastewater effluent on the resistome of an urban watershed
Source: Gigascience. 2020 Nov 19;9(11):giaa125. doi: 10.1093/gigascience/giaa125 (PMC7677451; doi:10.1093/gigascience/giaa125)
Supplement: giaa125_Response_to_Reviewer_Comments_Original_Submission [file giaa125_response_to_reviewer_comments_original_submission.pdf]

Author's Response To Reviewer Comments

Close

Dear GigaScience Editorial Office,

We thank the reviewers for their helpful comments and suggestions. We have addressed all comments in the revised manuscript, and we believe that the revised manuscript is substantially improved.

In addition, the python package developed for the project has been registered in the bio.tools and SciCrunch.org databases, and the identifiers are listed in the revised manuscript.

Reviewer #1 (Comments for the Author):
The authors present a comprehensive study on the effect of waste water effluent release on river resistome. The manuscript is well written and the results clearly presented. The conclusion are supported by the results. It also includes all necessary information required by the journal.
Of course, there are still some parts that could be improved and I hope my comments would be useful. Detailed point-by-point comments can be found below. In many places whole sentences are copied, because the manuscript lacks line numbers.

1. I'll start with the title. This is more of an opinion, but I would like to see a title that tells something about the results. And to be precise, waste water treatment is not what's actually influencing the urban watershed, but the effluent release.

Response:
We appreciate the suggestion and have revised the title accordingly: "Localized effect of treated wastewater effluent on the resistome of an urban watershed".

2. First sentence of the abstract could be rephrased. Since WWTPs are doing pretty good job in removing ARGs and ARBs from sewage, would be nice to bring this up already here. Now it gives impression that WWTPs are the problem. Without them, raw sewage would be released directly to environment.

Response:
Our intent was not to suggest that raw sewage is preferable to wastewater treatment. However, we agree that the abstract, in particular, should not be seen to target wastewater treatment plants. We have rephrased the sentence accordingly: "Wastewater treatment is an essential tool for maintaining water quality in urban environments. While the treatment of wastewater can remove most bacterial cells, some will inevitably survive treatment to be released into natural environments."

3. Sentence in abstract: "Treated effluent was found to be associated with increased abundance and diversity of antibiotic resistance genes and mobile genetic elements".

Since you didn't sample effluents, I think this should be: "Treated effluent release was found...".

Response:
The sentence was changed as suggested.

4. Fourth paragraph of Background: "In addition, selection for resistance can occur during water treatment even at subinhibitory levels of antimicrobial compounds."

This work and all the others are done in simple settings and the work done with more complex communities has shown that selection occurs at much higher concentrations. These studies are more relevant to this study and should be referenced.

Response:
We agree that selection at subinhibitory levels is unnecessary to mention here. We have revised that

paragraph accordingly:
"The localization of ARGs on mobile genetic elements (MGEs), such as transposons and plasmids, enables their movement between bacterial cells of the same or different species. Transfer of resistance factors is likely to increase during exposure to selection factors such as antibiotics and other environmental pollutants [15]. The conditions in WWTPs, including the mixture of organisms from diverse environmental origins and the availability of surfaces and biofilms, can create strong selection pressures for resistance [16, 7]. Even those treatments that are effective in removing bacteria from the water can promote the exchange, selection, and dispersal of AR genes [17, 18]."

5. "Although previous studies have investigated antimicrobial resistance in WWTPs and treated effluent, few studies have addressed the impact of wastewater on the environmental resistome throughout an urban watershed. Until recently, such studies have been limited to the analysis of a few known resistance genes using molecular detection techniques such as qPCR or to the detection of a cultivable minority of resistant bacteria using culture-dependent techniques".

The authors have missed many studies about the influence of effluent release on receiving environments that have used metagenomics. This study might be more comprehensive than previous studies, but the main message is nothing new actually. Effluent contains ARGs that make up the resistome in receiving environments, but the effect dilutes with distance.

Response:
No metagenomic studies of a single watershed had been published while we were working on this project, but it is true that these studies are now coming out. We have revised the paragraph accordingly:
"Recently, metagenome sequencing of wastewater effluent has been implemented as a useful tool for monitoring the spread of ARGs into natural environments (e.g. [19, 20]). Few studies, however, have investigated the environmental resistome throughout a single urban watershed from its headwaters to a major drainage. In this study, we sequenced metagenomes from 72 river samples collected from an urbanized watershed with the goal of assessing the impact of point sources of human waste on the resistance profile of receiving river surface waters, focusing especially on ARGs associated with MGEs."

6. The normalisation used by the authors is problematic: "DNA base pairs per quantity of sampled material" (BPS-1)". Sequencing data is always compositional (see e.g. doi:10.3389/fmicb.2017.02224), so it cannot be normalised with the amount of starting material. And authors used "Whenever possible, 80 ng of purified DNA ".

Response:
We acknowledge the reviewer's point about the compositional nature of sequencing data. We believe that our BPS-1 metric could be justified as being consistent with compositional data, but we now realize that such a claim requires more evidence with an extensive statistical discussion that would be beyond the scope of this paper and would not substantively affect the results or conclusions of this paper. Therefore, we have removed the BPS-1 metric from the manuscript. The y-axis of Figures 1 and 2 remains as "normalized coverage," which is almost completely unaffected by the choice of normalization procedure. To demonstrate this, we repeated all analyses with a normalization to rpoB, as recommended by Reviewer #2 (see below). The results are unaffected, and we provide figures reporting the rpoB-normalized abundances as additional data.

7. The statistical test used could be mentioned whenever results from statistical tests are presented.

Response:
We have added the appropriate statistical tests as suggested.

8. "No correlation was found between crAssphage and ARG abundances in samples collected from sites without an upstream WWTP (adjusted R2 = 0.0009, p = 0.32). This was unexpected given that one large-scale study had previously found significant associations between crAssphage and ARG abundance in environments both with and without influence from WWTPs".

I don't think that was our message. Lack of correlation means that the ARGs are originating from another source, e.g. animal farming. Figure 2 shows that in sites without WWTP, crAssphage abundance is very low compared to sites with WWTP. I would be more informative to have all the data in figure 2 in one plot.

Response:
The second sentence has been removed, and we added "is not due to a complete absence of human fecal pollution but suggests the influence of additional environmental factors that were not measured in this study" to the final sentence of this section. Also, we explored the option of combining all data points into a single figure but maintain that splitting them into two figures is more informative. The first paragraph under "Statistical Analyses" provides justification for separating the sites based on location relative to WWTPs. We have also added "Note the smaller range of crAssphage abundances in samples without upstream WWTPs." to the figure caption.

9. "slightly but not significantly higher ($p = 0.09$)". This might be debatable, but I don't think this is correct way to present results. According to the statistical test, there was no difference.

Response:
We have rephrased the sentence to prevent potential misinterpretation.

10. The authors write that sulphonamide resistance gene, sul1, was found from multiple mobile context. However, it is not presented what mobile elements these were. I would guess all are class 1 intergrons. So sul1 was always found from class 1 integrons, as can be seen in figure 4, that shows high homology to class 1 integron backbone in P. aeruginosa. I wouldn't call these multiple mobile contexts.

Response:
Supplementary Table 2 shows sul1 in two separate genetic contexts. Therefore, we have revised the sentence to the following: "The sulfonamide resistance gene sul1 was found in two distinct mobile contexts throughout the watershed (Supplementary Table 2), but in upstream sites, it appeared primarily in an integron that also contained the quaternary ammonium compound (QAC) resistance genes qacE and qacG."

11. "Interestingly, this site is not downstream from a WWTP but is <3 km downstream from a regional hospital."

Is the hospital releasing something to the river? If not, this is irrelevant.

Response:
We agree that the sentence is irrelevant without further information about how the hospital treats its wastewater. It has been removed from the revised version.

12. "Antibiotic resistance genes (ARGs) have been previously detected in the discharge of wastewater treatment plants (WWTPs) [13, 14, 12], but previous studies have targeted specific resistance determinants or have sampled only one or a few locations."

This is related to comment #5, not fully true. Would be better to tell what you did and why its important than to try understate previous studies. At least state that it is known that ARGs can be found from downstream environments. The bigger question is "What then?"

Response:
We have rephrased the sentence to read, "Antibiotic resistance genes (ARGs) have been previously detected in the discharge of wastewater treatment plants (WWTPs) [25, 26, 11, 8], as well as in receiving aquatic environments such as rivers and lakes [19, 27, 13, 14. 28, 29, 12]. Here, we conducted an extensive metagenomic study to investigate the distance decay effect of four wastewater treatment plants on the resistance profile of an urban watershed."

13. "River samples collected downstream from WWTPs had significantly higher environmental abundance and diversity of ARGs, suggesting that WWTPs can be an important contributor of ARGs to receiving surface waters."

Again, related to previous. This is not so novel finding. And also WWTPs are not the real contributor, but human (or animal) fecal material and actually most WWTPs are part of the solution.

Response:
We have revised the sentence to focus on the distance decay effect, which has not been previously reported for the flowing waters of an urban river (as opposed to sediments):
"River samples collected immediately downstream from WWTPs had significantly higher environmental

abundance and diversity of ARGs, and this effect diminished in samples collected >5 km downstream. These results provide additional support for a localized effect of WWTPs on the resistome of receiving aquatic environments."

14. "Wastewater treatment can result in substantially reduced total bacterial load, but cells that survive the treatment process are more likely to be antibiotic-resistant (see Karkman et al. 2018 for review)"

I don't think we say this in our review. Due to the high bacterial loads, biofilms and antibiotics found from sewage, WWTPs could be hotspots, but there isn't really any evidence of that. But you can prove me wrong in here, of course. And naturally, I appreciate that you cite our work, and would kindly ask you to add this also to the reference list, now it's only in the text.

Response:
We have corrected the mismatches between citations and works cited list, which was due to a software error. We have also removed the comment about the surviving cells being more likely to be resistance, as it was irrelevant to the point of this paragraph, which we have attempted to clarify with a few minor wording changes.

15. "In the two largest rivers investigated here, Blue River and Indian Creek, WWTP effluent could comprise over 95 percent of base flow under typical operating conditions [27]"

By reading the reference, I got the impression that it's on average 15 %, but maybe I got something wrong.

Response:
The reviewer is correct. According to the reference that we cited, during the period investigated in the USGS study WWTPs contributed an average of 15% of base flow in the Blue River, which approached 50% during the dry season. In some instances, however, WWTPs were observed to contribute greater than 95% of stream flow. This information was mistakenly attributed to the report cited, but was actually obtained from a later report on the same study (Wilkison et al. 2006), which we have since cited. We realize that the way the sentence was phrased could be confusing, however, and have revised the sentence accordingly: "In the two largest rivers investigated here, Blue River and Indian Creek, WWTPs contribute on average 15% of base flow [33], and could contribute over 95% under certain conditions [34]."

16. "These results, combined with the strong correlation of total ARG abundance with the abundance of a human gut phage, is consistent with the interpretation that ARGs were released into surface waters with human fecal pollution and then diluted in downstream waters"

This has also been shown in other studies, so it would be appropriate to cite these studies here.

Response:
We have added citations to this sentence.

17. "This same mobile element was ubiquitous throughout the entire watershed, spanning multiple streamflows, land use types, and pollution levels, suggesting that it may be maintained in natural microbial communities due to a variety of selection pressures"

Could there be another source of ARGs in these upstream sites? Agriculture/animal farming? The numbers in figure 2 are pretty high for non-WWTP sites as well. So it's not probably just environmental background. Or is this an artefact from the normalization?

Response:
The particular mobile element mentioned in the paragraph was not found exclusively in upstream sites, but throughout the entire watershed. The abundance distribution in non-WWTP sites is nearly identical with other normalization choices (see our new additional data with rpoB-normalized abundances and responses to Reviewer 2 below). The watershed was mostly urbanized, with some agriculture near the sampling sites closest to the headwaters. It is highly unlikely that agriculture, and animal agriculture in particular, could explain the presence of these ARGs throughout the river waters considering the rarity of farming in the region. While we can't rule out the possibility that some other consistent pollution source could be contributing ARGs to the river surface waters, this scenario seems unlikely given the extent of the watershed and variability in land use. Nevertheless, we have revised the paragraph to acknowledge

the possible influence of pollution near the headwaters:

"The abundance of ARGs in sites upstream from all WWTPs, in contrast, were independent of human fecal pollution levels. The relatively high abundances of ARGs in these sites suggests the influence of agricultural pollution near the headwaters, but this hypothesis was not tested by the current study. The most abundant ARG in upstream samples was the sul1 sulfonamide resistance gene, which was primarily encoded on a novel class 1 integron along with two QAC resistance genes. This same mobile element was ubiquitous throughout the entire watershed, spanning multiple streamflows, land use types, and pollution levels, suggesting that it may be maintained in natural microbial communities due to a variety of selection pressures."

18. In methods, the authors write: "This smaller size fraction was the focus of the study, as it was considered to be more representative of suspended cells that are dispersing through the watershed rather than being retained in sediments and debris."

Does this mean that 5 µm filters were not included in this study? It should be discussed on how this might affect the results. Not all bacteria in environmental samples (flocks/biofilms/cell aggregates) will pass 5 µm filter.

Response:
Size fractionation is a common, though contentious, strategy in aquatic ecology. There are no universally accepted procedures across all types of aquatic systems. We added another sentence to this paragraph that discusses our rationale for focusing on the small size fraction:
"No sequencing was performed with the 5 µm filters; therefore, genes encoded by large cells or cells associated with aggregates and biofilms are likely to be under-represented in our study."

19. The approach to use only assembled data has its limitations as the authors do point out. So I would have liked to see read-based analysis done as well. It would have given a better view of the resistome. But the bias is the same for all samples, so the overall message of the manuscript probably won't change.

Response:
We agree that it would be interesting to see a comparison of the different methods used in environmental resistance profiling. Unfortunately, such a comparison was beyond the scope of our study.

20. The assemblies could be provided. Assembling all the data to verify the results is bit too much for review purposes. Also, more information about the assemblies could be adde to suppl. table 7: size of assembly, longest contig, mapping rates.

Response:
The assemblies have been uploaded to NCBI WGS under the BioProject accession listed in "Availability of supporting data and materials". Additional information about the assemblies has also been added to the supplementary table as suggested.

21. The authors have samples 24 sites in triplicates and end up with 74 metagenomes. I don't get the math.

Response:
Only 72 metagenomes were used in the analysis. One site was sampled on two separate days to determine the effect that the previous night's heavy weather event might have on the microbial community. Once the data was in hand, we verified that the samples collected from the second time point were more similar to the samples from the first time point than to any of the other samples collected throughout the watershed, which was sufficient to say that the impact was minimal in the context of the study. For unrelated reasons, we were successful in extracting DNA from only two samples from the first time point, so the samples collected from the second time point were used in the analyses. We have changed the number of metagenomes to 72 throughout the manuscript to avoid confusing the reader. However, 74 datasets, including data from both time points, have been submitted to the SRA.

22. I don't understand the discussion about heteroresiostance affecting assemblies. I think bigger problem in trying to assemble antibiotic resistance genes is their location in multiple genetic context.

The assembler can't solve these and many times the assembly breaks outside the ARG.

Response:
We agree. Nevertheless, strain variation is also a challenge to assemblers, and because heteroresistance necessarily adds to strain variation, we thought that it could be an interesting avenue of research to explore in the future, and therefore worth mentioning. We agree, however, that as written, it is a distraction and not necessary to include in the methods. We have removed these sentences.

23. Related to the previous comment, the authors have used software called Mix for merging contigs. I don't have experience with it, but as it is designed for WGS of single isolates, I would be very cautious when using it with metagenomes. It might produce chimeric sequences, especially with highly homologous ARGs and MGEs. Mapping reads back to these merged contigs could give some overview of it.

Response:
We agree with the reviewer that one should be cautious when using an assembly reconciliation tool designed for genomes on metagenomics data. What we failed to mention, however, was that the individual contigs were mapped back to the merged contigs and only the merged contigs that passed manual inspection for misassembly were considered. This omission has been rectified in the text.

Reviewer #2 (Comments for the Author):
The manuscript by Thornton et al. describes a rather large metagenomic study of antibiotic resistance genes (ARGs) in a US watershed. The authors analyze water microbial communities at 24 different sites with differing impact of human activities (mostly in the form of treated WWTP effluent). The methodology and reasoning are overall well thought through and sound, as are the conclusions. While the outcomes of the study are not that spectacularly new - almost all of the findings were pretty much known since before, but in a much more bits-and-pieces way - I like how this study ties all these pieces together and aligns multiple views on resistance dissemination in the environment.

The paper is well written and overall easy to read and addresses an interesting subject. I only have one slightly bigger complaint. As the authors are specifically looking for bacterial resistance genes, I think it would make more sense to normalize to e.g. total Bacterial 16S rRNA or rpoB or similar, as most people studying resistance in the environment do. Instead, the authors invent a new measure of resistance abundance that is hard to understand and compare to anything else. This seems unnecessary and makes the paper hard to compare to existing literature. Otherwise, I think this work is good.

I would recommend the paper for publication after addressing some minor comments and making some clarifications (see below).

1. Page 2, last paragraph: How was ARG diversity measured?

Response:
ARG diversity was measured as the number of unique ARG types detected at a site (or within the watershed, depending on context). This has been defined in the methods.

2. Table 1: I don't understand the units in this table. How is abundance measured? What would an abundance of 1 mean? What is the detection limit? What would 100% resistome correspond to? How is prevalence counted, given that there were only 24 sites? This needs to be much better clarified.

Response:
We understand that our abundance measure is not very informative in this context. It has been replaced with average length-normalized copies per rpoB gene, which will allow for comparison with similar studies. We have also updated the table for clarity as suggested and have defined "percent resistome" in the methods.

3. Page 5, last paragraph before "Potential implications": 1) What Karkman paper is referred to here? I can't find it in the reference list. 2) I am not so sure it is true that WWTPs select for resistant bacteria. The data I have seen suggests no selection for resistance in municipal WWTPs (equally resistant bacteria

come in and go out, but in much smaller numbers going out). I think this statement overstates the evidence in one direction.

Response:
Please see response to Reviewer 1, comment #14.

4. Page 6, first paragraph: You can do assembly AND also map the individual genes to an ARG database! You don't have to choose one strategy!

Response:
Please see response to Reviewer 1, comment #19.

5. Page 6, first paragraph under "Sample collection": How does placing the tubing upstream of the sampling equipment minimize contamination?

Response:
Placement of the tubing upstream of sampling equipment would prevent contaminants shed from the sampling equipment and personnel (perhaps including those collected from other sampling locations) from being pumped into the filter, as the equipment and personnel would be located downstream of the water intake. This is standard protocol in aquatic ecology. However, the phrasing could be improved. We have changed the sentence to read, "... to reduce unnecessary exposure to contaminants from sampling equipment and personnel, and ...".

6. Page 8, second paragraph under "Sequence assembly": What measures were investigated to asses assembly performance? That is pretty important to gauge the relevance of this assertion.

Response:
There were four primary metrics used in the comparison of the assemblies at different score thresholds: the total number of mismatches and indels, the total aligned length, and the fraction of the reference metagenome covered by the assembly. As the composition of the reference was known, we deemed these metrics to me more relevant than some of the more common metrics used to measure assembly quality, such as N50 score and read mapping rates. The four metrics used are not directly mentioned in the text, but are all shown in Supplementary Figure 5.

7. Page 8, first paragraph under "Identification and quantification of ARGs and MGEs": What settings were used for AMRFInder? How was a "true" (high-confidence) resistance gene hit defined?

Response:
AMRFinder was used with the default settings. This has been clarified in the relevant methods section.

A "true" resistance gene hit was defined (in AMRFinder terminology) as either an allele, which is found by searching query AA sequences against a database of AA sequences with BLASTP and a high sequence similarity threshold, or an exception, which are HMM models created from groups of nearly identical sequences (e.g. ANT(3'')-Ia family aminoglycoside nucleotidyltransferase AadA1). Above the level of exception are the 'equivalog' and 'subfamily' types, which are built from groups of proteins with various levels of homology. A comparison of these models with the CARD database (v 2.0.1) suggested that either some resistance elements are underrepresented in CARD (particularly aminoglycoside resistance genes and beta-lactamases) or that the seed sequences used in creation of some AMRFinder equivalog and subfamily HMM profiles could be too divergent, which would render their profiles overly sensitive at the expense of specificity. As we have no way of knowing the true reason, we decided to exclude the results of those models to ensure that only the most confident results were reported.

8. Page 9, third full paragraph: I don't get why the last part of the FP calculation is done. Do you assume that you have obtained all possible features from every sample in the assembly? That's an assumption very unlikely to be true. What is the aim of this step and what assumptions is it operating under?

Response:
The calculation does not assume that every possible feature has been identified from the samples, and does not rely on it either. Features here simply mean predicted protein genes, and the assumption is that all members of the watershed bacterial community encode genes in a way that is detectable by the detection method used (prodigal), which is a reasonable assumption to make. The purpose of calculating

FP is to convert our mapping rates into a measure of relative abundance (see comment #6 from Reviewer #1 for the reason). It is related to the TPM abundance measure for RNA-Seq expression estimation, which we modified to be more relevant to metagenomics feature abundance estimation, and has identical assumptions. TPM was originally described here (https://doi.org/10.1093/bioinformatics/btp692).

Close
