# Supplementary material for: Localized effect of treated wastewater effluent on the resistome of an urban watershed
Source: Gigascience. 2020 Nov 19;9(11):giaa125. doi: 10.1093/gigascience/giaa125 (PMC7677451; doi:10.1093/gigascience/giaa125)
Supplement: giaa125_Response_to_Reviewer_Comments_Revision_1 [file giaa125_response_to_reviewer_comments_revision_1.pdf]

Author's Response To Reviewer Comments

Close

Dear GigaScience Editorial Office,
We appreciate the reviewers' continued efforts to improve the paper, and believe that we have now adequately addressed their final concerns.

Reviewer #1 (Comments for the Author):
The authors have revised the manuscript and replied to all concerns raised by the reviewers.

1. The manuscript has improved, but I still have one concern about the normalisation that I raised already on the first round of review (point #6). As reviewer #2 also raised the same issue, I think it should be corrected in all figures and analyses. Now the authors have only removed the description of normalisation from the methods, but kept it in figures and analyses.

Response:
We have updated all of the relevant figures, tables, and statistical test results to reflect the new normalization requested by the reviewers. Previously these results were reported in the supplemental material, and they are now reflected in the main text, as requested.

Reviewer #2 (Comments for the Author):
The authors have addressed almost all my concerns in their revised manuscript. However, there are two instances where I think they could still improve the clarity of the manuscript and where I feel that they skirted my comments a bit.

1. In Table 1, it is still not clear how prevalence is counted, given that there were only 24 sites. How can the prevalence then be >24?

Response:
The numbers in the table reflect the prevalence on a per sample basis, as three samples were collected at each sampling site. However, we realize that it would be informative to also provide the site prevalence, so we have updated the table to include those numbers as well.

2. Under "Identification and quantification of ARGs and MGEs", exactly what was regarded a "high sequence similarity threshold" (see my earlier comments)? It should not be hard to provide this number.

Response:
The revised manuscript states:
"Homologs of known antibiotic resistance determinants were identified from the predicted protein sequences using AMRFinder v1.04 with default settings [21]. ARGs detected through AMRFinder with family type equal to "equivalog" or "subfamily" were excluded to ensure that only the highest confidence results were reported."

There is no single answer to the reviewer's question because AMRFinder uses a tiered decision-making process, as explained in the AMRFinder documentation:
BLASTP is run with the -evalue 1e-20 -comp_based_stats 0 options against the AMR gene database described above. Exact BLAST matches over the full length of the reference protein are reported. If there is no exact match, then the following rules are applied unless overridden by a curated blast cutoff: Matches with < 90% identity or with < 50% coverage of the protein are dropped. If the hit is to a fusion protein than at least 90% of the protein must be covered. Curated blast cutoffs override these defaults. A BLAST match to a reference protein is removed if it is covered by another BLAST match which has more identical residues or the same number of identical residues, but to a longer reference protein. A single match is chosen as the best of what remains sorting by the following criteria in order (1) if it is exact; (2) has more identical residues; (3) hits a shorter protein; or (4) the gene symbol comes first in alphabetical order.

Therefore, we have added a sentence referring the reader to the AMRFinder documentation if the reader would like to know more about how ARGs are detected by AMRFinder.

Close
