# Supplementary material for: Localized effect of treated wastewater effluent on the resistome of an urban watershed
Source: Gigascience. 2020 Nov 19;9(11):giaa125. doi: 10.1093/gigascience/giaa125 (PMC7677451; doi:10.1093/gigascience/giaa125)
Supplement: giaa125_Response_to_Reviewer_Comments_Revision_2 [file giaa125_response_to_reviewer_comments_revision_2.pdf]

Author's Response To Reviewer Comments

Close

Editor comments:
1. The protocols.io DOI for the DNA extraction method needs to be cited in the paper, as well as the GigaDB DOI.

The citations have been added in the references section and main text, as directed in the PDF comments.

Close
