# Supplementary material for: Localized effect of treated wastewater effluent on the resistome of an urban watershed
Source: Gigascience. 2020 Nov 19;9(11):giaa125. doi: 10.1093/gigascience/giaa125 (PMC7677451; doi:10.1093/gigascience/giaa125)
Supplement: giaa125_Reviewer_1_Report_Original_Submission — Antti Karkman -- 5/7/2020 Reviewed [file giaa125_reviewer_1_report_original_submission.pdf]

Reviewer Report

Title: Localized effect of treated wastewater effluent on the resistome of an urban watershed

Version: Original Submission **Date:** 5/7/2020

Reviewer name: Antti Karkman

Reviewer Comments to Author:

The authors present a comprehensive study on the effect of waste water effluent release on river resistome. The manuscript is well written and the results clearly presented. The conclusion are supported by the results. It also includes all necessary information required by the journal.
Of course, there are still some parts that could be improved and I hope my comments would be useful.
Detailed point-by-point comments can be found below. In many places whole sentences are copied, because the manuscript lacks line numbers.
1. I'll start with the title. This is more of an opinion, but I would like to see a title that tells something about the results. And to be precise, waste water treatment is not what's actually influencing the urban watershed, but the effluent release.
2. First sentence of the abstract could be rephrased. Since WWTPs are doing pretty good job in removing ARGs and ARBs from sewage, would be nice to bring this up already here. Now it gives impression that WWTPs are the problem. Without them, raw sewage would be released directly to environment.
3. Sentence in abstract: "Treated effluent was found to be associated with increased abundance and diversity of antibiotic resistance genes and mobile genetic elements".
Since you didn't sample effluents, I think this should be: "Treated effluent release was found...".
4. Fourth paragraph of Background: "In addition, selection for resistance can occur during water treatment even at subinhibitory levels of antimicrobial compounds."
This work and all the others are done in simple settings and the work done with more complex communities has shown that selection occurs at much higher concentrations. These studies are more relevant to this study and should be referenced.
5. "Although previous studies have investigated antimicrobial resistance in WWTPs and treated effluent, few studies have addressed the impact of wastewater on the environmental resistome throughout an urban watershed. Until recently, such studies have been limited to the analysis of a few known resistance genes using molecular detection techniques such as qPCR or to the detection of a cultivable minority of resistant bacteria using culture-dependent techniques".
The authors have missed many studies about the influence of effluent release on receiving environments that have used metagenomics. This study might be more comprehensive than previous studies, but the main message is nothing new actually. Effluent contains ARGs that make up the resistome in receiving environments, but the effect dilutes with distance.
6. The normalisation used by the authors is problematic: "DNA base pairs per quantity of sampled material" (BPS-1)". Sequencing data is always compositional (see e.g. doi:10.3389/fmicb.2017.02224), so it cannot be normalised with the amount of starting material. And authors used "Whenever possible, 80

ng of purified DNA ".
7. The statistical test used could be mentioned whenever results from statistical tests are presented.
8. "No correlation was found between crAssphage and ARG abundances in samples collected from sites without an upstream WWTP (adjusted R2 = 0.0009, p = 0.32). This was unexpected given that one large-scale study had previously found significant associations between crAssphage and ARG abundance in environments both with and without influence from WWTPs".
I don't think that was our message. Lack of correlation means that the ARGs are originating from another source, e.g. animal farming. Figure 2 shows that in sites without WWTP, crAssphage abundance is very low compared to sites with WWTP. I would be more informative to have all the data in figure 2 in one plot.
9. "slightly but not significantly higher (p = 0.09)". This might be debatable, but I don't think this is correct way to present results. According to the statistical test, there was no difference.
10. The authors write that sulphonamide resistance gene, sul1, was found from multiple mobile context. However, it is not presented what mobile elements these were. I would guess all are class 1 intergrons. So sul1 was always found from class 1 integrons, as can be seen in figure 4, that shows high homology to class 1 integron backbone in P. aeruginosa. I wouldn't call these multiple mobile contexts.
11. "Interestingly, this site is not downstream from a WWTP but is <3 km downstream from a regional hospital."
Is the hospital releasing something to the river? If not, this is irrelevant.
12. "Antibiotic resistance genes (ARGs) have been previously detected in the discharge of wastewater treatment plants (WWTPs) [13, 14, 12], but previous studies have targeted specific resistance determinants or have sampled only one or a few locations."
This is related to comment #5, not fully true. Would be better to tell what you did and why its important than to try understate previous studies. At least state that it is known that ARGs can be found from downstream environments. The bigger question is "What then?"
13. "River samples collected downstream from WWTPs had significantly higher environmental abundance and diversity of ARGs, suggesting that WWTPs can be an important contributor of ARGs to receiving surface waters."
Again, related to previous. This is not so novel finding. And also WWTPs are not the real contributor, but human (or animal) fecal material and actually most WWTPs are part of the solution.
14. "Wastewater treatment can result in substantially reduced total bacterial load, but cells that survive the treatment process are more likely to be antibiotic-resistant (see Karkman et al. 2018 for review)"
I don't think we say this in our review. Due to the high bacterial loads, biofilms and antibiotics found from sewage, WWTPs could be hotspots, but there isn't really any evidence of that. But you can prove me wrong in here, of course. And naturally, I appreciate that you cite our work, and would kindly ask you to add this also to the reference list, now it's only in the text.
15. "In the two largest rivers investigated here, Blue River and Indian Creek, WWTP effluent could comprise over 95 percent of base flow under typical operating conditions [27]"
By reading the reference, I got the impression that it's on average 15 %, but maybe I got something wrong.
16. "These results, combined with the strong correlation of total ARG abundance with the abundance of a human gut phage, is consistent with the interpretation that ARGs were released into surface waters

with human fecal pollution and then diluted in downstream waters"
This has also been shown in other studies, so it would be appropriate to cite these studies here.
17. "This same mobile element was ubiquitous throughout the entire watershed, spanning multiple streamflows, land use types, and pollution levels, suggesting that it may be maintained in natural microbial communities due to a variety of selection pressures"
Could there be another source of ARGs in these upstream sites? Agriculture/animal farming? The numbers in figure 2 are pretty high for non-WWTP sites as well. So it's not probably just environmental background. Or is this an artefact from the normalization?
18. In methods, the authors write: "This smaller size fraction was the focus of the study, as it was considered to be more representative of suspended cells that are dispersing through the watershed rather than being retained in sediments and debris."
Does this mean that 5 Âµm filters were not included in this study? It should be discussed on how this might affect the results. Not all bacteria in environmental samples (flocks/biofilms/cell aggregates) will pass 5 Âµm filter.
19. The approach to use only assembled data has its limitations as the authors do point out. So I would have liked to see read-based analysis done as well. It would have given a better view of the resistome. But the bias is the same for all samples, so the overall message of the manuscript probably won't change.
20. The assemblies could be provided. Assembling all the data to verify the results is bit too much for review purposes. Also, more information about the assemblies could be adde to suppl. table 7: size of assembly, longest contig, mapping rates.
21. The authors have samples 24 sites in triplicates and end up with 74 metagenomes. I don't get the math.
22. I don't understand the discussion about heteroresiostance affecting assemblies. I think bigger problem in trying to assemble antibiotic resistance genes is their location in multiple genetic context. The assembler can't solve these and many times the assembly breaks outside the ARG.
23. Related to the previous comment, the authors have used software called Mix for merging contigs. I don't have experience with it, but as it is designed for WGS of single isolates, I would be very cautious when using it with metagenomes. It might produce chimeric sequences, especially with highly homologous ARGs and MGEs. Mapping reads back to these merged contigs could give some overview of it.

Methods

Are the methods appropriate to the aims of the study, are they well described, and are necessary controls included? Choose an item.

Conclusions

Are the conclusions adequately supported by the data shown? Choose an item.

Reporting Standards

Does the manuscript adhere to the journal's guidelines on [minimum standards of reporting?]() Choose an item.

Choose an item.

Statistics

Are you able to assess all statistics in the manuscript, including the appropriateness of statistical tests used? Choose an item.

Quality of Written English

Please indicate the quality of language in the manuscript: Choose an item.

Declaration of Competing Interests

Please complete a declaration of competing interests, considering the following questions:

- Have you in the past five years received reimbursements, fees, funding, or salary from an organisation that may in any way gain or lose financially from the publication of this manuscript, either now or in the future?
- Do you hold any stocks or shares in an organisation that may in any way gain or lose financially from the publication of this manuscript, either now or in the future?
- Do you hold or are you currently applying for any patents relating to the content of the manuscript?
- Have you received reimbursements, fees, funding, or salary from an organization that holds or has applied for patents relating to the content of the manuscript?
- Do you have any other financial competing interests?
- Do you have any non-financial competing interests in relation to this paper?

If you can answer no to all of the above, write 'I declare that I have no competing interests' below. If your reply is yes to any, please give details below.

I declare that I have no competing interests

I agree to the open peer review policy of the journal. I understand that my name will be included on my report to the authors and, if the manuscript is accepted for publication, my named report including any attachments I upload will be posted on the website along with the authors' responses. I agree for my report to be made available under an Open Access Creative Commons CC-BY license (http://creativecommons.org/licenses/by/4.0/). I understand that any comments which I do not wish to be included in my named report can be included as confidential comments to the editors, which will not be published.

Choose an item.

To further support our reviewers, we have joined with Publons, where you can gain additional credit to further highlight your hard work (see: https://publons.com/journal/530/gigascience). On publication of this paper, your review will be automatically added to Publons, you can then choose whether or not to claim your Publons credit. I understand this statement.

Yes Choose an item.
