# Supplementary material for: Localized effect of treated wastewater effluent on the resistome of an urban watershed
Source: Gigascience. 2020 Nov 19;9(11):giaa125. doi: 10.1093/gigascience/giaa125 (PMC7677451; doi:10.1093/gigascience/giaa125)
Supplement: giaa125_Reviewer_2_Report_Original_Submission — Johan Bengtsson-Palme -- 5/18/2020 Reviewed [file giaa125_reviewer_2_report_original_submission.pdf]

**Reviewer Report**

**Title: Localized effect of treated wastewater effluent on the resistome of an urban watershed**

**Version: Original Submission** **Date:** 5/18/2020

**Reviewer name: Johan Bengtsson-Palme**

**Reviewer Comments to Author:**

The manuscript by Thornton et al. describes a rather large metagenomic study of antibiotic resistance genes (ARGs) in a US watershed. The authors analyze water microbial communities at 24 different sites with differing impact of human activities (mostly in the form of treated WWTP effluent). The methodology and reasoning are overall well thought through and sound, as are the conclusions. While the outcomes of the study are not that spectacularly new - almost all of the findings were pretty much known since before, but in a much more bits-and-pieces way - I like how this study ties all these pieces together and aligns multiple views on resistance dissemination in the environment.
The paper is well written and overall easy to read and addresses an interesting subject. I only have one slightly bigger complaint. As the authors are specifically looking for bacterial resistance genes, I think it would make more sense to normalize to e.g. total Bacterial 16S rRNA or rpoB or similar, as most people studying resistance in the environment do. Instead, the authors invent a new measure of resistance abundance that is hard to understand and compare to anything else. This seems unnecessary and makes the paper hard to compare to existing literature. Otherwise, I think this work is good.
I would recommend the paper for publication after addressing some minor comments and making some clarifications (see below).
Specific comments

- Page 2, last paragraph: How was ARG diversity measured?
- Table 1: I don't understand the units in this table. How is abundance measured? What would an abundance of 1 mean? What is the detection limit? What would 100% resistome correspond to? How is prevalence counted, given that there were only 24 sites? This needs to be much better clarified.
- Page 5, last paragraph before "Potential implications": 1) What Karkman paper is referred to here? I can't find it in the reference list. 2) I am not so sure it is true that WWTPs select for resistant bacteria. The data I have seen suggests no selection for resistance in municipal WWTPs (equally resistant bacteria come in and go out, but in much smaller numbers going out). I think this statement overstates the evidence in one direction.
- Page 6, first paragraph: You can do assembly AND also map the individual genes to an ARG database! You don't have to choose one strategy!
- Page 6, first paragraph under "Sample collection": How does placing the tubing upstream of the sampling equipment minimize contamination?
- Page 8, second paragraph under "Sequence assembly": What measures were investigated to asses assembly performance? That is pretty important to gauge the relevance of this assertion.
- Page 8, first paragraph under "Identification and quantification of ARGs and MGEs": What settings were used for AMRFInder? How was a "true" (high-confidence) resistance gene hit defined?

- Page 9, third full paragraph: I don't get why the last part of the FP calculation is done. Do you assume that you have obtained all possible features from every sample in the assembly? That's an assumption very unlikely to be true. What is the aim of this step and what assumptions is it operating under?

**Methods**

Are the methods appropriate to the aims of the study, are they well described, and are necessary controls included? Choose an item.

**Conclusions**

Are the conclusions adequately supported by the data shown? Choose an item.

**Reporting Standards**

Does the manuscript adhere to the journal's guidelines on minimum standards of reporting? Choose an item.

Choose an item.

**Statistics**

Are you able to assess all statistics in the manuscript, including the appropriateness of statistical tests used? Choose an item.

**Quality of Written English**

Please indicate the quality of language in the manuscript: Choose an item.

**Declaration of Competing Interests**

Please complete a declaration of competing interests, considering the following questions:

- Have you in the past five years received reimbursements, fees, funding, or salary from an organisation that may in any way gain or lose financially from the publication of this manuscript, either now or in the future?
- Do you hold any stocks or shares in an organisation that may in any way gain or lose financially from the publication of this manuscript, either now or in the future?
- Do you hold or are you currently applying for any patents relating to the content of the manuscript?
- Have you received reimbursements, fees, funding, or salary from an organization that holds or has applied for patents relating to the content of the manuscript?
- Do you have any other financial competing interests?
- Do you have any non-financial competing interests in relation to this paper?

If you can answer no to all of the above, write 'I declare that I have no competing interests' below. If your reply is yes to any, please give details below.

I declare that I have no competing interests

I agree to the open peer review policy of the journal. I understand that my name will be included on my report to the authors and, if the manuscript is accepted for publication, my named report including any attachments I upload will be posted on the website along with the authors' responses. I agree for my report to be made available under an Open Access Creative Commons CC-BY license (http://creativecommons.org/licenses/by/4.0/). I understand that any comments which I do not wish to be included in my named report can be included as confidential comments to the editors, which will not be published.

Choose an item.

To further support our reviewers, we have joined with Publons, where you can gain additional credit to further highlight your hard work (see: https://publons.com/journal/530/gigascience). On publication of this paper, your review will be automatically added to Publons, you can then choose whether or not to claim your Publons credit. I understand this statement.

Yes Choose an item.
